# Identification of *Bletilla striata* and related decoction pieces: a data fusion method combining electronic nose, electronic tongue, electronic eye, and high-performance liquid chromatography data

**DOI:** 10.3389/fchem.2023.1342311

**Published:** 2024-01-10

**Authors:** Han Li, Pan-Pan Wang, Zhao-Zhou Lin, Yan-Li Wang, Xin-Jing Gui, Xue-Hua Fan, Feng-Yu Dong, Pan-Pan Zhang, Xue-Lin Li, Rui-Xin Liu

**Affiliations:** ^1^ School of Pharmacy, Henan University of Chinese Medicine, Zhengzhou, China; ^2^ Department of Pharmacy, The First Affiliated Hospital of Henan University of Chinese Medicine, Zhengzhou, China; ^3^ Beijing Zhongyan Tongrentang Medicine R&D Co., Ltd., Beijing, China; ^4^ Henan Province Engineering Research Center for Clinical Application, Evaluation and Transformation of Traditional Chinese Medicine, Zhengzhou, China; ^5^ Co-construction Collaborative Innovation Center for Chinese Medicine and Respiratory Diseases by Henan and Education Ministry of China, Henan University of Chinese Medicine, Zhengzhou, China; ^6^ Henan Provincial Key Laboratory for Clinical Pharmacy of Traditional Chinese Medicine, Zhengzhou, China; ^7^ Engineering Research Center for Pharmaceutics of Chinese Materia Medica and New Drug Development, Ministry of Education, Beijing, China

**Keywords:** *Bletilla striata*, data fusion, electronic senses, feature extraction, PLS-DA, GC-IMS, authenticity, species

## Abstract

**Introduction:** We here describe a new method for distinguishing authentic *Bletilla striata* from similar decoctions (namely, *Gastrodia elata*, *Polygonatum odoratum*, and *Bletilla ochracea schltr*).

**Methods:** Preliminary identification and analysis of four types of decoction pieces were conducted following the *Chinese Pharmacopoeia* and local standards. Intelligent sensory data were then collected using an electronic nose, an electronic tongue, and an electronic eye, and chromatography data were obtained via high-performance liquid chromatography (HPLC). Partial least squares discriminant analysis (PLS-DA), support vector machines (SVM), and back propagation neural network (BP-NN) models were built using each set of single-source data for authenticity identification (binary classification of *B. striata* vs. other samples) and for species determination (multi-class sample identification). Features were extracted from all datasets using an unsupervised approach [principal component analysis (PCA)] and a supervised approach (PLS-DA). Mid-level data fusion was then used to combine features from the four datasets and the effects of feature extraction methods on model performance were compared.

**Results and Discussion:** Gas chromatography–ion mobility spectrometry (GC-IMS) showed significant differences in the types and abundances of volatile organic compounds between the four sample types. In authenticity determination, the PLS-DA and SVM models based on fused latent variables (LVs) performed the best, with 100% accuracy in both the calibration and validation sets. In species identification, the PLS-DA model built with fused principal components (PCs) or fused LVs had the best performance, with 100% accuracy in the calibration set and just one misclassification in the validation set. In the PLS-DA and SVM authenticity identification models, fused LVs performed better than fused PCs. Model analysis was used to identify PCs that strongly contributed to accurate sample classification, and a PC factor loading matrix was used to assess the correlation between PCs and the original variables. This study serves as a reference for future efforts to accurately evaluate the quality of Chinese medicine decoction pieces, promoting medicinal formulation safety.

## 1 Introduction

Bletillae Rhizoma refers to the dried tuber of the plant *Bletilla striata*. It has a long history of use in medicines due to its pharmacological activities, which include hemostatic, gastric mucosal protective, anti-ulcer, antibacterial, anti-inflammatory, and wound healing functions (Li et al., 2014; Posocco et al., 2015; Zhou et al., 2023). Bletillae Rhizoma is also commonly used as a biomedical or cosmetic raw material (Luo et al., 2010; Ding et al., 2016; Jiang et al., 2017; Zhang et al., 2019b), a pharmaceutical excipient (Feng et al., 1995), and a component of industrial glue (Cui et al., 2017; Liao et al., 2019). Continuous discovery of Bletillae Rhizoma functions has gradually expanded its application scope and the market demand. However, *B. striata* plants are fastidious, and their reproductive rates are low under natural conditions (Jiang et al., 2022). Due to excessive collection by humans, wild *B. striata* resources are decreasing every year (Jin et al., 2017). As a result, *B. striata* is now listed as a second-class protected plant in the List of National Key Protected Wild Plants in China (Batch 2) (Zhang et al., 2019a). In recent years, due to high demand and limited availability of high-quality resources, raw *B. striata* materials and decoctions pieces on the market have become heterogeneous in quality. For example, *B. striata* materials are often mixed with the plants *Bletilla ochracea schltr* (Li et al., 2023), *Gastrodia elata*, or *Polygonatum odoratum*, which have some similar characteristics but differ in medicinal value from *B. striata* (Zhai et al., 2012). Thus, circulation of counterfeit *B. striata* products affects the clinical efficacy of *B. striata* decoction pieces and can compromise drug safety. It is therefore necessary to develop efficient, rapid, sensitive detection techniques to measure decoction piece quality.

At present, the primary methods of identifying *B. striata* include traditional manual identification, microscopic analysis, thin-layer chromatography (TLC), near-infrared spectroscopy, and DNA barcoding technology (Cai et al., 2020; Niu et al., 2020). These detection methods are based on modern analysis technologies and exhibit high accuracy, strong reliability, and precise detection. However, some such methods require complex pretreatments and can be prohibitively time-consuming and costly.

Artificial intelligence sensory technologies can be used to quantify multiple quality signals, including sensory information obtained from bionic sensory systems, and to perform pattern recognition for sample classification. This approach provides fast, accurate, comprehensive sample data, and has been widely used in detection and analysis of drugs and foods in the past (Lu et al., 2022; Gui et al., 2023). Data fusion technology consists in merging complementary information to obtain more data points; this technology was originally used in the military, but has gradually been applied in various types of quality evaluation of traditional Chinese medicine such as origin identification (Ru et al., 2019; Wang et al., 2021), species identification (Lan et al., 2020; Sun et al., 2020), quality control of production process (Zhang et al., 2022), and evaluation of preparation quality (Wang et al., 2017; Yan and Sun, 2018). Traditional Chinese medicines are complex in composition and the matrix elements utilized are diverse. Data fusion can organically integrate these types of multi-dimensional data. Furthermore, multi-class intelligent sensory data fusion can simulate traditional manual evaluations by combining visual, auditory, taste, and scent-based data, integrating complementary sensory information to improve identification accuracy. Indeed, several studies have clearly demonstrated the advantages of multi-intelligent sensory data fusion (Zhang et al., 2021b; Li et al., 2022; Hou et al., 2023; Wang et al., 2023).

Data fusion approaches include multiple levels: low, mid, and high. Mid-level fusion can avoid the disadvantages of the large data volumes used in low-level fusion algorithms, effectively reducing data dimensionality (Wang et al., 2019) to highlight key information and facilitate rapid modeling. There are many methods used for feature extraction in mid-level data fusion. In the present study, principal components (PCs) and latent variables (LVs) were used as feature variables and extracted with an unsupervised algorithm [principal component analysis (PCA)] and a supervised algorithm [partial least squares discriminant analysis (PLS-DA)]. Most previous studies have extracted PCs through whole-sample joint dimensionality reduction. However, that dimensionality reduction method reveals information about the validation dataset, jeopardizing accurate analysis of model generalizability. An improved dimensionality reduction method has therefore been developed based on the principle of PCA, enabling isolated dimensionality reduction in the calibration dataset and the validation dataset. This method uses a dimensionality reduction framework (standard deviation and variable boundary) that is consistent between the validation and calibration datasets. Some studies have found that features extracted by supervised algorithms are related to classification labels, which improves identification performance (Xue-Mei et al., 2018; Zhang et al., 2021a). We therefore compared classification results obtained from fused PCA and PLS-DA features.

Here, a mid-level data fusion strategy based on feature extraction was used to identify *B. striata* and related decoction pieces. First, four types of decoction pieces were manually identified based on the *Chinese Pharmacopoeia* and local standards. Differences in the volatile substances present in each type of decoction pieces were analyzed with gas chromatography (GC)–ion mobility spectrometry (IMS). Similarities in high-performance liquid chromatography (HPLC) fingerprints were calculated and response values from an electronic nose, eye, and tongue were analyzed. Using single-source data, models were constructed for authenticity and species identification using PLS-DA, support vector machines (SVM), and back propagation (BP)–neural network (NN) models. Features (PCs and LVs) were extracted using PCA and PLS-DA, respectively. Finally, using the feature fusion data, authenticity and species identification were conducted. Classification performance was compared between the two feature extraction methods and the contribution of each PC to sample identification was analyzed. Finally, the factor loading matrix was calculated for the PCs to explore the feature elements that most strongly influenced sample identification. Overall, our study provides a flexible and accurate method for quality evaluation of *B. striata* and other decoction pieces, promoting medicinal formulation safety.

## 2 Materials and methods

### 2.1 Samples

**FIGURE 1:**
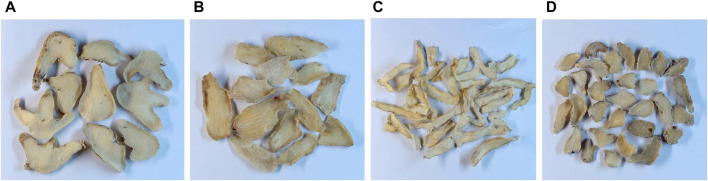
Samples of **(A)** *Bletilla striata*, **(B)** *Gastrodia elata*, **(C)** *Polygonatum odoratum*, and **(D)** *Bletilla ochracea schltr*.

Samples of dried tubers were collected from the Chengdu lotus pond Chinese medicine market and from seven hospitals, including Zhengzhou Hospital of Chinese Medicine, the First Affiliated Hospital of Henan University of Chinese Medicine, the Henan Hospital of Chinese Medicine, and the Zhang Zhongjing Pharmacy. The samples comprised 45 batches of *B. striata* (BS), 30 batches of *G. elata* (GE), 30 batches of *P. odoratum* (PO), and 29 batches of *B. ochracea schltr* (BOS). Each batch consisted of 100 g of material (Figure 1).

### 2.2 Sample identification

#### 2.2.1 Pharmacopoeia- and local standard-based identification

A total of 134 samples were identified with the following methods: appearance analysis; microscopic identification; thin-layer chromatography (TLC) identification; moisture content; and ash content. These detection items were conducted as described in the BS section of the *Chinese Pharmacopoeia* (2020) and the BOS sections of the *Sichuan Provincial Standards for Processing Chinese Herbal Pieces* (2015), the *Gansu Provincial Standards for Processing Chinese Herbal Pieces* (2009), and the *Gansu Provincial Standards for Chinese Medicinal Materials* (2009).

#### 2.2.2 GC-IMS identification

GC-IMS was performed with a FlavourSpec^®^ flavor analyzer (GAS, Germany) equipped with analytical software including Laboratory Analytical Viewer (LAV), Reporter, Gallery Plot, and GC × IMS Library Search. For each sample, 1.0 g of crude powder was accurately weighed and placed in a 20-mL headspace bottle. The powder was incubated at 80°C for 20 min before injection into the flavor analyzer. Each sample was injected twice in parallel. The headspace injection conditions were as follows: incubation temperature, 80°C; incubation time, 20 min; incubation speed, 500 rpm; injection needle temperature, 85°C; injection volume, 500 μL. For GC, an FS-SE-54-CB-1 chromatographic column of 15 m in length was used; the column temperature was 60°C. The carrier gas flow volume was as follows: 0–2 min, 2 mL·min^−1^; 2–20 min, 2–100 mL·min^−1^; and 20–30 min, 100 mL·min^−1^. For IMS conditions, the carrier and drift gas were N_2_, the temperature was 45°C, and the drift gas flow rate was 150 mL·min^−1^. The NIST and IMS databases included with the instrument software were used to identify the volatile organic compounds present in the samples, then the abundances of the volatile organic compounds were analyzed with the Reporter and Gallery Plot functions.

### 2.3 HPLC

Reference standards of militarine and gastrodin (both with purity ≥98%) were obtained from Shanghai Yuanye Biotechnology Co., Ltd (catalog number K18O9B72711) and the National Institute for Food and Drug Control (batch number 110807—201809), respectively. *B. striata* reference medicinal materials were obtained from the National Institute for Food and Drug Control (batch number 121261—201706). Ethanol (Tianjin Yongda Chemical Reagent Co., Ltd.), methanol (Merck KGaA, 64271 Darmstadt, Germany), acetonitrile (Merck KGaA, 64271 Darmstadt, Germany), and phosphoric acid (Beijing DiKMA Technology Co., Ltd.) was chromatographic pure.

For each of the 134 samples, a sufficient volume was crushed through a No. 4 sieve to produce at least 2.0 g powder, which was accurately weighed and placed in a 50-mL stoppered bottle. 40 mL dilute ethanol (concentration: 52.9%) was added to each sample. The total mass was weighed, then ultrasonic extraction was performed for 30 min at 200 W and 40 kHz. Samples were filtered and the filtrate was concentrated until the ethanol was not detectable by taste. The residue was dissolved in 3% acetonitrile in water, centrifuged for 10 min, then filtered through a 0.45 μm microporous membrane. The resulting filtrates were used in HPLC fingerprint analysis, which was performed on UltiMate-3000 HPLC instrument [Thermo Fisher Scientific (ChinaCo., Ltd.] equipped with an ultraviolet detector. The chromatographic column was a Shim-pack GIST C18-AQ (250 mm × 4.6 mm, 5 μm). Mobile phase A was 0.1% aqueous phosphoric acid solution and mobile phase B was acetonitrile. Before use, the mobile phases were degassed and filtered through a 0.2 μm microporous filter membrane. The gradient elution program was as follows: 0–5 min, 5%–20% A; 5–10 min, 20%–24% A; 10–20 min, 24%–31.5% A; 20–25 min, 31.5%–35% A; 25–30 min, 35%–42% A; 30–45 min, 42%–60% A. The flow rate was 1.0 mL/min, the column temperature was 30°C, the injection volume was 10 μL, and the detection wavelength was 280 nm.

### 2.4 Electronic sensory signal acquisition

#### 2.4.1 Electronic nose

Olfactory information was collected using 10 types of metal oxide sensors (W1C, W5S, W3C, W6S, W5C, W1S, W1W, W2S, W2W, and W3S) in a PEN3 portable electronic nose (AIRSENSE, Germany). For each of the 134 samples, there were three replicates of 2 g of powder each. Based on data from our pre-experimental results, the samples were tested after incubating at room temperature in a covered container for 30 min. The sampling time was 80 s, the cleaning time was 80 s, the sensor zeroing time was 5 s, the sample preparation time was 5 s, and the air intake flow rate was 400 mL·min^−1^. The resulting olfactory data were used to construct the olfactory information matrix *X1* (134 × 10).

#### 2.4.2 Electronic eye

The IRIS VA400 electronic eye was used to collect visual data from the samples. Samples of an area of about 10 × 10 cm^2^ were randomly selected and placed on A4 white paper. Top lighting conditions were selected based on pre-experimental results. A 24-color plate was used for color correction. The electronic eye used a 5-mm aperture and the upper and lower backlights were used simultaneously to eliminate the background. Three separate images were taken of each sample with the position of the slices changed between images. The resulting visual data, obtained with 85 sensors, were used to construct the visual information matrix *X2* (134 × 85).

#### 2.4.3 Electronic tongue

Taste information was collected from each of the 134 samples using the TS-5000Z Insent electronic tongue (Ensoul Technology LTD. Electronic tongue sensors include Sourness, Bitterness, Astringency, Aftertaste-B, Aftertaste-A, Umami, Richness and Saltiness.). For each sample, 2 g was weighed out and crushed in an electric homogenizer for 15 s. The resulting powder was placed in a 100-mL beaker with an appropriate volume of purified water. After stirring, samples were incubated at room temperature without perturbation for 5 min. The samples were subsequently filtered, sterilized and poured into a special cup to be tested by the electronic tongue. The electronic tongue sensor was cleaned in a cleaning solution for 90 s, in a reference solution for 120 s, and in a different reference solution for 120 s. The sensor started to collect sample information after the response value stabilized at 0 for 30 s. The acquisition time of the beforetaste value of each sample was 30 s, the sensors were then cleaned for 3 s in the two reference solutions. Finally, the sensors were inserted into the new reference solution to collect data for 30 s and the aftertaste value was exported. This cycle was repeated four times; data from the first cycle were removed and the average value was calculated from the last three cycles. The resulting taste data, obtained from eight sensors, were used to construct the taste information matrix *X3* (134 × 8).

### 2.5 Construction of authenticity and species identification models based on single-source data

To eliminate randomness and ensure model stability, the Kennard-Stone algorithm was used to divide samples of the four species into a calibration set (100 samples) and a validation set (34 samples). The linear classifier PLS-DA, the nonlinear classifier SVM, and BP-NN were used to establish authenticity identification (binary classification) models based on data from the electronic nose, electronic tongue, electronic eye, and HPLC. Model performance was evaluated in terms of accuracy in the calibration set with cross-validation and accuracy in the validation set. Because the program used for SVM could not be used for multi-classification problems, only the PLS-DA and BP-NN algorithms were used to establish multi-class species identification models. The performances of these models were evaluated as described for the authenticity identification model.

PLS-DA is a discriminant method based on partial least squares regression that can be used for dimensionality reduction, classification, and prediction. The algorithm allows determination of whether a given sample belongs to a specific predefined category (Ballabio and Consonni, 2013; Borraz-Martínez et al., 2019). It can transform input data into a set of linear latent variables for classification problems. SVM and BP-NN are nonlinear classifiers based on a kernel function and a large number of neurons, respectively. Both algorithms are widely used in the field of machine learning. SVM is used to identify an optimal decision boundary (a classification hyperplane) and uses a kernel function to map input samples to high-dimensional space for linear separable or non-separable problems (Chauhan et al., 2019). Because the radial basis kernel function of SVM has the advantages of local feature expression and strong learning abilities (Gao et al., 2008), we selected this kernel function. BP-NN is based on abstraction and simulation of the basic characteristics of the human brain or a natural neural network. It includes input, output, and hidden layers. The addition of a momentum term can accelerate the algorithm’s learning speed and avoid the oscillation caused by a high convergence speed (Zheng, 2005). The number of hidden-layer neurons is determined by the specific problem to which it is applied. A greater number of hidden-layer neurons is associated with higher accuracy but reduced generalizability. Furthermore, a smaller number of neurons can reduce the network cost. These parameters usually require a series of repeated tests for optimization.

### 2.6 Feature extraction

Feature extraction was carried out with both PCA and PLS-DA. Improved PCA-based feature extraction was performed in a home-made program designed in MATLAB. First, the PC scores of the calibration samples from each data source were extracted. The validation set was then added to the calibration set for simultaneous standardization of the entire dataset. Standardization was performed with the z-score method (Formula 1):

$$X_{ij}^{\prime }=\frac{X_{ij}-\bar{X_{j}}}{S_{j}},j=1,2,3,\ldots ,m$$ (1)

$X_{ij}$ refers to the number in the *i*th row and the *j*th column. $X_{ij}^{\prime }$ refers to the number after standardization in the *i*th row and the *j*th column; $\bar{X_{j}}$ refers to the average value of the number in *j*th column; $S_{j}$ refers to the standard deviation of the number in *j*th column.

The standardized data in the validation set were then inserted into the linear expression of each PC (Formula 2) to obtain the PC scores of the validation set:

$$Z_{i}=a_{i1}X_{1}^{\prime }+a_{i2}X_{2}^{\prime }+\ldots +a_{im}X_{m}^{\prime }\;i=1,2,\ldots ,m$$ (2)

$Z_{i}$ refers to the scores of *i*th principal component; $a_{i1}$ , $a_{i2}$ , …, $a_{im}$ refers to eigenvector; $X_{1}^{\prime }$ , $X_{2}^{\prime }$ , …, $X_{m}^{\prime }$ refers to the number value of the sample in the first, second, …, *m*th original variables.

For feature extraction based on PLS-DA, the latent variable scores of the calibration set and the validation set were obtained in the process of algorithm operation.

PCA can be used to transform multiple variables into a small number of comprehensive variables, represented by PCs, through dimensionality reduction. The PCs are linear combinations of the original variables that collectively reflect most of the information contained in the original variables. LVs can likewise explain most of the variance of the original data. Here, the PCs selection principle was explanation of more than 90% of the variance in the original data; the selection principle of LVs was determined with leave-one-out cross-validation. The PC and LV scores were used as the input variables for subsequent data analysis.

### 2.7 Construction of authenticity and species identification models with fused data

Using the sample identification results and the features extracted as described in Section 2.6, PLS-DA, SVM, and BP-NN authenticity identification fusion models and PLS-DA and BP-NN species identification fusion models were constructed. Model performance was evaluated as described in Section 2.5.

### 2.8 Model analysis

Wilk’s lambda value represents the ratio of intra-group variation to inter-group variation in a calibration dataset (Yue et al., 2019). A smaller value corresponds to a stronger discriminant ability in a given variable. The factor loading represents the correlation coefficient between PCs and original variables, reflecting the closeness and direction of the relationship between the types of data. Thus, the correlation between each PC and each original variable can be understood through the factor loading matrix. Here, using Wilk’s lambda value, we identified PCs with large contributions to classification in the fusion models. The factor loading of each feature was calculated as follows to analyze the key features affecting sample identification (Formula 3):

$$q_{ij}=\sqrt{\lambda_{i}}a_{ij}$$ (3)

where ${\;\lambda }_{i}$ is the eigenvalue of the *i*th PC and $a_{ij}$ is the coefficient of the original variable $X_{j}$ .

## 3 Results and discussion

### 3.1 Sample identification

#### 3.1.1 Identification based on the pharmacopoeia and local standards

We first analyzed 134 dried tuber samples as set forth in the Pharmacopoeia and with microscopic, TLC, moisture content, and ash content analysis. Based on these analyses combined, 45 batches of *B. striata* decoction pieces met the standards for classification as BS on the *Chinese Pharmacopoeia* (Part I) and did not meet the standards for classification as BOS based on the *Sichuan Provincial Standards for Processing Chinese Herbal Pieces*, the *Gansu Provincial Standards for Processing Chinese Herbal Pieces*, or the *Gansu Provincial Standards for Chinese Medicinal Materials*. Similarly, 30 samples each of GE and PO decoction pieces did not meet the standards for classification as BS; they were positively identified as authentic GE and PO, respectively. However, many of the 29 batches of BOS not only met the standards for classification as BOS, but also met the standards for classification as BS. Specifically, the traits of BOS samples 106–120 were essentially identical to those of BS. However, samples 121–134 were highly wooded and had a significantly different texture than BS, thus meeting local standards for classification as BOS. There was extremely high similarity in TLC results between the 29 BOS batches and the control medicinal samples of BS, making it difficult to distinguish between the two sample types. Microscopic characteristics were consistent between the 29 BOS batches and the BS samples, displaying the characteristic “epidermal cells with wavy curved walls, calcium oxalate needle crystal bundles, catheters, fiber bundles and gelatinized starch granules” described in Pharmacopoeia. However, the microscopic background was more turbid in BOS than in BS samples. Overall, the moisture contents of 134 samples ranged from 6.10% to 12.75% and the ash contents ranged from 1.22% to 4.39%, consistent with expectations based on the Pharmacopoeia.

#### 3.1.2 GC-IMS identification

**FIGURE 2:**
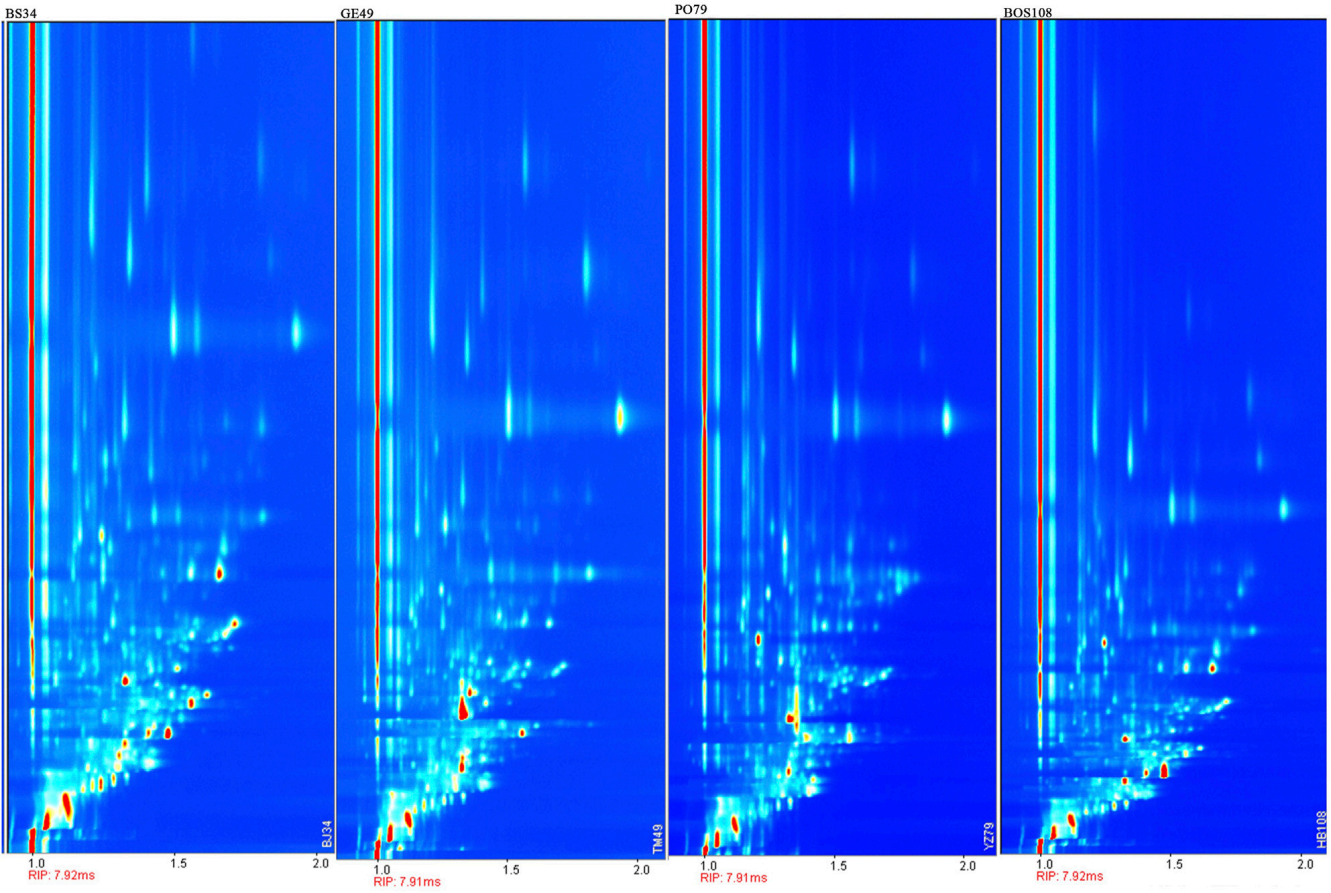
Vertical view plot of four types of decoction pieces [BS, GE, PO, and BOS] based on GC-IMS.

We next analyzed the levels of volatile organic compounds in each sample with GC-IMS. Sample 34 (BS), sample 49 (GE), sample 79 (PO), and sample 108 (BOS) were randomly selected for comparison of the two-dimension vertical view plot of BS with the vertical view plots of the other sample types (Figure 2). In Figure 2, the ordinate represents the GC retention time and the abscissa represents the normalized ion draft time. The red vertical line at abscissa = 1.0 is the normalized reactive ion peak (RIP). Each point on the right side of the RIP peak represents one type of volatile organic compound. Concentrations are indicated with color depth. The results clearly showed significant differences in both the types and abundances of volatile organic compounds between BS and other three types of decoction pieces.

**FIGURE 3:**
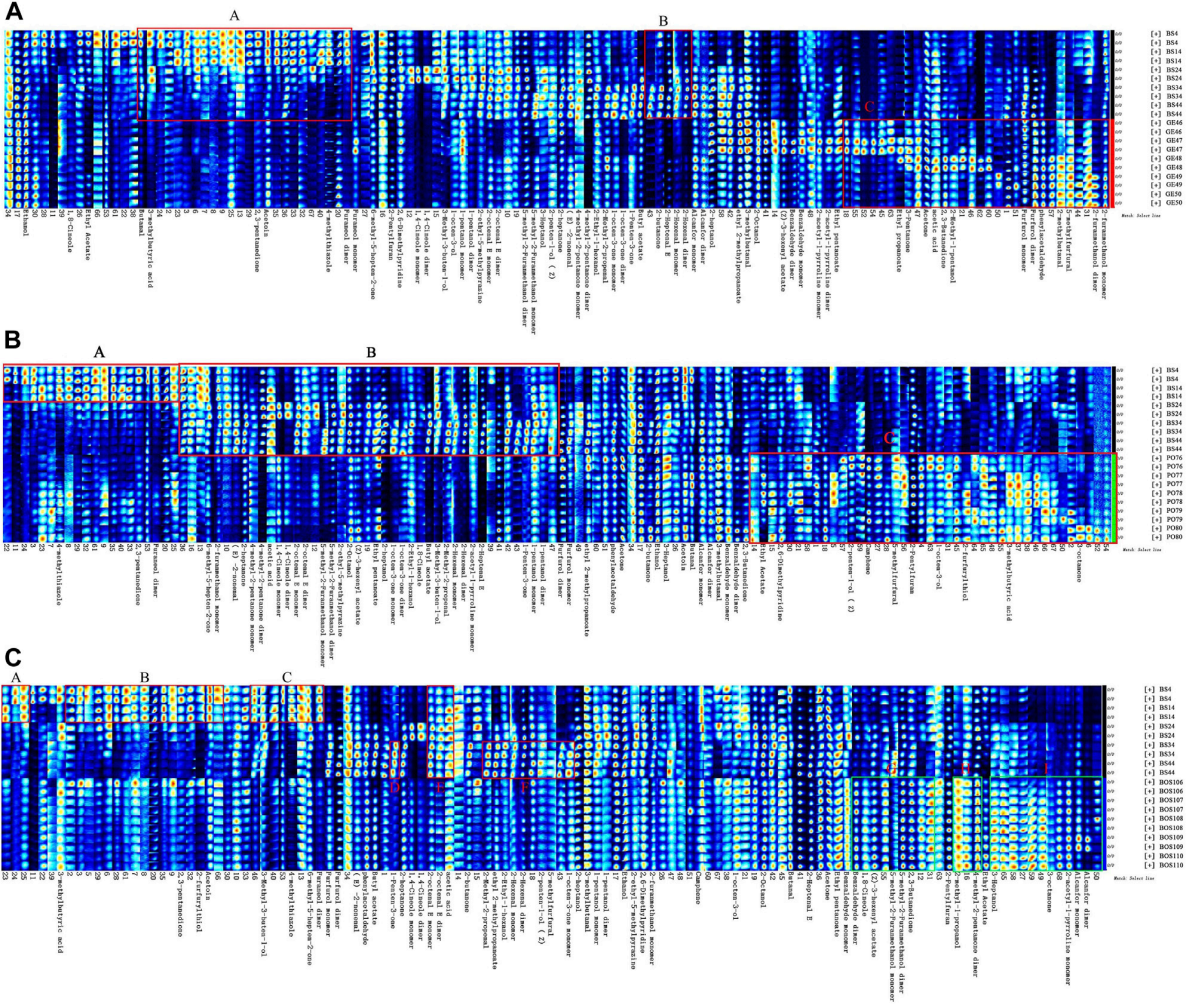
GC-IMS fingerprints of BS compared to **(A)** GE, **(B)** PO, and **(C)** BOS.

We first compared the fingerprints of BS and GE (Figure 3A). In regions A and B, levels of volatile compounds were higher in BS. Specifically, BS had an increased abundance of butyraldehyde, 3-methylbutyric acid, 2,3-pentanedione, 4-methylthiazole, 2-butanone, 2-heptenal, 2-hexenal (monomer), 2-hexenal (polymer), and several unknown compounds compared to GE. Region C shows volatile compounds that were more abundant in GE than in BS, namely, ethyl propionate, 3-pentanone, acetone, acetic acid, 2,3-butanedione, 2-methyl-1-pentanol, furfural (monomer and dimer), phenylacetaldehyde, 2-methylbutanal, 5-methylfurfural, and 2-furanmethanol (monomer and dimer).

Comparison of fingerprints between BS and PO (Figure 3B) showed numerous volatile compounds that were more abundant in BS (regions A and B): 2,3-pentanedione, 4-hydroxy-2,5-dimethyl-3 (2H) furanone (dimer), methylheptenone, furfuryl alcohol (monomer), trans-2-nonenal, 2-heptanone, 4-methyl-2-pentanone (dimer), and some unknown compounds. However, compounds including 1-octen-3-ol, furfuryl mercaptan, cis-2-penten-1-ol, isovaleric acid, 3-octanone, 5-methylfuranaldehyde, camphene, and ethyl acetate were more abundant in PO (region C). Finally, the fingerprints of BS and BOS were compared (Figure 3C). Overall, the volatile organic compounds were similar in both identity and abundance between the two types of decoction pieces, although there were subtle differences. Regions A–F show compounds (including 2,3-pentanedione, furfuryl mercaptan, 3-hydroxy-2-butanone, 3-methyl-3-buten-1-ol, 4-methylthiazole, methylheptenone, 4-hydroxy-2,5-dimethyl-3 (2H) furanone [dimer], and 1-penten-3-one) that were more abundant in BS than in BOS. Regions G–I show compounds that were significantly more abundant in BOS than in BS, including benzaldehyde (dimer), eucalyptol, ethenyl acetate, 5-methyl-2-furanmethanol (monomer and dimer), 2,3-butanedione, isobutanol, and 4-methyl-2-pentanone (monomer). Thus, the integrated results of the Pharmacopoeia, local standards, and GC-IMS positively identified samples 1–45 as BS, samples 46–75 as GE, samples 76–105 as PO, and samples 106–134 as BOS, consistent with the known identities of the samples.

### 3.2 HPLC fingerprinting

**FIGURE 4:**
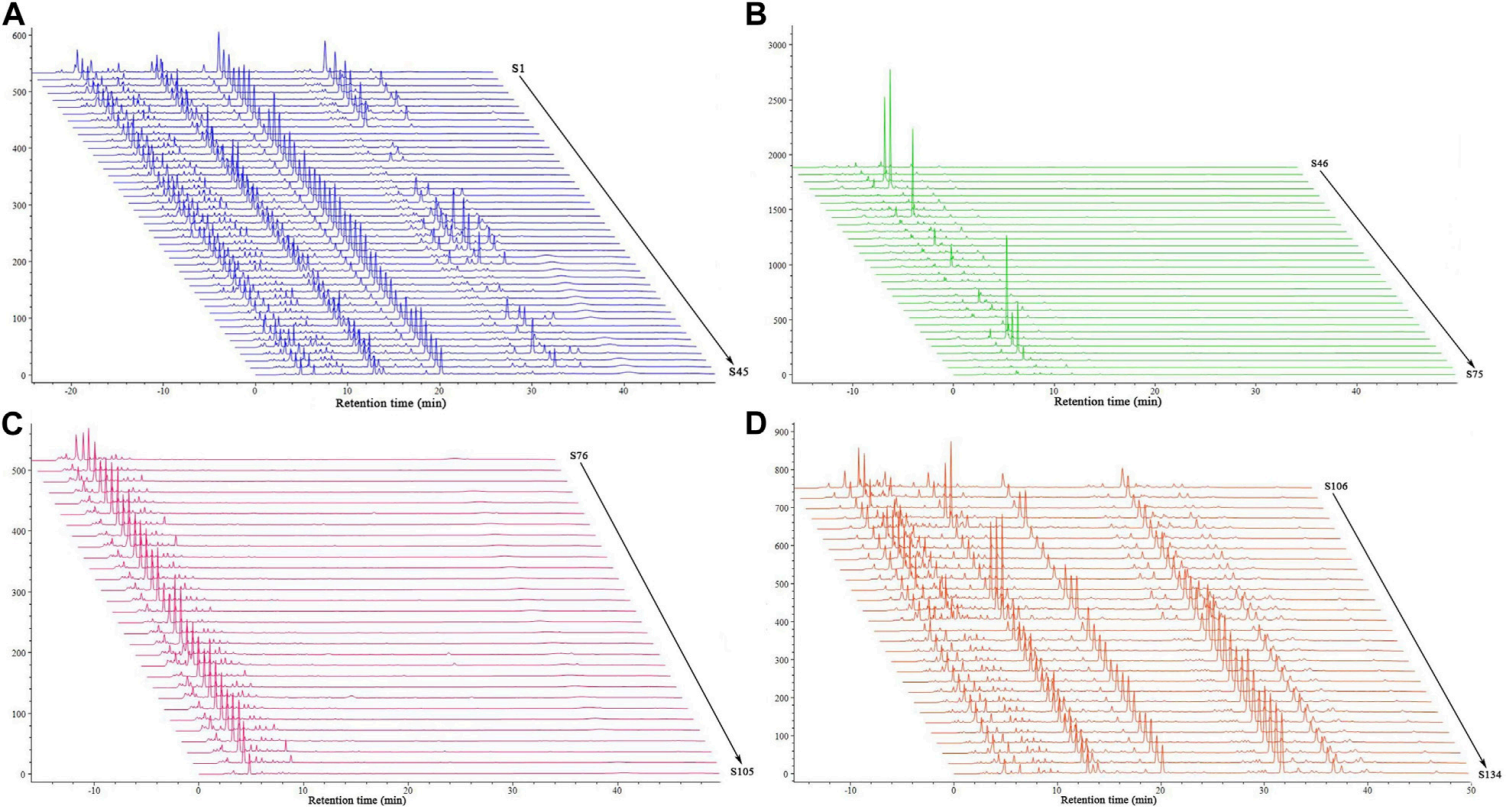
HPLC fingerprints of 134 batches of decoction pieces. Data are shown for **(A)** BA, **(B)** GE, **(C)** PO, and **(D)** BOS.

Each sample was next analyzed with HPLC fingerprinting (Figure 4). Based on reference samples, peak 3 in the control fingerprint (Supplementary Figure S1) of BS was gastrodin and peak 14 was militarine. There were significant differences in the fingerprints of BS, GE, and PO samples. The 45 batches of BS were used as representative samples to generate a control fingerprint. Using the control fingerprint as a reference, the similarities of the 134 batches of decoction pieces were calculated with the common peak weighting method; militarine and other components were weighted as 2.5 and 1, respectively. The fingerprints of the 45 BS, 30 GE, 30 PO, and 29 BOS batches showed similarities of 0.88–0.98, 0.2–0.34, 0.1–0.2, and 0.7–0.88, respectively. Thus, the fingerprint similarities differed considerably between the four types of decoction pieces. Precision: S14 solution was continually injected 6 times according to the above chromatographic conditions. The RSD of relative retention time and relative peak area of 8 common peaks measured with militarine as the reference peak was both ≤0.1%. Repeatability: 6 batches of identical S14 solution were injected according to the above chromatographic conditions. The RSD of relative retention time and relative peak area of 8 common peaks measured by militarine as reference peak were ≤0.1% and ≤2.3%, respectively. Stability: S2 solution was injected at 0, 2, 4, 8, 12 and 24 h after preparation according to the above chromatographic conditions. The RSD of relative retention time and relative peak area of 8 common peaks measured with militarine as the reference peak was 0.1% and 0.3%, respectively, indicating that the sample solution was stable within 24 h.

### 3.3 Electronic sense signals

**FIGURE 5:**
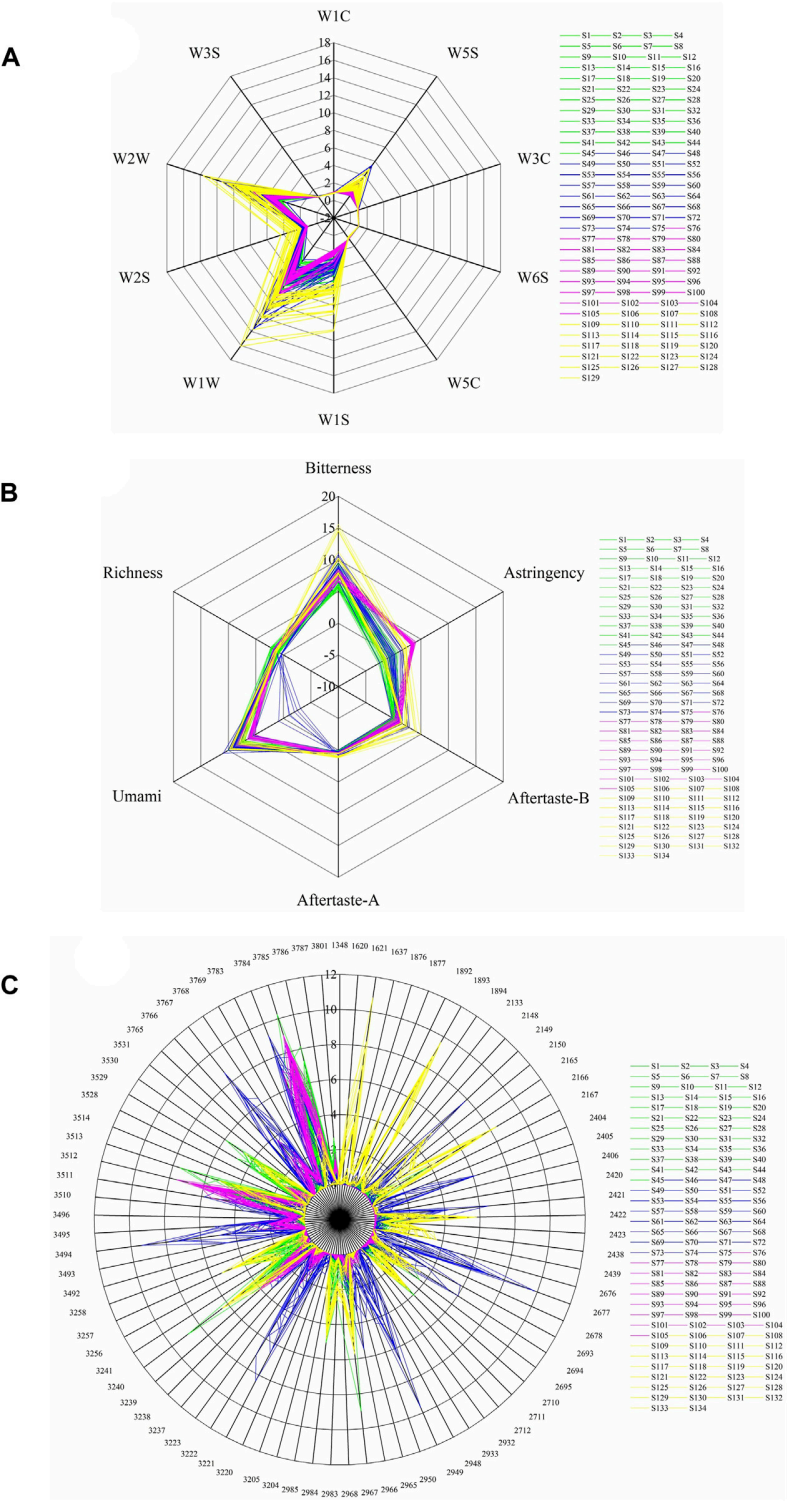
Response values of the electronic **(A)** nose, **(B)** tongue, and **(C)** eye devices for samples of BS (S1–S45), GE (S46–S75), PO (S76–S105), and BOS (S106–S134).

All 134 samples were analyzed with an electronic nose, an electronic tongue, and an electronic eye. Most of the samples had the highest response for the electronic nose sensors W1W, W2W, and W1S, followed by W5S and W2S (Figure 5A). Notably, the response values at sensors W5S and W2S were significantly higher for BOS than for BS samples. Most of the samples had low response values at sensors detecting alkanes and hydrogen gases (e.g., W3S and W6S) and aromatic compounds (e.g., W1C, W5C, and W3C). In the electronic tongue sensor detection, Bitterness, Astringency, Aftertaste-B, and Umami showed the greatest differences between sample types (Figure 5B). The bitterness and astringency response values were relatively low in BS samples but very large in some BOS samples. Other BOS samples showed high similarity to BS samples, indicating extensive heterogeneity among the 29 BOS batches. The results also showed significantly stronger astringency in PO compared to BS samples. Across all samples, the electronic eye detected the strongest color values at numbers 3784, 3785, 3786, 3767, 3494, 3512, 3513, 2677, 3221, 3222, 2949, 2967, and 3240 (Figure 5C). These corresponded to moderate yellow and light yellowish brown sample colors.

### 3.4 Authenticity identification models

#### 3.4.1 Models built with electronic nose data

**FIGURE 6:**
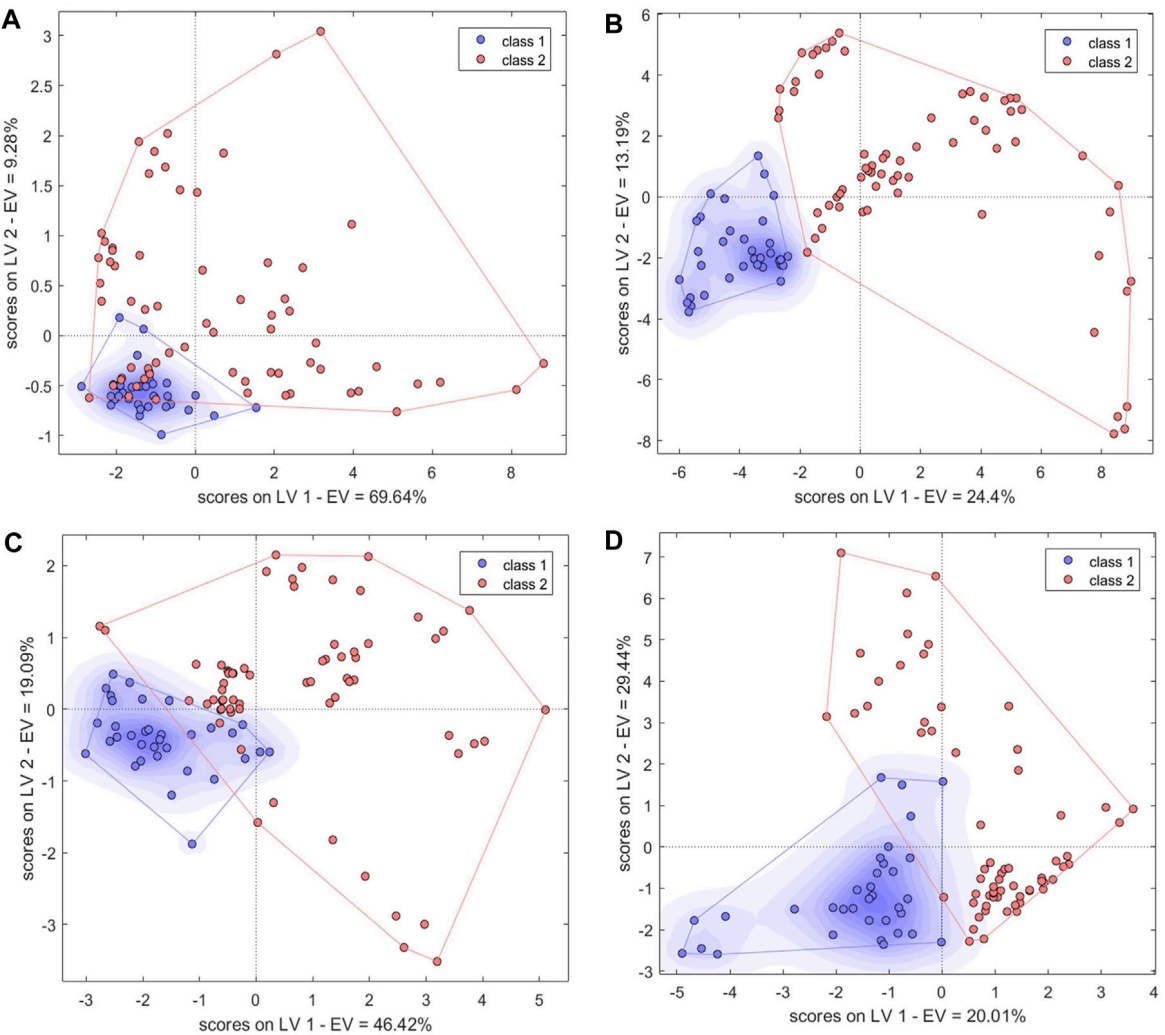
Score plots of single-source PLS-DA authenticity and counterfeit identification model based on **(A)** electronic nose, **(B)** electronic tongue, **(C)** electronic eye and **(D)** high-performance liquid chromatography. Class1, BS; class2, counterfeit species (GE, PO, and BOS).

The PLS algorithm was used to convert the electronic nose data into linear LVs. Six LVs were then used to establish a PLS-DA model with good performance. These first six LVs explained 99% of the total variance among the samples. For the first two LVs, there were some similarities between BS and counterfeit samples (Figure 6A). In the calibration set, the BS samples S34 and S44 were misclassified, and S76 (PO) was misclassified as BS (Table 1). In the validation set, S57 (GE) was misclassified as BS. Misclassification of the samples may have been due to differences in volatile components compared to other samples resulting from changes in temperature and humidity during transportation and storage. Overall, the model accuracy was 97% in the calibration set and 97.06% in the validation set. The model sensitivity (*Se*) and specificity (*Sp*) were 0.94 and 0.98, respectively. This indicated that electronic nose data could be used to accurately distinguish between BS and similar decoction pieces.

**TABLE 1:** Authenticity identification results and model parameters [*Se* = TP/(TP + FN); *Sp* = TN/(TN + FP); *Ac* = TP/(TP + FP); TP: true positive; FP: false positive; TN: true negative; FN: false negative].

Model	Data matrix	Calibration set					Validation set	
		Misclassified samples	Not-assigned samples	Se	Sp	Ac	Misclassified or not-assigned samples	Ac
PLS-DA	EN	3	0	0.9400	0.9800	0.9700	1	0.9706
	EE	2	0	1.0000	0.9700	0.9800	1	0.9706
	ET	1	0	0.9700	1.0000	0.9900	1	0.9706
	HPLC	2	0	0.9700	0.9800	0.9800	0	1.0000
	**Data fusion by PCs**	**0**	**0**	**1.0000**	**1.0000**	**1.0000**	**1**	**0.9706**
	**Data fusion by LVs**	**0**	**0**	**1.0000**	**1.0000**	**1.0000**	**0**	**1.0000**
SVM	EN	8	0	0.9100	0.9200	0.9200	4	0.8824
	EE	1	0	1.0000	0.9800	0.9900	1	0.9706
	ET	10	0	1.0000	0.9800	0.9000	2	0.9412
	HPLC	6	0	0.8500	0.9800	0.9400	0	1.0000
	**Data fusion by PCs**	**0**	**0**	**1.0000**	**1.0000**	**1.0000**	**1**	**0.9706**
	**Data fusion by LVs**	**0**	**0**	**1.0000**	**1.0000**	**1.0000**	**0**	**1.0000**
BP-NN	EN	2	0	0.9400	1.0000	0.9800	1	0.9706
	**EE**	**1**	**0**	**1.0000**	**0.9800**	**0.9900**	**1**	**0.9706**
	ET	1	1	1.0000	0.9800	0.9800	1	0.9706
	HPLC	5	0	0.9100	0.9700	0.9500	0	1.0000
	**Data fusion by PCs**	**1**	**0**	**1.0000**	**0.9800**	**0.9900**	**1**	**0.9706**
	**Data fusion by LVs**	**1**	**0**	**1.0000**	**0.9800**	**0.9900**	**1**	**0.9706**

The bold values indicate the optimal model.

We next optimized the kernel parameter and cost values, which were initially 0.05 and 0.1, respectively (Supplementary Figure S2). The error rate in cross-validation changed along with the kernel parameters and cost values, and we selected the combination that produced the lowest error rate and highest accuracy (kernel parameter = 1.13, cost value = 10). The model was run again with the optimized parameters. In the calibration set, S28, S34, and S44 (BS) were misclassified, whereas S64 (GE), S83, S86, and S101 (PO), and S121 (BOS) were misclassified as BS; in the validation set, S30, S81, S82, and S128 were misclassified. Thus, the accuracy was 92% and 88.24% in the calibration and validation sets, respectively. The model *Se* and *Sp* were 0.91 and 0.92, respectively. Visualization of the SVM classification hyperplane and decision boundaries (Supplementary Figure S3A) indicated that the performance of the model built with electronic nose data required improvement.

**FIGURE 7:**
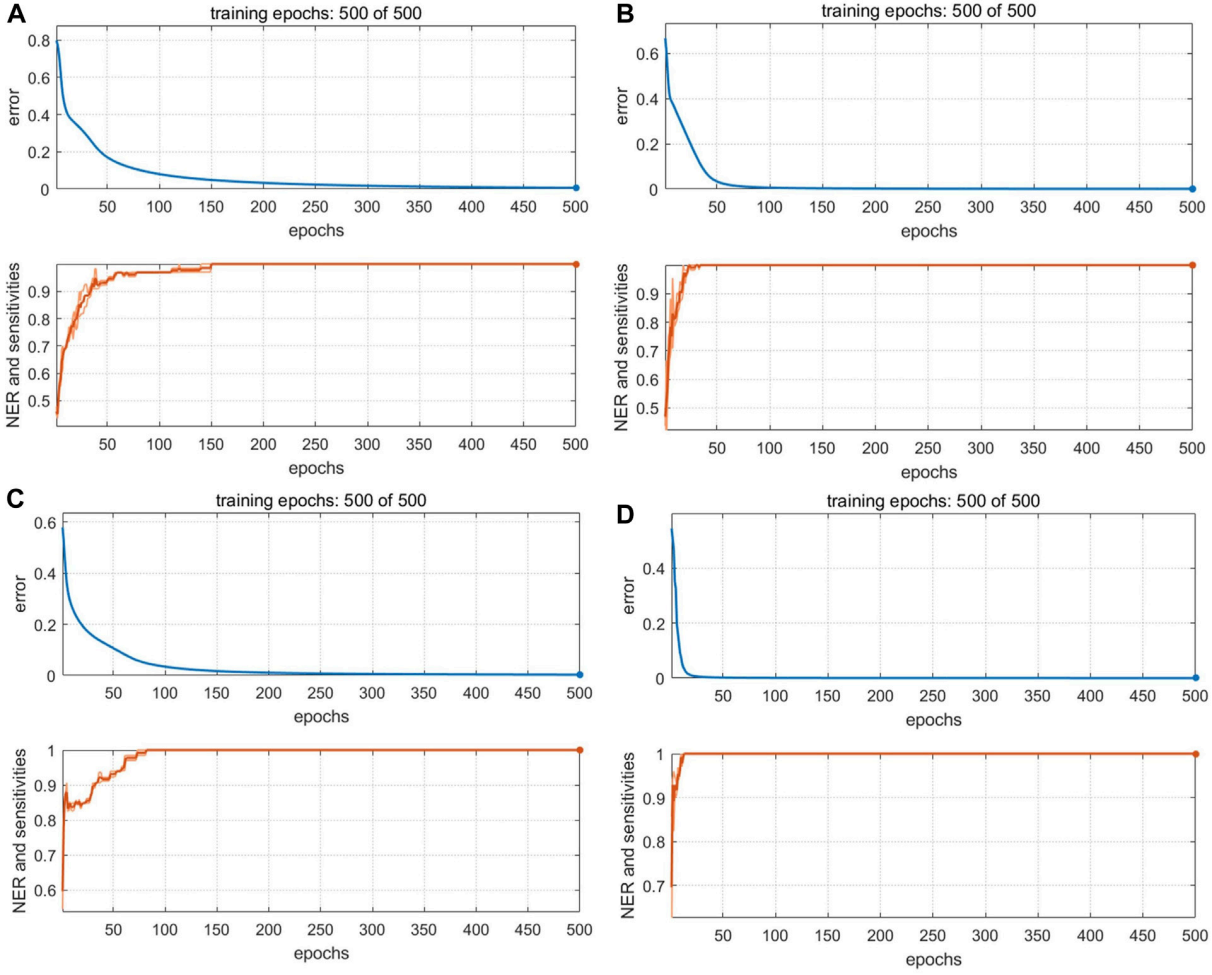
Training iterations and error rates of BP-NN authenticity and counterfeit model. Models were built with data from **(A)** an electronic nose, **(B)** an electronic eye, **(C)** an electronic tongue, and **(D)** HPLC.

The optimized BP-NN model parameters were as follows: two hidden layers; 10 neurons per layer; learning rate = 0.01; momentum term = 0.5; 500 iterations. After 500 iterations of training, the model error rate was 0 (Figure 7A). In the calibration set, the BS samples S28 and S44 were misclassified; in the validation set, S82 was unassigned. Notably, these three samples were also misclassified or unclassified in the PLS-DA and SVM models, presumably due to differences in sample quality. The accuracy was 98% and 97.06% in the calibration and validation sets, respectively, and the *Se* and *Sp* were 0.94 and 1.0, respectively. Thus, the BP-NN model built with electronic nose data could be used to accurately distinguish between BS and related decoction pieces.

#### 3.4.2 Models built with electronic eye data

In leave-one-out cross-validation, the PLS-DA model performed best when using the first three LVs, which together explained 63% of the sample variance. The two types of samples (BS and others) had good aggregation in the first two LVs (Figure 6B). S84, S91, and S5 were misclassified. Analysis of the original samples suggested that they may have been misclassified because they had slightly different surface color characteristics compared with other samples. The accuracy was 98% and 97.06% in the calibration and validation sets, respectively, and the *Se* and *Sp* were 1.0 and 0.97, respectively.

The SVM model had the lowest error rates when the kernel parameter was 1.6, 2.26, 3.2, or 4.53 and the corresponding cost value was 1, 1, 1, or 0.1, respectively; these parameter combinations produced accuracies of 96%, 99%, 99%, and 96%, respectively. Larger kernel parameter and cost values were associated with smaller numbers of support vectors (Supplementary Figure S4). The role of a support vector is to determine the optimal classification hyperplane. The number of support vectors is affected by both the penalty parameter *c* and the kernel parameter *g* (Yang and Gao, 2020). A larger *c* corresponds to fewer support vectors, higher model accuracy, a smaller occupied network space, and faster prediction speeds. The number of support vectors was smallest (51) when the kernel parameter was 3.2 and the cost value was 1. This model was therefore selected for further analysis. Using these parameters, S91 and S5 were misclassified in the calibration and validation sets, respectively; *Ac* was 99% and 97.06% in the calibration and validation sets, respectively; *Se* and *Sp* were 1.0 and 0.98, respectively.

Because there were 85 color values detected by the electronic eye and the input variables were large, the number of hidden-layer neurons in the BP-NN model needed to be optimized for these data. Too many hidden-layer neurons will weaken model generalizability and increase network operation costs. After optimization, the parameters were as follows: three hidden layers; 10 neurons per layer; learning rate = 0.01; momentum term = 0.5; 500 iterations. When the number of training iterations was 500, the model error rate was 0 (Figure 7B). However, S5 was still misclassified in the validation set. This sample comprised irregular thick slices with sections of white, gray-white, and brown; thus, the sample characteristics were different from the irregular thin slices and white color of the 44 other BS samples. In the validation set, S116 was misclassified. *Ac* was 99% and 97.06% in the calibration and validation sets, respectively and the model *Se* and *Sp* were 1.0 and 0.98, respectively.

#### 3.4.3 Models built with electronic tongue data

The PLS-DA model performed best when it was built with four LVs that explained 83% of the sample variance (Figure 6C). S3 and S60 were misclassified in the calibration and validation sets, respectively. BS and non-BS samples showed some similarities in the variance of the first two LVs. The *Se* and *Sp* of the model were 0.97 and 1, respectively, and the classification performance was good.

In cross-validation, the SVM model error rate was smallest when the kernel parameter was 0.4, 0.57, 0.8, 1.6, or 3.2, and the corresponding cost value was 1, 1, 1, 10, or 1, respectively. Model accuracy was highest (90% and 94.12% for the calibration and training sets, respectively) at kernel parameter = 0.57 and cost value = 1. Using this model, there were seven and three misclassified BS and GE samples, respectively, in the calibration set. In the validation set, S10 and S67 were misclassified. *Se* and *Sp* were 1.0 and 0.98, respectively. The classification result showed that when the SVM classifier performed pattern recognition on the electronic tongue data, the relationship between the recognition mechanism and the differences in electronic tongue data demonstrated a need for further optimization.

The optimal BP-NN model contained two hidden layers and five neurons per layer; it had a learning rate of 0.01, a momentum term of 0.3, and was trained for 500 iterations. In the calibration set, the accuracy was 98%, S15 was unassigned, and S53 was misclassified. S48 was misclassified in the validation set (*Ac*: 97.06%). After 500 iterations of training, the error rate was 0 (Figure 7C). Model accuracy increased the most when the momentum term was gradually increased from 0.2 to 0.5 while other parameters were unchanged. However, when the momentum term was increased from 0.4 to 0.5, the accuracy decreased due to an increase in the number of unclassified samples. This indicated that a larger momentum term was not well suited for model classification and that parameters in the BP-NN model should be further optimized. The model *Se* and *Sp* were 1.0 and 0.98, respectively.

#### 3.4.4 Models built with HPLC data

The first 12 LVs, which could explain 89% of the sample variance, were selected to construct a PLS-DA model. In the calibration set, S41 and S119 were misclassified, but the accuracy in the validation set was 100% (Figure 6D). The model *Se* and *Sp* were 0.97 and 0.98, respectively, and the classification performance was good. For the SVM model (Supplementary Figure S3D), accuracy was highest (94% and 100% in the calibration and validation sets, respectively) when the kernel parameter was 9 and the cost value was 1. The model *Se* and *Sp* were 0.85, 0.98, respectively. The optimized BP-NN model contained two hidden layers and 10 neurons per layer; the learning rate was 0.1, the momentum term was 0.5, and it was trained for 500 iterations, producing an error rate of 0 (Figure 7D). There were five misclassified samples in the calibration set with *Ac* of 95%, but the validation set accuracy was 100%. The model *Se* and *Sp* were 0.91, 0.97, respectively. The high accuracy in the validation set may have been because there were extensive differences in the HPLC data between the four types of decoction pieces; especially when the sample size was small, this could correspond to clear difference between sample types. Thus, for the HPLC data, the accuracies of both the linear and nonlinear classifiers were 100% on the validation set.

#### 3.4.5 Models built with mid-level fused data

**FIGURE 8:**
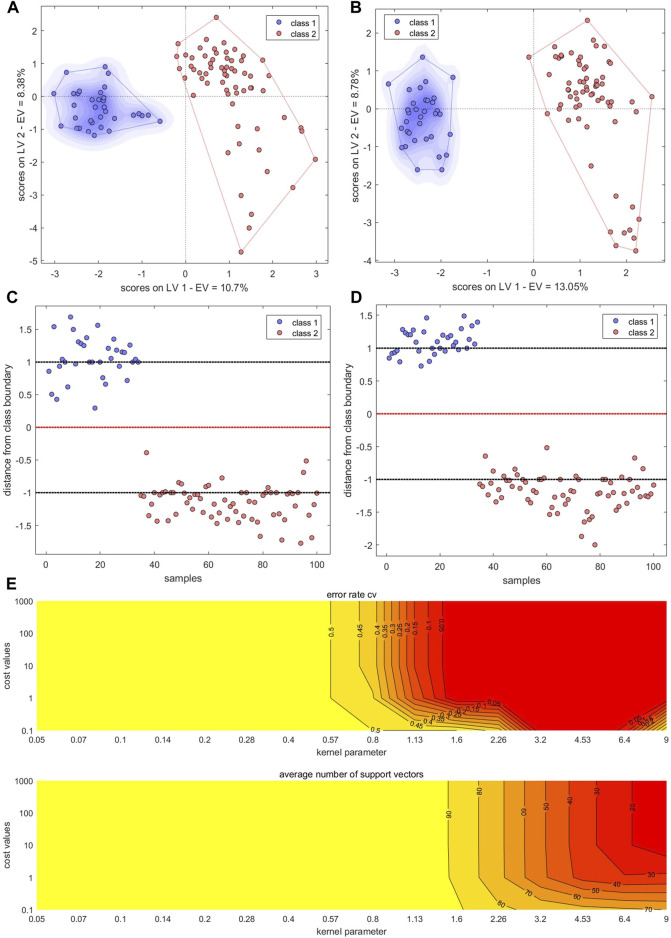
Results of models built with fused data. **(A,B)** PLS-DA score plots. **(C,D)** SVM classification hyperplanes and support vectors. **(E)** SVM parameter optimization. Models shown in **(A,C)** were built with fused PCs; models shown in **(B,D,E)** were built with fused LVs.

**FIGURE 9:**
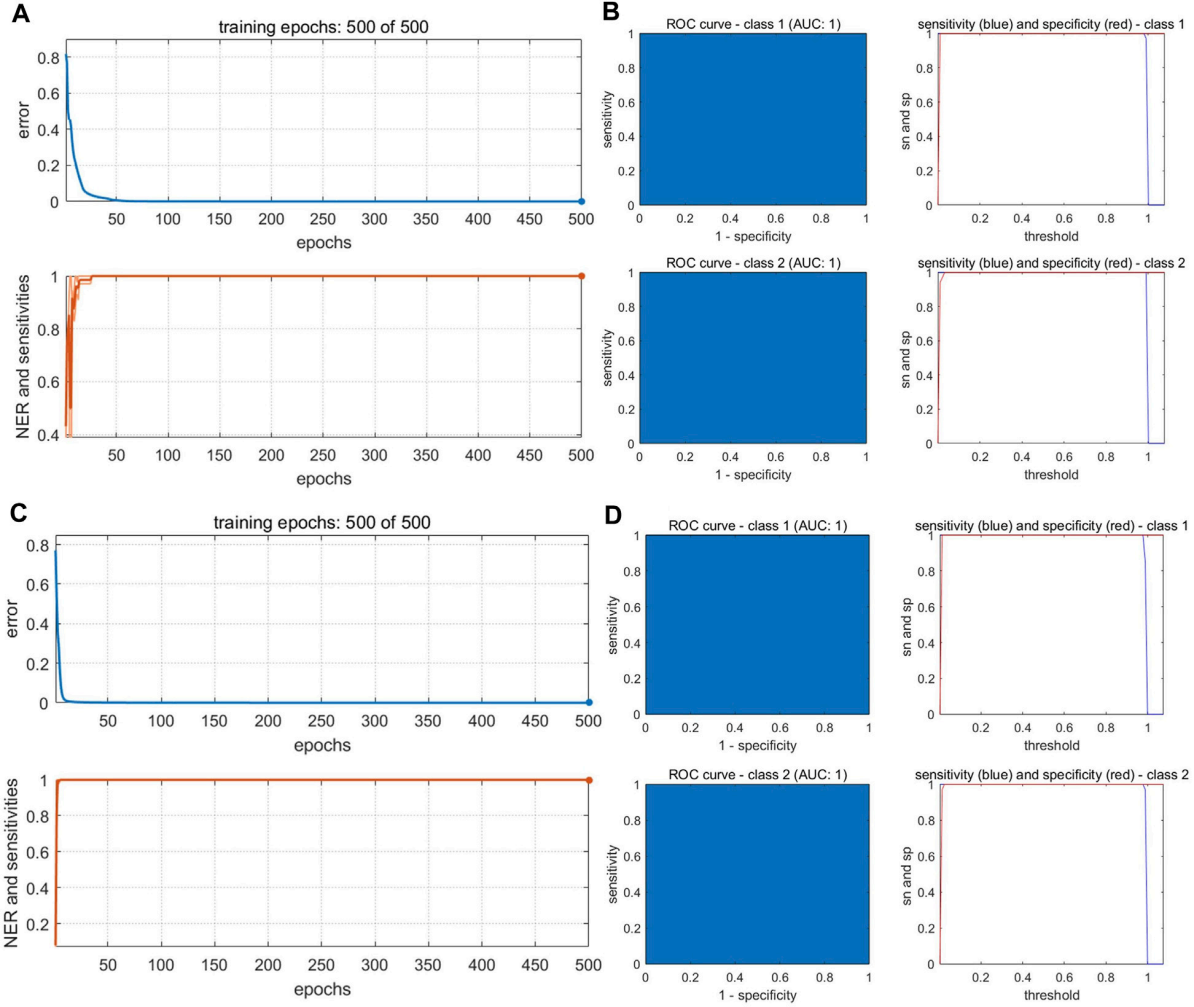
Performance of BP-NN authenticity and counterfeit model. **(A)** Training iterations and error rates for a model built with fused PCs. **(B)** Receiver operating characteristic (ROC) curves (left), sensitivity, and specificity (right) for a model built with fused PCs. **(C)** Training iterations and error rates for a model built with fused LVs. **(D)** ROC curves (left), sensitivity, and specificity (right) for a model built with fused LVs.

The PC scores of the electronic sensor and HPLC data were fused and models were built as described above. The accuracy of the resulting PLS-DA model was 100% for the calibration set, which was superior to the performance of the PLS-DA models constructed with single-source data. In the validation set, only S5 was misclassified (*Ac*: 97.06%). *Se* and *Sp* were 1.0, 1.0, respectively. The two sample types could be completely divided into two categories based on the first two LVs (Figure 8A); the aggregation trend was also superior compared to the models built with single-source data. This indicated that fused PCs (each explaining more than 90% of sample variance in the single-source data) could be used to accurately identify the two sample types while reducing the algorithm running speed. The optimal SVM model had a kernel parameter of 6.4 and cost value of 1; this produced a classification prediction probability of 1 for both sample types (Supplementary Figure S5), which was better than the corresponding SVM model for each single-data source. Model accuracy was also 100% when the kernel parameter was 2.26 or 9 and the cost value was 1, but the number of support vectors was larger in the former case and the classification prediction probability was lower in the latter case. Thus, a kernel parameter/cost value combination of 6.4/1 was selected. The classification hyperplane and support vectors of SVM model was shown in Figure 8C. In the validation set, S5 was misclassified (*Ac*: 97.06%). *Se* and *Sp* were 1.00, 1.00, respectively. The optimal BP-NN model built with the fusion data contained two hidden layers and 10 neurons per layer with a learning rate of 0.1, a momentum term of 0.4. It was trained for 500 iterations, producing a final error rate of 0 (Figure 9A). The area under the curve (AUC) of the receiver operating characteristic (ROC) was 1 (Figure 9B). In the calibration set, S120 was misclassified with *Ac* of 99.00%; S5 remained misclassified in the validation set with *Ac* of 97.06%. The model *Se* and *Sp* were 1.00, 0.98, respectively.

After fusing the LVs from each data source, all three model types could discriminate between samples better than the corresponding models trained on single-source data and performed comparably to or better than the models built with PC fusion data. The PLS-DA model showed 100% accuracy in both the calibration and validation sets, and *Se* and *Sp* were both 1. The two sample types could be completely separated on the first two LVs, and samples clustered together better than in the results of the models built with PC fusion data (Figure 8B). For SVM, the optimal parameter combination was kernel parameter = 9 and cost value = 1 (Figure 8E); this model had 33 support vectors (Figure 8D) and accuracy was 100% on both the calibration and validation sets. *Se* and *Sp* were 1.00, 1.00, respectively. The optimized BP-NN model had two hidden layers, 10 neurons per layer, a learning rate of 0.1, a momentum term of 0.3, and 500 iterations (final error rate = 0) (Figure 9C). Using this parameter combination, only S76 was misclassified. The AUC of ROC was 1, and the *Se* and *Sp* values at varying prediction probability thresholds showed that the *Sp* value increases when it is close to 0 and the *Se* value decreases when it is close to 1 and the area surrounded by the red and blue lines is also large, which indicated that the model had good classification performance (Figure 9D). The accuracy of the final model was 99%, with only one sample misclassified in the validation set and the model *Se* and *Sp* were 1.00, 0.98, respectively; this was superior to the classification results based on electronic nose or electronic tongue data alone.

### 3.5 Species identification

#### 3.5.1 Models built with electronic nose data

**FIGURE 10:**
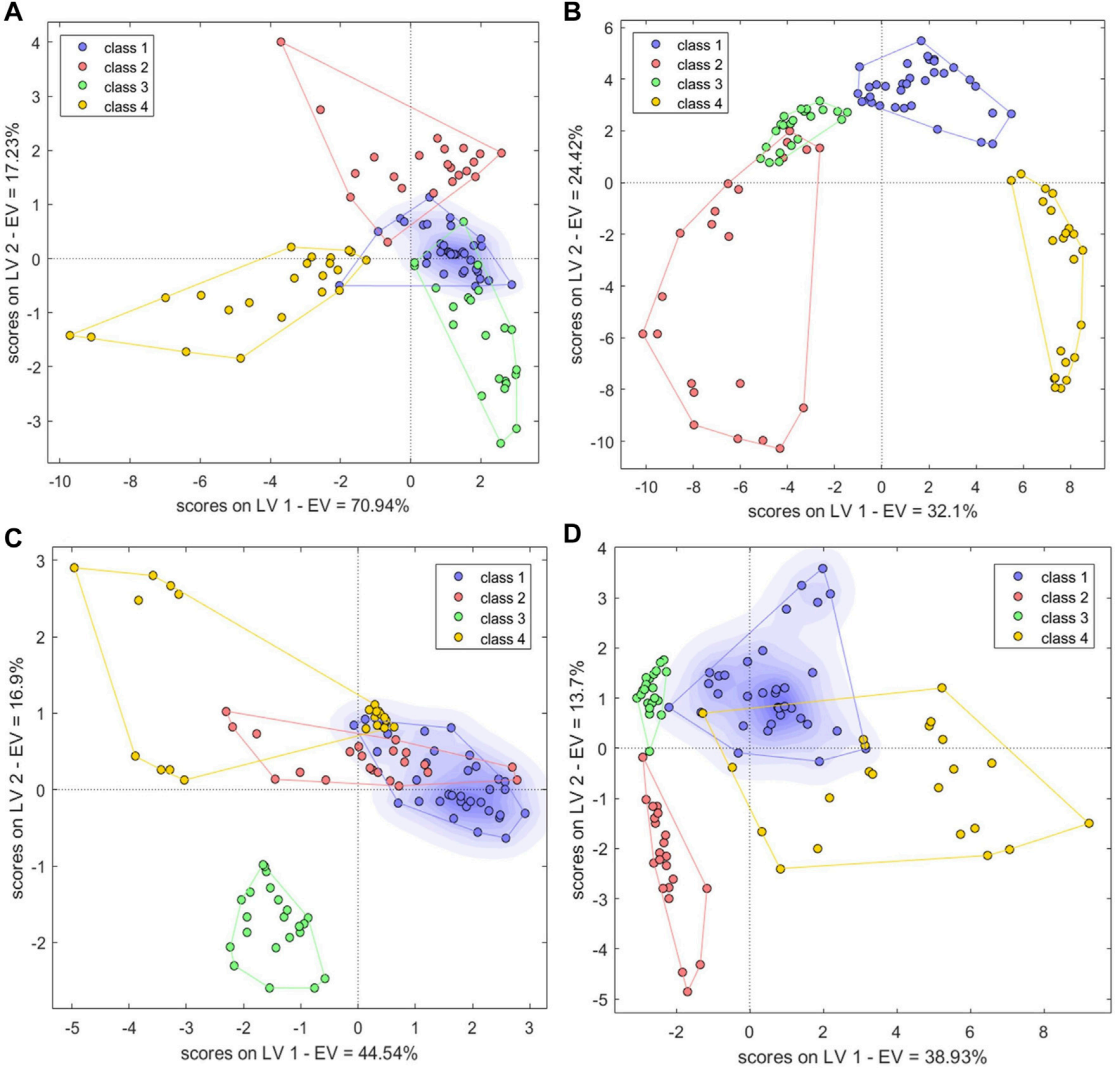
Single-source PLS-DA score plots of species identification model based on **(A)** electronic nose, **(B)** electronic tongue, **(C)** electronic eye and **(D)** HPLC. Class1, BS; class2, GE; class3, PO; class4, BOS.

Six LVs which can explain 99% of the sample variance were selected for construction of an optimal PLS-DA species identification model. The four sample types showed similarities in the variance represented by the first two latent variables (Figure 10A). S28 and S34 were misclassified in the calibration set. Because PLS-DA can divide samples only into predefined categories, this model was unable to identify specific samples; one, one, and four BS, GE, and PO samples, respectively, were unassigned. The accuracy was 92% in the calibration set, and *Se* and *Sp* were 0.94 and 1, respectively. There were four unassigned samples in the validation set. This indicated a need for further improvement of this model.

The model identification criteria were strict in the BP-NN model because the samples were divided into four categories. This necessitated an appropriate increase in the number of hidden layers and the number of neurons in each layer. The final model contained three hidden layers with 10 neurons per layer and had a learning rate of 0.1, a momentum term of 0.3, and was trained for 500 iterations. In the calibration set, S28 was misclassified and S80 and S83 were unclassified and *Ac* was 97.00%. The model *Se* and *Sp* were 0.97, 1.00, respectively. In the validation set, two samples were misclassified and the overall accuracy was 94.12%.

#### 3.5.2 Models built with electronic eye data

The first 13 LVs, which explained 95% of the sample variance, were selected to establish a PLS-DA model. The four sample types could be clearly distinguished in the two-dimensional plot, with GE and PO clustering relatively close to one another and BS and BOS samples also clustering near one another (Figure 10B). In the calibration set, S52, S63, and S91 were misclassified and seven samples were unclassified (*Ac*: 90%). In the validation set, S5, S11, and S48 were unassigned and S67 and S93 were misclassified (*Ac*: 85.29%). *Se* and *Sp* were 1.00, 0.98, respectively.

For the BP-NN model, the optimal parameter combination was two hidden layers, 15 neurons per layer, a learning rate of 0.1, a momentum term of 0.3, and 500 training iterations. In the calibration set, the accuracy was 98%; S23 was unclassified and S69 was misclassified. The model *Se* and *Sp* were 1.00, 1.00, respectively. The AUC-ROC was 1, indicating good parameter optimization (Supplementary Figure S6). Increasing the number of hidden layers from two to three while keeping the other parameters unchanged led to a sudden increase in the number of unclassified samples. This suggested that the multi-classification ability of the model could not be improved overall by only increasing the number of hidden layers.

#### 3.5.3 Models built with electronic tongue data

To some extent, taste responses reflected the chemical composition of each sample. The electronic tongue data could therefore theoretically characterize the differences between the four types of decoction pieces better than the other sensory data types. Seven LVs accounting for a full 100% of the sample variance were used to build a PLS-DA model. Compared to the other two sensory data types, the number of unassigned samples decreased to one GE and two PO samples. There were no misclassified samples. *Ac* was 97.00% and 100% in the calibration and validation sets, respectively and *Se* and *Sp* were 1.0 and 1.0, respectively. Clustering based on just the first two LVs also showed good sample separation (Figure 10C); PO samples clustered far away from the other three sample types. BS, GE, and some BOS samples clustered close together, indicating that their tastes were similar. The clear distinction of the latter three sample types from PO was likely because PO is a member of the family *Liliaceae*, whereas the other three belong to the family *Orchidaceae*.

The optimal BP-NN model contained two hidden layers with five neurons per layer; the learning rate was 0.1, the momentum term was 0.4, and there were 500 iterations. In the calibration set, S29 and S53 were misclassified and S24, S79, S114 were unclassified (*Ac*: 95%). In the validation set, the accuracy was 97.06% and there was only one unassigned sample. The model *Se* and *Sp* were 0.97, 0.98, respectively. The error rate reached 0 after 500 iterations and the AUC-ROC was 1 (Supplementary Figure S7). This indicated that the selected parameters were appropriate for distinction between the four sample types.

#### 3.5.4 Models built with HPLC data

A PLS-DA model was constructed from the first nine LVs, which explained 84% of the sample variance. There were four misclassified and four unclassified samples in the calibration set (*Ac*: 92%); in the validation set, the accuracy was 100%. *Se* and *Sp* were 0.94, 0.98, respectively. In the two-dimensional plot, the BS and BOS samples overlapped, whereas GE and PO samples could be clearly classified into two separate categories, distinct from the BS and BOS samples (Figure 10D). This was consistent with the high similarity between BS and BOS samples in the HPLC fingerprint data. The optimal BP-NN model built on these data had two hidden layers, 10 neurons per layer, a learning rate of 0.1, a momentum term of 0.5, and 500 iterations. There were nine misclassified samples in the calibration set, but the accuracy was 100% in the validation set. The model *Se* and *Sp* were 0.94, 0.97, respectively.

#### 3.5.5 Models built with mid-level fused data

**FIGURE 11:**
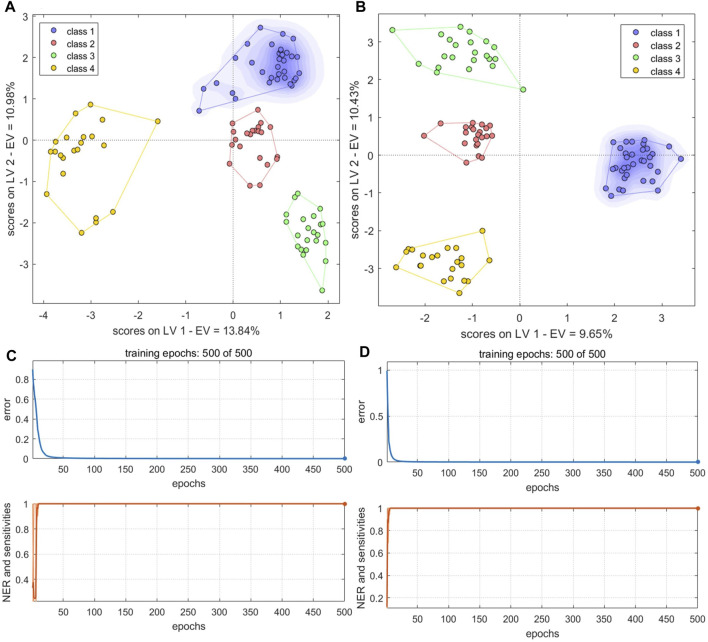
PLS-DA score plots and the number of training iterations and error rates for models of species identification. **(A,B)** PLS-DA score plots for models built with fused PCs **(A)** and LVs **(B) (C,D)** Training iterations and error rates of BP-NN model built on fused PCs **(C)** and LVs **(D)**. Class1, BS; class2, GE; class3, PO; class4, BOS.

The PCs from the electronic sensor data were next fused with the PCs from HPLC data (Table 2). PLS-DA and BN-NN models built with the fused data had improved classification abilities compared to the corresponding models built with single-source data. The PLS-DA model was constructed with the first five LVs. The accuracy in the calibration set was 100%; in the validation set, S5 was still misclassified (*Ac*: 97.06%). *Se* and *Sp* were 1.00, 1.00, respectively. In the variance represented by the first two LVs, the four sample types could be clearly distinguished, although BS and GE were close together (Figure 11A). The optimal BP-NN model had three hidden layers, 10 neurons per layer, a learning rate of 0.1, a momentum term of 0.4, and 500 iterations. After data fusion, there were two unassigned samples in the calibration set (*Ac*: 97.00%); *Se* and *Sp* were 1.0 and 1.0, respectively; the error rate decreased consistently as the training iteration number increased (Figure 11C). The AUC-ROC was 1 for the four sample types (Supplementary Figure S8), indicating that appropriate parameters had been selected.

**TABLE 2:** Species identification results and model parameters.

Model	Data matrix	Calibration set					Validation set	
		Misclassified samples	Not assigned samples	Se	Sp	Ac	Misclassified or not-assigned samples	Ac
PLS-DA	EN	2	6	0.9400	1.0000	0.9200	4	0.8824
	EE	3	7	1.0000	0.9800	0.9000	5	0.8529
	ET	0	3	1.0000	1.0000	0.9700	0	1.0000
	HPLC	4	4	0.9400	0.9800	0.9200	0	1.0000
	**Data fusion by PCs**	**0**	**0**	**1.0000**	**1.0000**	**1.0000**	**1**	**0.9706**
	**Data fusion by LVs**	**0**	**0**	**1.0000**	**1.0000**	**1.0000**	**1**	**0.9706**
BP-NN	EN	1	2	0.9700	1.0000	0.9700	2	0.9412
	EE	1	1	1.0000	1.0000	0.9800	3	0.9118
	ET	2	3	0.9700	0.9800	0.9500	1	0.9706
	HPLC	4	5	0.9400	0.9700	0.9100	0	1.0000
	**Data fusion by PCs**	**0**	**2**	**1.0000**	**1.0000**	**0.9800**	**1**	**0.9706**
	Data fusion by LVs	1	2	1.0000	1.0000	0.9700	2	0.9412

The bold values indicate the optimal model.

The LVs were then fused for the electronic sensor and HPLC data. The classification results of a PLS-DA model built with the fused LV data were the same as the results of the model built with the fused PC data: no unclassified or misclassified samples in the calibration set; 100% accuracy; and only S5 unclassified in the validation set. *Se* and *Sp* were 1.00, 1.00, respectively. The four sample types were fully separated based on the first two LVs (Figure 11B), and the model performed better than it did with the fused PCs. However, the classification performance of a BP-NN model built with the fused LV data was not significantly improved compared to models built with single-source data. The optimal model had two hidden layers, 15 neurons per layer, a learning rate of 0.1, a momentum term of 0.4, and was trained for 500 iterations. In the calibration set, this model misclassified one sample and two others were unassigned (*Ac*: 97%); there was one unassigned and one misclassified sample in the validation set (*Ac*: 94.12%). *Se* and *Sp* were 1.00, 1.00, respectively. After 500 training iterations, the error rate was 0 (Figure 11D).

### 3.6 Highly contributing feature analysis

**FIGURE 12:**
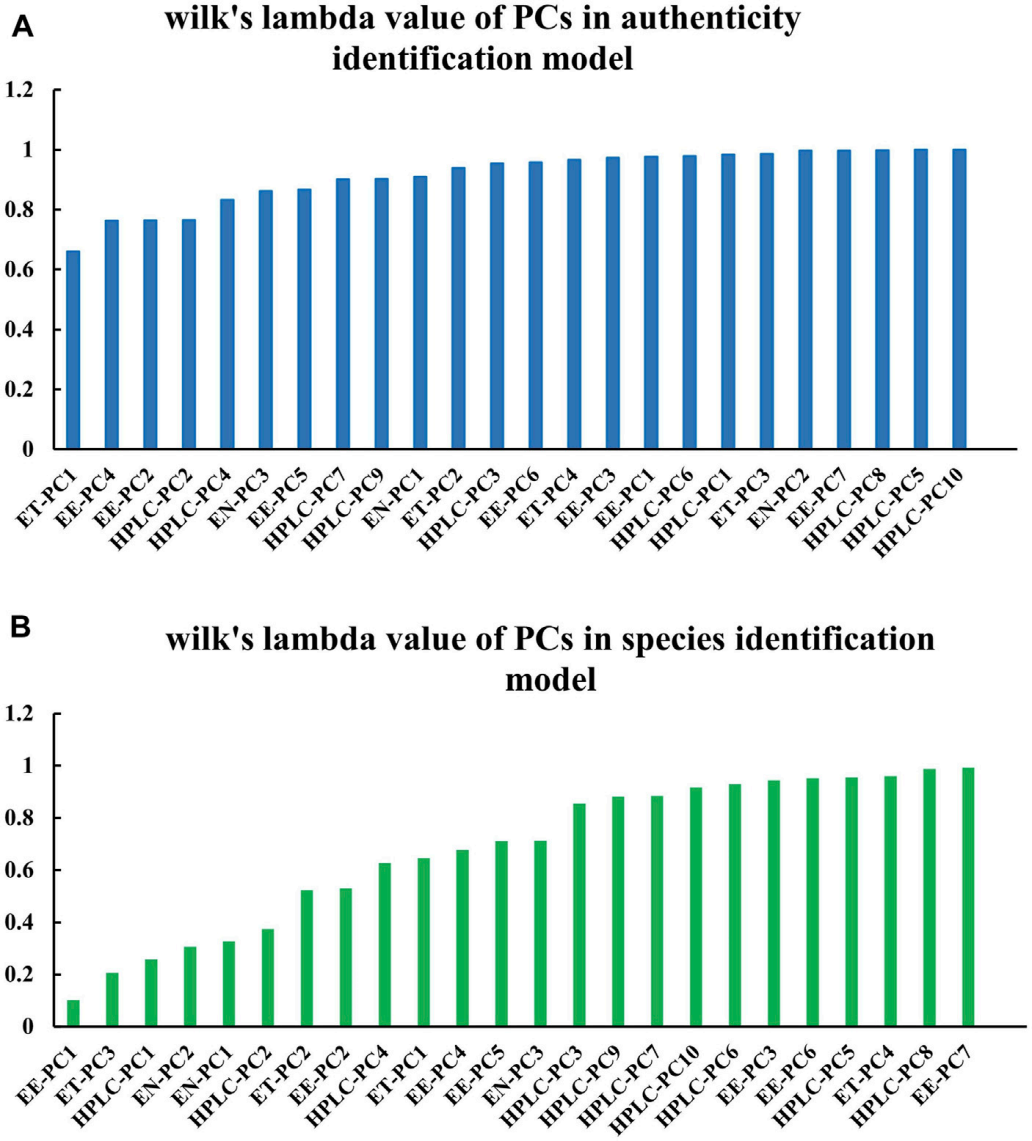
Wilk’s lambda values of variables in the PCs-based data fusion models. Data are shown for the **(A)** sample authenticity binary classification model and the **(B)** species identification multi-class model.

In classifying sample authenticity, the PCs with relatively small Wilk’s lambda values were electronic tongue (ET)-PC1, electronic eye (EE)-PC4, EE-PC2, and HPLC-PC2 (Figure 12A). In species identification, the PCs with small Wilk’s lambda values included EE-PC1, ET-PC3, HPLC-PC1, and electronic nose (EN)-PC2 (Figure 12B). These PCs contributed greatly to the classification model. Notably, the results indicated that it was not only the first three PCs that played major roles in classification with each data type; other PCs also represented a great deal of variance.

We next constructed a factor loading matrix of the four types of data sources (Supplementary Table S1). Some of the original variables (Table 3) had larger factor loading values of the highly contributing PCs. These original variables were highly correlated with the PCs, and changes in these values would be expected to have a strong impact on the classification performance of the model.

**TABLE 3:** Original variables with larger factor loading values of highly contributing principal components.

Problems of classification	PCs	Original variables
Authenticity identification	ET-PC1	Sourness
	EE-PC4	Color number value 1621
	EE-PC2	Color number value 1621
	HPLC-PC2	Peak 4
Species identification	EE-PC1	Color number value 1621
	ET-PC3	Sourness
	HPLC-PC1	Peak 5
	EN-PC2	W1C

## 4 Conclusion

In this study, a preliminary identification of *B. striata* and similar decoction pieces was firstly conducted based on the classification scheme in the *Chinese Pharmacopoeia* and local standards. Samples were then analyzed with GC-IMS, an electronic nose, an electronic eye, an electronic tongue, and HPLC. Classical machine learning and deep learning algorithms were used to classify samples based on each type of data. Furthermore, using improved data fusion technology, highly effective models were constructed to accurately distinguish between *B. striata* and similar decoction pieces. In the sample authenticity binary classification model (*B. striata* vs. other samples), the PLS-DA and SVM models built with fused LV data had the best performance, with an accuracy of 100% after cross-validation of the calibration dataset and only one misclassified sample in the validation set. In the multi-class species identification model, the PLS-DA model built with fused PCs and the PLS-DA model generated with fused LVs performed the best, with accuracies of 100% and just one misclassified sample each in the validation set. The results of feature extraction were compared between a supervised and an unsupervised algorithm; overall, fused LVs performed better than fused PCs in the PLS-DA and SVM authenticity identification models, whereas fused PCs performed comparably to or better than fused LVs fusion in the BP-NN authenticity and species identification models. PCs beyond the first three components can make large contributions to sample classification, sometimes playing key roles in model identification. Factor loading values indicated that some original variables had higher values of the highest-contributing PCs, demonstrating the importance of specific original variables in accurately classifying samples. These variables included the Sourness sensor in the ET, the W1C sensor in the EN (aromatic organic compounds), color number 1621 in the EE (dark reddish gray), and peaks 4 and 5 in the HPLC data. In summary, our study provides a highly feasible method of accurately evaluating putative *B. striata* and related samples, promoting quality evaluation and control in Chinese decoction pieces.

## Data Availability

The original contributions presented in the study are included in the article/Supplementary Material, further inquiries can be directed to the corresponding authors.
