# Supplementary material for: Identification of Bletilla striata and related decoction pieces: a data fusion method combining electronic nose, electronic tongue, electronic eye, and high-performance liquid chromatography data
Source: Front Chem. 2024 Jan 10;11:1342311. doi: 10.3389/fchem.2023.1342311 (PMC10806155; doi:10.3389/fchem.2023.1342311)
Supplement: Supplementary file 1 [file DataSheet1.docx]

Supplementary Material

# Supplementary Figures and Tables

## Supplementary Figures


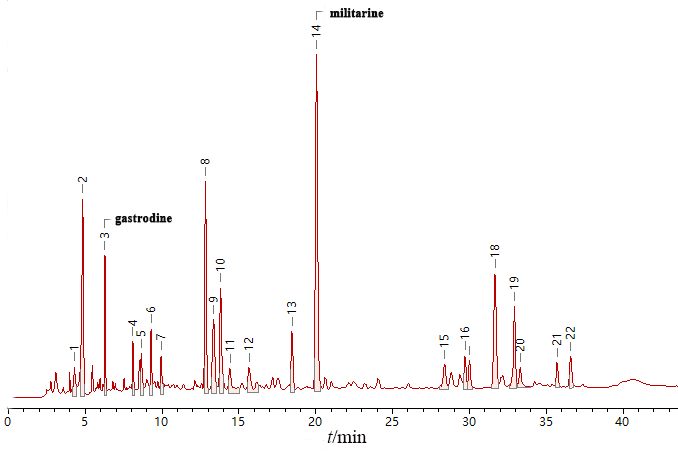


**Supplementary Figure 1.** HPLC control fingerprint of *Bletilla striata.*


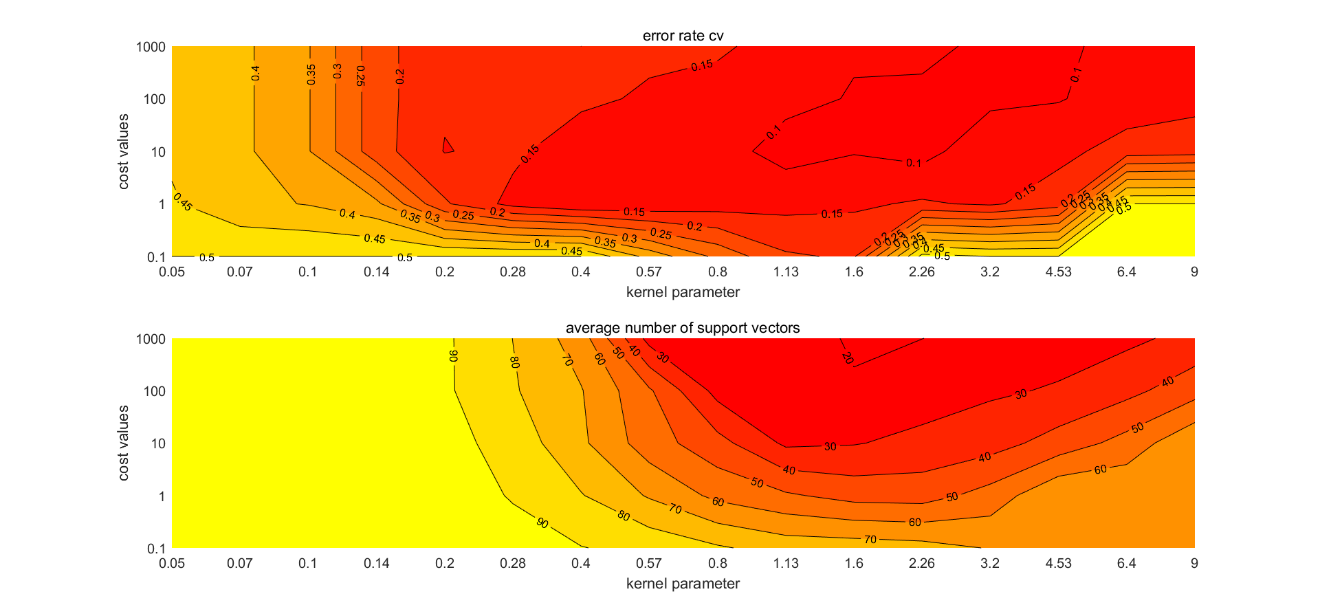


**Supplementary Figure 2.** SVM parameter optimization in SVM authenticity and counterfeit identification model based on electronic nose.


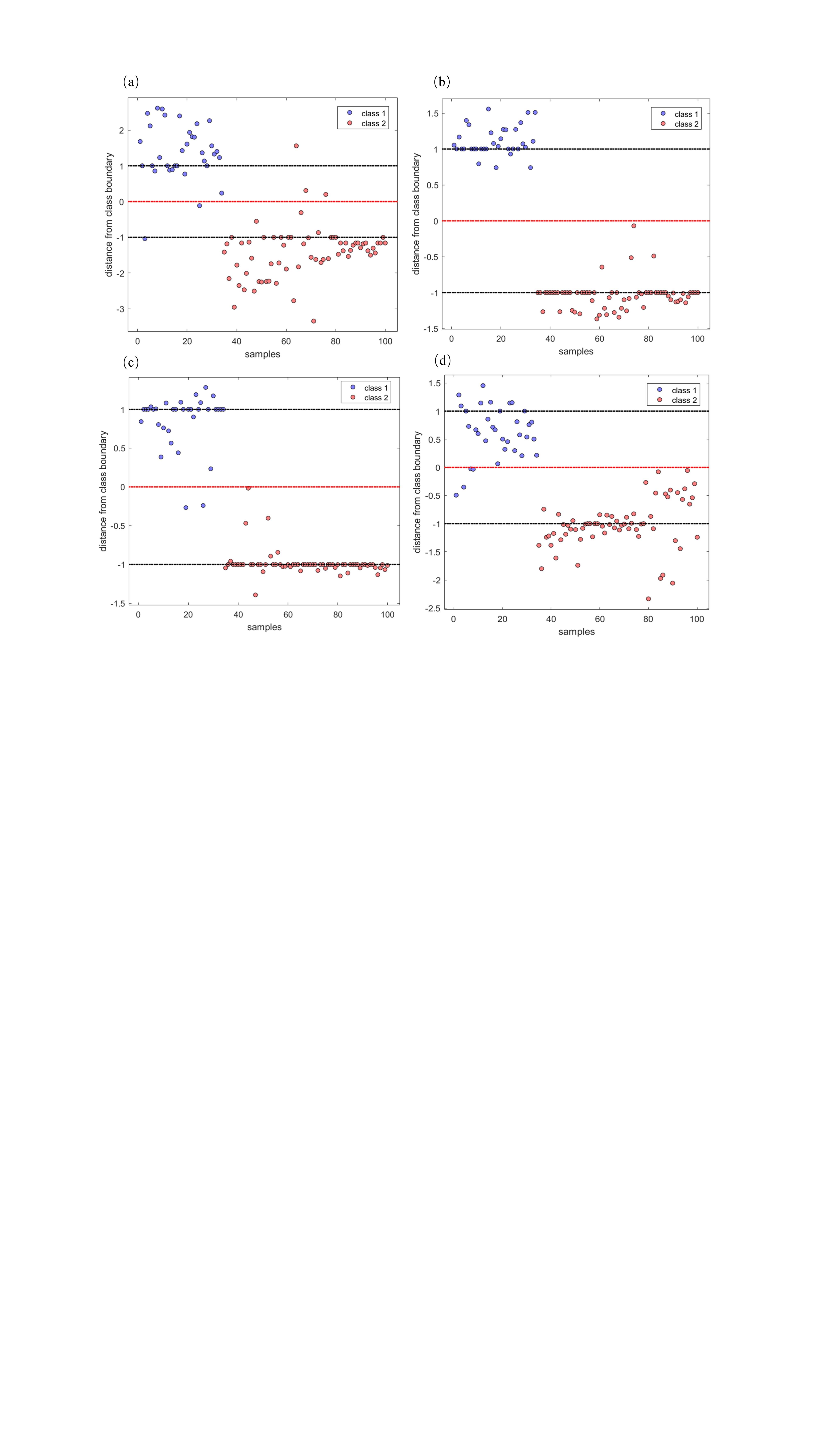


**Supplementary Figure 3.** Classification hyperplanes and support vectors of single-source SVM authenticity and counterfeit identification model based on (a) electronic nose, (b) electronic tongue, (c) electronic eye and (d) high-performance liquid chromatography. Class1, *Bletilla striata*; class2, counterfeit species (*Gastrodia elata*, *Polygonatum odoratum*, and *Bletilla ochracea schltr*).


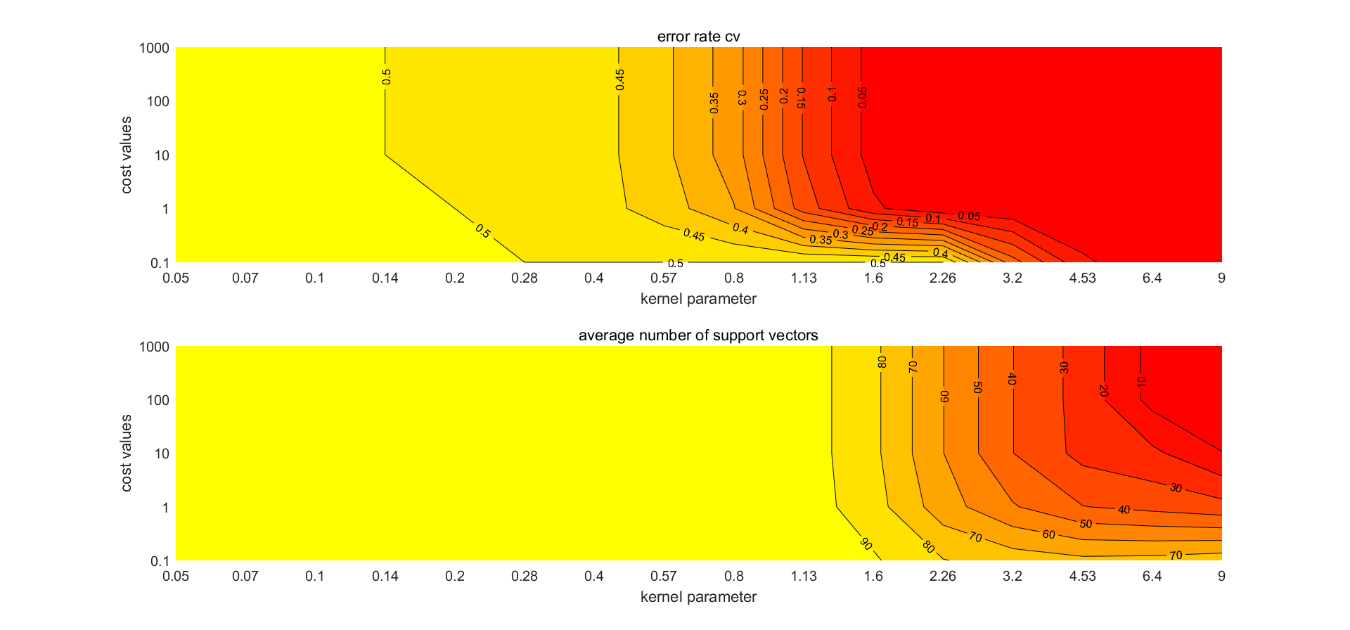


**Supplementary Figure 4.** SVM parameter optimization in SVM authenticity and counterfeit identification model based on electronic eye.


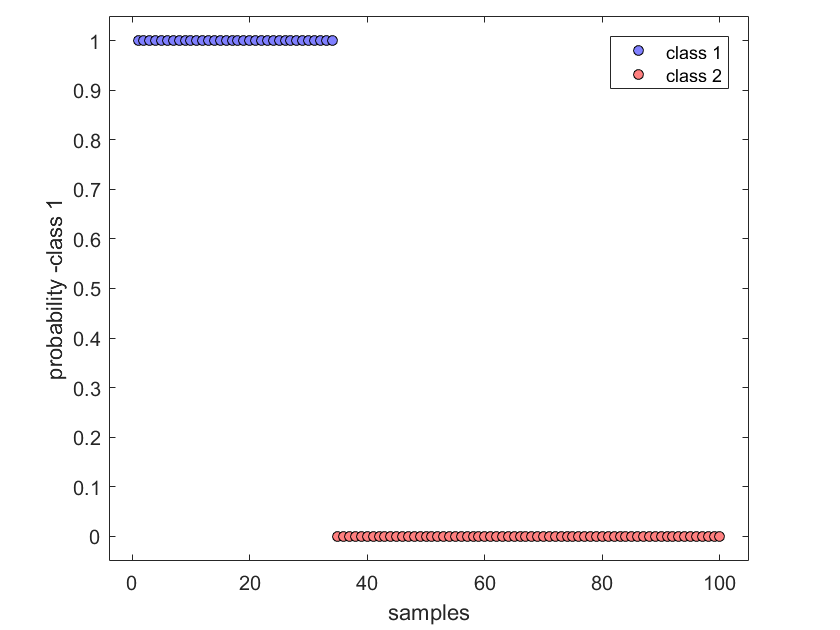


**Supplementary Figure 5.** Classification prediction probability for two sample types in SVM authenticity and counterfeit identification model based on PCs-fused data.


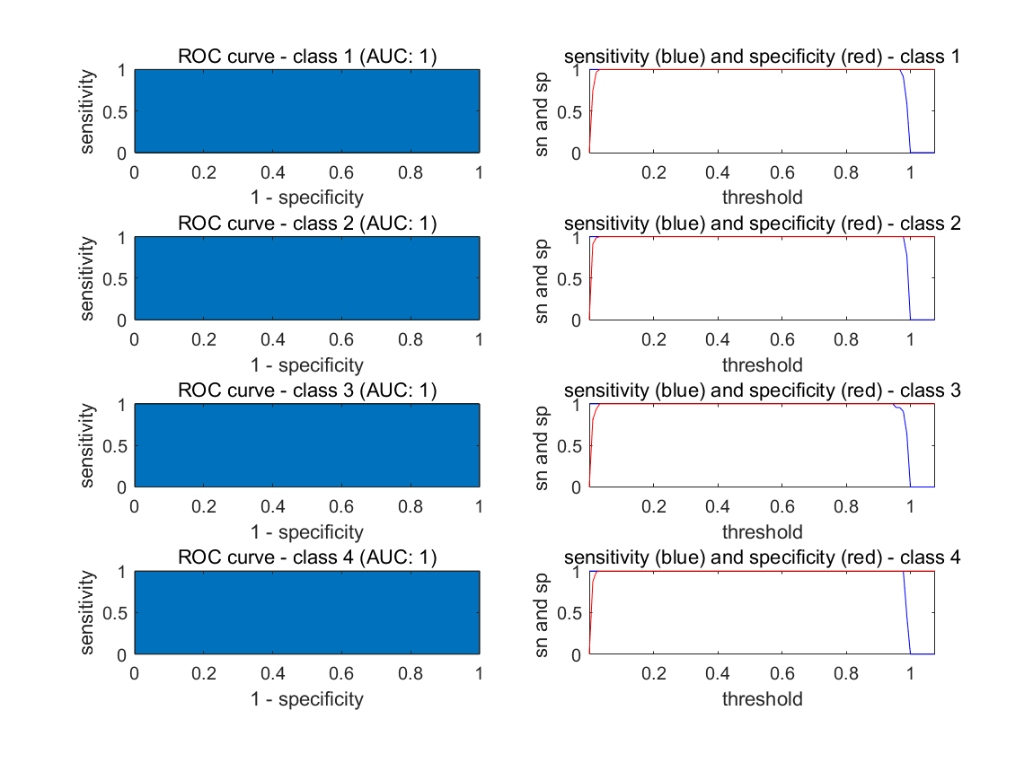


**Supplementary Figure 6.** Receiver operating characteristic (ROC) curves (left), sensitivity, and specificity (right) for a model built with electronic eye in species identification.


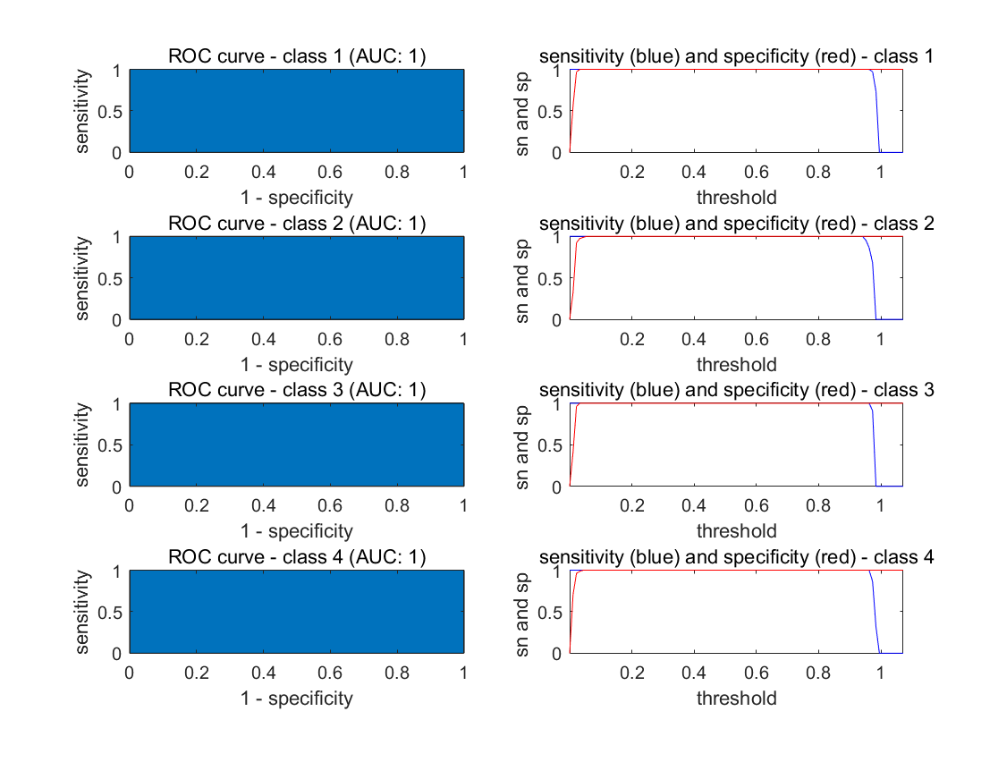


**Supplementary Figure 7.** Receiver operating characteristic (ROC) curves (left), sensitivity, and specificity (right) for a model built with electronic tongue in species identification.


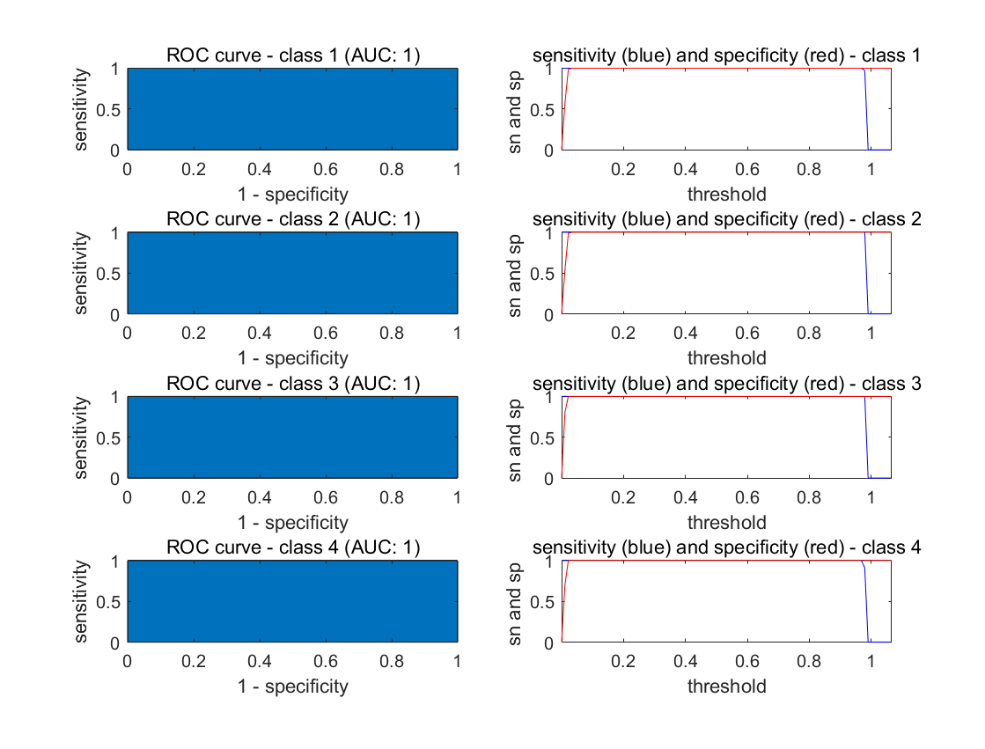


**Supplementary Figure 8.** Receiver operating characteristic (ROC) curves (left), sensitivity, and specificity (right) for a model built with PCs-fused data in species identification

## Supplementary Table1

Factor loading matrix of the four types of data sources.

**Electronic nose:**

|  | W1C | W5S | W3C | W6S | W5C | W1S | W1W | W2S | W2W | W3S |
| --- | --- | --- | --- | --- | --- | --- | --- | --- | --- | --- |
| PC1 | -0.964886955 | 0.082998485 | 0.196949624 | -0.025735536 | -0.069741316 | -0.103356516 | -0.048829038 | 0.063248484 | 0.019452468 | -0.018533153 |
| PC2 | 0.721619906 | -0.226353806 | 0.59936923 | 0.242407366 | 0.096550714 | -0.023332314 | -0.004793807 | 0.000161352 | 0.010125696 | 0.005646854 |
| PC3 | -0.96950127 | 0.071030861 | 0.208137508 | 0.006789168 | -0.043204032 | -0.044809263 | 0.068836289 | 0.023291144 | -0.042709933 | 0.025982309 |
| PC4 | 0.33589412 | 0.906416882 | -0.063863966 | 0.217339189 | -0.090650515 | 0.053110599 | -0.053674536 | 0.013454203 | -0.01297122 | 0.00041334 |
| PC5 | -0.940011072 | 0.238171545 | 0.173063629 | 0.080565234 | -0.095472651 | 0.022118047 | 0.101376701 | -0.050606315 | 0.022767653 | -0.015839396 |
| PC6 | 0.95172746 | 0.034016458 | -0.228971355 | 0.05336437 | -0.109095858 | -0.156482527 | 0.011955172 | -0.027725055 | 0.014577899 | 0.016409713 |
| PC7 | 0.917580666 | -0.037237033 | 0.349030729 | -0.142824379 | -0.101900872 | -0.023529521 | -0.02655926 | -0.032493961 | -0.035087784 | -0.022539797 |
| PC8 | 0.947502824 | -0.14600435 | -0.234447719 | 0.107618913 | 0.016367877 | -0.011889039 | 0.1028276 | 0.051459917 | -0.013629087 | -0.023599097 |
| PC9 | 0.947659495 | 0.017228977 | 0.226524316 | -0.150596642 | -0.128586954 | 0.084162551 | 0.037292181 | 0.04122551 | 0.025675753 | 0.016870005 |
| PC10 | 0.407734101 | 0.871940265 | 0.119113479 | -0.170299308 | 0.160721631 | -0.052374448 | 0.040782276 | -0.000542813 | 0.006664662 | -0.000877399 |

**Electronic tongue:**

|  | Sourness | Bitterness | Astringency | Aftertaste-B | Aftertaste-A | Umami | Richness | Saltiness |
| --- | --- | --- | --- | --- | --- | --- | --- | --- |
| PC1 | 0.75088871 | -0.586659974 | 0.255651132 | 0.098684909 | 0.037091547 | -0.077967284 | 0.095163457 | 0.019729248 |
| PC2 | 0.804819617 | 0.074610738 | -0.532696292 | -0.082251068 | -0.226431765 | 0.011551484 | 0.025076962 | -0.064299373 |
| PC3 | 0.718081529 | 0.231668119 | 0.595011995 | -0.140430595 | -0.214911431 | 0.090057382 | -0.017931477 | 0.048062257 |
| PC4 | 0.825729429 | 0.138955088 | -0.520751973 | 0.072343372 | 0.026691715 | -0.12318947 | -0.046147269 | 0.066547345 |
| PC5 | 0.893935723 | 0.292499941 | -0.141489882 | 0.09237281 | 0.239321785 | 0.171110247 | 0.014040045 | -0.004453 |
| PC6 | -0.676810658 | 0.637146658 | -0.319720203 | -0.146987847 | -0.049526298 | 0.021892721 | 0.082108694 | 0.049709665 |
| PC7 | -0.0622602 | 0.372125032 | 0.130365773 | 0.914176564 | -0.067126245 | -0.017796956 | 0.006606033 | -0.008142361 |
| PC8 | 0.379047567 | 0.771123127 | 0.416046464 | -0.242558019 | 0.092419492 | -0.139697131 | 0.007319023 | -0.040654558 |

**Electronic eye:**

Color number value 1348-2421:

|  | 1348 | 1620 | 1621 | 1637 | 1876 | 1877 | 1892 | 1893 | 1894 | 2133 | 2148 | 2149 | 2150 | 2165 | 2166 | 2167 | 2404 | 2405 | 2406 | 2420 | 2421 |
| --- | --- | --- | --- | --- | --- | --- | --- | --- | --- | --- | --- | --- | --- | --- | --- | --- | --- | --- | --- | --- | --- |
| PC1 | -0.473864339 | -0.373481899 | 0.590157641 | 0.026013331 | 0.001930181 | -0.307431618 | 0.175002001 | -0.167052814 | 0.25881878 | 0.147189144 | 0.006960184 | 0.02214201 | -0.010004269 | -0.026985815 | -0.066684929 | -0.010815054 | 0.029482256 | -0.002204797 | -0.012406133 | 0.031773583 | -0.007989613 |
| PC2 | -0.530070584 | -0.404609062 | 0.622885666 | 0.042887874 | 0.027043645 | -0.2765065 | 0.13335728 | -0.109912894 | 0.172751454 | 0.114416108 | 0.023894875 | 0.024700553 | -0.016071535 | -0.020892533 | -0.050067005 | -0.02482418 | 0.052858592 | -0.005186973 | 0.013916786 | -0.00078854 | -0.029443964 |
| PC3 | -0.529594257 | -0.401379982 | 0.588035786 | 0.044553252 | 0.007056479 | -0.286130961 | 0.152405212 | -0.153442011 | 0.209070682 | 0.109736971 | 0.006494821 | 0.048051239 | 0.022851109 | -0.018551406 | -0.047012356 | 0.01643337 | -0.015261536 | 0.007427866 | -0.045398605 | 0.030626661 | 0.037576194 |
| PC4 | -0.579950274 | -0.431284708 | 0.614416085 | 0.0567537 | 0.035176651 | -0.232688598 | 0.103201109 | -0.069471098 | 0.096015179 | 0.059073374 | 0.019501189 | 0.045965391 | 0.017032497 | -0.014579148 | -0.026967382 | 0.00720261 | -0.005293669 | 0.007043857 | -0.031767073 | 0.016068468 | 0.023433211 |
| PC5 | -0.565415441 | -0.466004435 | 0.577117047 | 0.04524291 | 0.047675411 | -0.177189556 | 0.099325846 | -0.019333214 | 0.066585936 | 0.090123212 | 0.053828261 | -0.004373919 | -0.069590613 | -0.007582424 | -0.028036014 | -0.063645088 | 0.106807418 | -0.02308465 | 0.060071798 | -0.059766844 | -0.0949687 |
| PC6 | -0.562197449 | -0.491171582 | 0.578351954 | 0.02581704 | 0.015715303 | -0.209996245 | 0.166889623 | -0.086956115 | 0.105830849 | 0.094549035 | 0.007362184 | 0.005639866 | -0.033898101 | -0.006743966 | -0.02773341 | -0.007887734 | 0.025280846 | -0.002449172 | -0.00867531 | -0.008155766 | -0.009046199 |
| PC7 | -0.629040465 | -0.448109085 | 0.578500538 | 0.081439277 | 0.066479023 | -0.136044081 | 0.053463045 | 0.023583455 | -0.018454991 | 0.039179206 | 0.035654896 | 0.00648824 | -0.037433926 | -0.005632165 | -0.010316722 | -0.036018385 | 0.056961407 | -0.022651863 | 0.037966997 | -0.038065924 | -0.068374878 |
| PC8 | -0.600889884 | -0.464538287 | 0.613059002 | 0.053682566 | 0.058986982 | -0.168371138 | 0.073426617 | 0.016642322 | -0.020010121 | 0.018459898 | 0.026966868 | 0.029057619 | -0.003160912 | -0.005128379 | -0.007788405 | -0.019389318 | 0.014595969 | -0.007261102 | -0.004952889 | -0.01289263 | 0.009846977 |
| PC9 | -0.600061627 | -0.462515416 | 0.606054193 | 0.048101093 | 0.05018805 | -0.176419087 | 0.078287771 | 0.006162246 | -0.017309752 | 0.005297653 | 0.020147839 | 0.051215076 | 0.024520279 | -0.003830109 | -0.008715157 | -0.002232388 | -0.020180581 | 0.006070418 | -0.033736896 | 0.003136745 | 0.052180241 |
| PC10 | 0.006907261 | -0.633221291 | -0.14256082 | -0.244995115 | -0.152956163 | 0.23646617 | 0.414599466 | 0.007924398 | -0.068391482 | 0.155247627 | -0.080851435 | -0.282477088 | -0.325473649 | -0.015077321 | 0.144657766 | 0.113882034 | 0.043599921 | 0.033026939 | -0.011605383 | 0.011017586 | 0.041317754 |
| PC11 | -0.126920673 | -0.824914711 | -0.145876908 | -0.230322691 | 0.037986146 | 0.249281025 | 0.257869584 | 0.170931318 | 0.113214625 | 0.04868552 | -0.013740306 | -0.090523033 | -0.177349457 | -0.001875512 | 0.03704689 | 0.017205453 | 0.006595725 | -0.006430169 | 0.010537833 | -0.011665987 | -0.005184562 |
| PC12 | -0.364604658 | -0.831376167 | 0.13737237 | -0.167144282 | 0.042079673 | 0.153540304 | 0.225676004 | 0.122133502 | -0.084506109 | -0.008060741 | -0.069681577 | -0.067110221 | -0.106210751 | -0.052257245 | 0.005538962 | 0.02386015 | -0.008531363 | -0.013558745 | 0.006806151 | -0.007346156 | 0.013967171 |
| PC13 | -0.573303853 | -0.64444499 | 0.446797687 | -0.033818301 | 0.050300223 | -0.022996629 | 0.112291839 | 0.065084827 | -0.158665465 | -0.042158307 | -0.026065154 | 0.023985192 | 0.002559697 | -0.029100835 | -0.017737528 | -0.002009574 | -0.019870459 | -0.001946266 | -0.012777003 | -0.014158691 | 0.025464157 |
| PC14 | -0.714322928 | -0.453131922 | 0.416137838 | 0.144350882 | 0.119684214 | 0.026014499 | -0.047884168 | 0.15817472 | -0.17706989 | -0.059202586 | 0.043400731 | 0.02240275 | -0.012292355 | 0.000474664 | 0.027712233 | -0.021064832 | 0.01481812 | -0.022649249 | 0.012776794 | -0.028468592 | -0.022958044 |
| PC15 | -0.641580064 | -0.437615952 | 0.518276007 | 0.093643197 | 0.103816239 | -0.021514666 | -0.050008068 | 0.176563482 | -0.235413341 | -0.088966975 | 0.048275839 | 0.035228562 | 0.002417295 | -0.00190461 | 0.039178959 | -0.0168818 | 0.006275944 | -0.000804403 | -0.003462415 | -0.027923727 | 0.028012623 |
| PC16 | -0.648147168 | -0.428055967 | 0.544383124 | 0.085126511 | 0.081260565 | -0.072371177 | -0.021870695 | 0.143022787 | -0.189253374 | -0.07546831 | 0.036206506 | 0.053686616 | 0.016621141 | -0.004572315 | 0.045470184 | 0.011695776 | -0.02270403 | 0.017618558 | -0.017428301 | -0.013325553 | 0.063362281 |
| PC17 | 0.220354846 | -0.735817267 | -0.388997975 | -0.309420552 | -0.046777074 | 0.124155988 | 0.260766976 | -0.085253278 | -0.086268842 | -0.0358063 | -0.127606104 | 0.008796994 | 0.140153161 | 0.008307826 | -0.08559509 | -0.032145883 | -0.002427045 | -0.02059661 | 3.29E-05 | 0.012014344 | -0.025024518 |
| PC18 | 0.161239987 | -0.735627598 | -0.357173609 | -0.320818656 | -0.047449168 | 0.176919586 | 0.294150349 | -0.070101519 | -0.08893425 | -0.040538083 | -0.187095576 | -0.014554986 | 0.095400451 | -0.051556335 | -0.130062068 | -0.012820994 | -0.040234002 | -0.013295174 | 0.000253078 | 0.002045397 | -0.023353904 |
| PC19 | -0.114618137 | -0.78200827 | -0.186762563 | -0.275400194 | -0.034658365 | 0.171179691 | 0.293820394 | -0.092247673 | -0.110500995 | -0.058730664 | -0.184098979 | 0.049188205 | 0.126860031 | -0.068553474 | -0.203014934 | -0.023076811 | -0.098177745 | -0.011027408 | -0.025032623 | -0.007127571 | -0.025919413 |
| PC20 | 0.173861653 | -0.776709944 | -0.4134234 | -0.219512164 | 0.035869336 | 0.164415151 | 0.161824047 | 0.128409587 | 0.168601685 | -0.000297347 | 0.058212509 | 0.046267834 | 0.017380147 | 0.160930003 | -0.006729288 | -0.040646702 | -0.022830927 | 0.037295986 | -0.025870351 | -0.020085882 | -0.056097086 |
| PC21 | -0.09583874 | -0.81411012 | -0.318945514 | -0.109795652 | 0.132308185 | 0.220375343 | 0.056622056 | 0.268785127 | 0.212695836 | -0.043525101 | 0.025887235 | 0.059798714 | -0.002350832 | 0.062038626 | -0.048834562 | -0.007351332 | -0.060638262 | 0.038568801 | -0.007409146 | -0.043091638 | -0.044901703 |

|  | 1348 | 1620 | 1621 | 1637 | 1876 | 1877 | 1892 | 1893 | 1894 | 2133 | 2148 | 2149 | 2150 | 2165 | 2166 | 2167 | 2404 | 2405 | 2406 | 2420 | 2421 |
| --- | --- | --- | --- | --- | --- | --- | --- | --- | --- | --- | --- | --- | --- | --- | --- | --- | --- | --- | --- | --- | --- |
| PC22 | -0.647153263 | -0.647408389 | 0.162751349 | 0.080170506 | 0.141661489 | 0.141924256 | -0.05072288 | 0.223545238 | -0.126149054 | -0.09533812 | 0.025513018 | 0.066747218 | 0.021139036 | -0.01611161 | -0.026761285 | 0.02006069 | -0.030625573 | 0.00161701 | -0.017474591 | -0.005065255 | 0.007558402 |
| PC23 | -0.723117842 | -0.490673584 | 0.382960344 | 0.106686083 | 0.081350351 | 0.000426745 | -0.028011652 | 0.130370755 | -0.188527512 | -0.067874051 | 0.012098407 | 0.064343796 | 0.029360767 | -0.017576514 | 0.011578828 | 0.04112589 | -0.021323026 | 0.016561588 | -0.018331116 | 0.001909211 | 0.043152777 |
| PC24 | -0.836193171 | -0.31692301 | 0.075422754 | 0.304488074 | 0.124166334 | 0.139144483 | -0.098412317 | 0.073099878 | -0.177573516 | -0.018545584 | 0.024205781 | 0.006061118 | 0.002347423 | 0.000255761 | 0.005027103 | -0.00792209 | 0.051487096 | -0.038233194 | -0.01816507 | 0.021378497 | -0.067118556 |
| PC25 | -0.802600022 | -0.360039787 | 0.286937626 | 0.21955124 | 0.093707969 | 0.052800214 | -0.075000689 | 0.121459344 | -0.213763919 | -0.063717879 | 0.030652684 | 0.040329165 | 0.021062406 | 0.010363308 | 0.040608731 | 0.041280648 | -0.000580448 | 0.02526177 | -0.026394764 | 0.026150377 | 0.00418451 |
| PC26 | 0.264822136 | -0.776085336 | -0.429847213 | -0.276074714 | -0.096330853 | 0.015864215 | 0.046925226 | -0.097602646 | -0.042999328 | 0.009276111 | 0.084627349 | 0.104657615 | 0.092456407 | -0.054738991 | 0.016794765 | -0.010691099 | 0.053055369 | -0.001539734 | 0.002464819 | -0.001352671 | 0.028848048 |
| PC27 | 0.262498413 | -0.790940592 | -0.441142596 | -0.246623386 | -0.058315299 | 0.043966473 | 0.035968909 | 0.00688798 | 0.06915096 | 0.01096518 | 0.119728788 | 0.105152375 | 0.053780921 | 0.023183306 | 0.018823046 | -0.010367926 | 0.037325274 | 0.011869082 | -0.00921444 | -0.013309246 | 0.025753027 |
| PC28 | 0.065502199 | -0.831452563 | -0.429133843 | -0.155590449 | 0.023641642 | 0.088183339 | -0.008402376 | 0.141034598 | 0.175212941 | -0.010611676 | 0.090831085 | 0.107462705 | -0.013973113 | 0.013337086 | -0.047644612 | 0.029337617 | -0.023311391 | 0.021762716 | 0.001521054 | -0.01985847 | 0.035352724 |
| PC29 | 0.159676137 | -0.669995426 | -0.482760164 | -0.070947666 | 0.211217557 | 0.017523954 | -0.162761954 | 0.242740598 | 0.349930834 | 0.021126019 | 0.091229753 | 0.098056479 | -0.022905992 | -0.074099487 | 0.038615962 | 0.029063235 | 0.017507123 | 0.029936554 | 0.031521106 | -0.010747128 | 0.012915434 |
| PC30 | -0.460024404 | -0.512907714 | -0.517878759 | 0.216990812 | 0.251244981 | 0.180431285 | -0.17544453 | 0.170937782 | 0.169003222 | 0.0310563 | 0.024348411 | 0.025608841 | -0.03754855 | -0.102519923 | -0.045003549 | 0.00753881 | 0.012598381 | -0.039404517 | 0.013548737 | -0.005374068 | -0.019149183 |
| PC31 | -0.834902953 | -0.348892342 | -0.1928515 | 0.281935814 | 0.097081381 | 0.160624637 | -0.064268524 | 0.059394311 | -0.063716262 | 0.001124249 | 0.013794347 | 0.03984821 | -0.001891015 | -0.011172384 | -0.005264622 | 0.05310278 | 0.011836127 | 0.011539945 | -0.009858407 | 0.01175829 | -0.026442206 |
| PC32 | -0.686977845 | 0.0065431 | -0.492467218 | 0.386245396 | 0.115749357 | 0.201061786 | -0.060010021 | -0.085827293 | -0.044815945 | 0.108592821 | -0.017800822 | -0.033715153 | -0.023835482 | -0.008341827 | -0.052232572 | -0.092093937 | 0.078041639 | -0.123286741 | -0.007681672 | 0.015381046 | -0.068795517 |
| PC33 | -0.806459594 | -0.041588257 | -0.341779642 | 0.41186612 | 0.060180812 | 0.157662474 | -0.047364367 | -0.080929835 | -0.044348865 | 0.053341761 | -0.002201006 | -0.006534701 | -0.004130685 | 0.013499843 | -0.021356249 | 0.006432314 | 0.052046695 | -0.018430142 | -0.007900628 | 0.013804196 | -0.081152189 |
| PC34 | -0.875495894 | -0.120314373 | -0.163527144 | 0.345484104 | 0.009653859 | 0.071933956 | -0.003392268 | -0.054101789 | -0.030738158 | 0.021111866 | -0.007685083 | 0.045922317 | 0.028219965 | 0.034502938 | 0.017871461 | 0.135486774 | -0.028261186 | 0.115425155 | -0.016462913 | 0.078928499 | -0.036304288 |
| PC35 | 0.264406644 | -0.752262375 | -0.406247184 | -0.276340611 | -0.14722672 | -0.018562332 | 0.10306484 | -0.192417124 | -0.177100521 | 0.019093805 | 0.01884545 | 0.045373044 | 0.020652253 | -0.052780243 | 0.061066384 | 0.010002586 | 0.079604306 | -0.008683299 | 0.008220372 | 0.019852644 | 0.021206219 |
| PC36 | 0.249090349 | -0.725724022 | -0.388580932 | -0.275363133 | -0.176768267 | -0.004050173 | 0.09779636 | -0.208902154 | -0.184929077 | 0.027595903 | 0.123820405 | 0.095739357 | 0.059254203 | 0.038210147 | 0.062459223 | -0.028887245 | 0.100029915 | -0.032660094 | -0.039067114 | 0.039833323 | 0.061826329 |
| PC37 | 0.343361669 | -0.772163365 | -0.42179522 | -0.155934528 | -0.091156714 | -0.135878768 | -0.007593808 | -0.1043422 | -0.077196308 | -0.009370417 | 0.072636516 | 0.074088108 | -0.00987938 | 0.089548292 | 0.057590342 | -0.018674458 | 0.030240943 | 0.013202778 | -0.004406568 | -0.004819663 | -0.052145038 |
| PC38 | 0.356427392 | -0.766725117 | -0.430494233 | -0.134457169 | -0.049405026 | -0.146756327 | -0.066387257 | -0.054066147 | -0.012346549 | 0.004667265 | 0.126473789 | 0.100961513 | 0.008416378 | 0.080121552 | 0.079588025 | -0.010379794 | 0.066696987 | -0.002092034 | -0.020027459 | 0.020172663 | 0.011590684 |
| PC39 | 0.325059743 | -0.735676052 | -0.490160451 | -0.066069727 | 0.118887991 | -0.137332693 | -0.146936949 | 0.075581509 | 0.131452587 | 0.004742274 | 0.083983223 | 0.095104585 | 0.013554939 | -0.014963286 | 0.024024051 | 0.006690984 | 0.013552393 | 0.00818094 | -0.01415916 | 0.002139885 | 0.030200212 |
| PC40 | 0.410625761 | -0.607093138 | -0.393056246 | -0.03825534 | 0.201639739 | -0.24926802 | -0.145013792 | 0.009822287 | -0.012124979 | 0.028221033 | -0.174447744 | -0.00445155 | 0.054856581 | -0.349681031 | 0.03380369 | 0.079819944 | 0.04959361 | 0.015728262 | 0.064186576 | 0.032527014 | 0.04573958 |
| PC41 | 0.117036773 | -0.357290064 | -0.661045358 | 0.272804758 | 0.468338999 | -0.134600677 | -0.127131815 | 0.073589276 | 0.072366159 | 0.135356721 | -0.039871874 | -0.079446972 | -0.015240181 | -0.135636437 | 0.000372101 | -0.060161561 | -0.004699912 | -0.06697233 | -0.079055255 | 0.058453111 | 0.026959075 |
| PC42 | -0.608720384 | 0.003510547 | -0.608986978 | 0.402961416 | 0.149661163 | 0.138935406 | -0.081045601 | -0.10449822 | -0.024736985 | 0.087163479 | 0.025749972 | -0.035755607 | -0.004535252 | -0.018712942 | -0.04973904 | -0.026783335 | 0.071722497 | -0.032005011 | -0.05093428 | -0.022968699 | 0.009229696 |

|  | 1348 | 1620 | 1621 | 1637 | 1876 | 1877 | 1892 | 1893 | 1894 | 2133 | 2148 | 2149 | 2150 | 2165 | 2166 | 2167 | 2404 | 2405 | 2406 | 2420 | 2421 |
| --- | --- | --- | --- | --- | --- | --- | --- | --- | --- | --- | --- | --- | --- | --- | --- | --- | --- | --- | --- | --- | --- |
| PC43 | -0.813468955 | -0.042702986 | -0.432877803 | 0.336987692 | 0.005265296 | 0.071058656 | -0.014912506 | -0.084774749 | 0.026683067 | 0.048227751 | 0.008529895 | 0.046534559 | 0.021231208 | 0.016073771 | -0.014588723 | 0.07296698 | 0.014933953 | 0.038431869 | -0.024306013 | 0.01642119 | -0.008901838 |
| PC44 | -0.649092677 | 0.192868059 | -0.570200972 | 0.36210943 | 0.053580586 | 0.128270994 | -0.012856134 | -0.106492061 | -0.060542787 | 0.107987856 | -0.008793449 | -0.007726606 | -0.029049868 | 0.001377793 | -0.053986295 | -0.060917103 | 0.063318893 | -0.0376345 | -0.014409905 | 0.011373518 | 0.033094241 |
| PC45 | -0.771377444 | 0.113788531 | -0.444263888 | 0.38331769 | -0.026816239 | 0.078474778 | 0.01996478 | -0.132020032 | 0.006524429 | 0.062245844 | -0.019141994 | 0.018865289 | 0.008466946 | 0.021971671 | -0.009443933 | 0.067861922 | 0.010079081 | 0.029807455 | -0.028033021 | 0.027370405 | -0.021794672 |
| PC46 | -0.829330679 | 0.045440619 | -0.328200762 | 0.349959967 | -0.058368912 | 0.01587456 | 0.056133377 | -0.117729601 | 0.041015163 | 0.040516163 | -0.029770056 | 0.058071305 | 0.030679751 | 0.043606365 | 0.037608684 | 0.133978819 | -0.060850291 | 0.09905713 | -0.001626157 | 0.071414303 | -0.017578031 |
| PC47 | 0.40835599 | -0.679244068 | -0.326233163 | 0.021979148 | -0.230106939 | -0.237224063 | -0.154207405 | -0.155564962 | -0.159151477 | 0.007687579 | 0.12338216 | 0.04457399 | -0.138478249 | -0.029120718 | 0.02767261 | -0.030389062 | -0.103709746 | 0.039704025 | -0.006121435 | -0.036505656 | -0.116302678 |
| PC48 | 0.426399092 | -0.705109373 | -0.343117746 | 0.028087488 | -0.180834665 | -0.255166784 | -0.081381678 | -0.133490713 | -0.166335141 | -0.046716965 | 0.048548 | 0.045453887 | -0.11549752 | 0.133026294 | -0.021501759 | 0.002464904 | -0.012580787 | -0.024429726 | -0.005545133 | 0.022477347 | -0.022515234 |
| PC49 | 0.498556637 | -0.624929818 | -0.263733588 | 0.203216675 | -0.242812293 | -0.303811815 | -0.194186093 | -0.046081635 | -0.138114065 | 0.027156828 | -0.006076972 | -0.0262995 | -0.04582164 | -0.091667639 | -0.040677143 | 0.049945468 | -0.046889711 | 0.019936036 | 0.028638516 | -0.072485512 | -0.046420092 |
| PC50 | 0.532826055 | -0.608152573 | -0.26322897 | 0.235572199 | -0.116174519 | -0.34269734 | -0.17620863 | -0.002235182 | -0.094461388 | -0.037586984 | -0.132251928 | -0.03736379 | -0.033601973 | -0.063126705 | -0.061257259 | 0.086745161 | 0.006713074 | 0.000543019 | 0.050347277 | -0.04424161 | -0.00606802 |
| PC51 | 0.65535696 | -0.511201178 | -0.223678664 | 0.299349701 | 0.122749573 | -0.320378239 | -0.077300433 | 0.049585478 | -0.009252752 | -0.083787452 | -0.09343818 | -0.036232999 | -0.08431595 | 0.063639319 | -0.038119532 | 0.058357168 | -0.024016169 | -0.013947048 | 0.00013469 | 0.009727704 | -0.027935804 |
| PC52 | 0.603604854 | -0.327407201 | -0.373659321 | 0.310239122 | 0.372289678 | -0.281812244 | 0.020111324 | -0.024852513 | -0.06487131 | -0.048445832 | 0.038282178 | -0.046037004 | -0.095173509 | 0.11278393 | -0.056647452 | 0.03480218 | -0.010070494 | -0.038191657 | -0.102512673 | -0.000580427 | 0.061707217 |
| PC53 | 0.683766812 | -0.345991922 | -0.071126866 | 0.402093708 | 0.064163839 | -0.258722863 | 0.019481924 | 0.153934537 | -0.031432848 | 0.067101961 | -0.261747206 | -0.153539529 | 0.121930317 | 0.054409743 | 0.074351759 | 0.007552761 | 0.05622068 | 0.072418112 | -0.046105036 | -0.062320541 | -0.043818854 |
| PC54 | 0.737881619 | -0.182509263 | -0.092801773 | 0.306572624 | 0.423685404 | -0.187719169 | 0.162507057 | 0.079247969 | -0.007758962 | 0.008411188 | -0.131354441 | -0.018272015 | 0.023604197 | 0.073123107 | 0.112616523 | -0.050123254 | -0.031567041 | 0.011347864 | 0.002000203 | 0.068062486 | -0.076157028 |
| PC55 | 0.233957527 | 0.247967311 | -0.49306691 | 0.358562311 | 0.60219984 | -0.109156065 | 0.226681097 | -0.054389631 | -0.108197312 | 0.082764887 | 0.048891668 | -0.021693206 | -0.050088187 | 0.017960186 | -0.005278252 | -0.170891938 | -0.052641959 | 0.014489452 | -0.009727621 | -0.028283931 | 0.070798069 |
| PC56 | -0.589791873 | 0.3022822 | -0.621718661 | 0.341139417 | 0.061734869 | -0.00388412 | 0.075056623 | -0.095212156 | -0.055540954 | 0.037895005 | 0.046546846 | -0.036953375 | -0.016839402 | 0.003123599 | -0.070633328 | -0.026246443 | 0.044677874 | 0.071409061 | 0.020556683 | -0.076240029 | 0.056699211 |
| PC57 | -0.780845602 | 0.18667223 | -0.472437134 | 0.272162519 | -0.107541373 | -0.077313269 | 0.097467101 | -0.084919137 | 0.056127212 | 0.012996202 | -0.012583874 | 0.032832462 | 0.018808231 | 0.01754699 | -0.00315494 | 0.066328352 | -0.015431938 | 0.055248573 | 0.004452366 | -0.006752659 | 0.024821145 |
| PC58 | -0.606418341 | 0.412611486 | -0.592611857 | 0.198537083 | -0.048007841 | -0.030183016 | 0.102393447 | 0.007123236 | -0.067777207 | 0.059866366 | 0.002597027 | 0.052640416 | -0.047164828 | 0.012278079 | -0.039270026 | -0.051135395 | 0.024993959 | 0.034216616 | 0.094075689 | -0.076921268 | 0.056902875 |
| PC59 | -0.746622626 | 0.265706226 | -0.479662876 | 0.258842335 | -0.144776924 | -0.081616203 | 0.134749574 | -0.048064699 | 0.050833056 | -0.013101269 | -0.026154213 | 0.047231077 | 0.002212944 | 0.001526281 | 0.045242268 | 0.019420485 | -0.044833626 | 0.006544501 | 0.052459434 | -0.04380289 | 0.010863561 |
| PC60 | -0.795971252 | 0.159400565 | -0.378602926 | 0.281430988 | -0.139470949 | -0.10576043 | 0.148568106 | -0.084401887 | 0.098773357 | -0.005282055 | -0.045259805 | 0.059958286 | 0.024016285 | 0.008984234 | 0.095654751 | 0.062376548 | -0.103154537 | -0.007836048 | 0.028612923 | 0.007385188 | 0.004672311 |
| PC61 | 0.45182952 | -0.306136418 | 0.017620871 | 0.369108856 | -0.49082827 | 0.08825717 | 0.110493785 | 0.194066965 | -0.058286962 | 0.35006184 | 0.287214851 | -0.153975755 | 0.143893544 | -0.041859601 | 0.059304224 | 0.026812066 | -0.025181206 | -0.036953099 | 0.002965574 | 0.000560062 | 0.013476889 |
| PC62 | 0.57238342 | -0.262548978 | 0.072643141 | 0.556406608 | -0.396875696 | -0.027546075 | 0.055634059 | 0.209584194 | -0.022009797 | 0.160576416 | 0.040986838 | -0.09710559 | 0.157639436 | 0.055685929 | -0.047544551 | 0.042244354 | 0.053140398 | -0.035750673 | 0.01075845 | -0.02629871 | 0.032110237 |
| PC63 | 0.668347439 | -0.211987072 | 0.110021692 | 0.593135632 | -0.194124471 | -0.030911316 | 0.10856995 | 0.167216992 | 0.028768827 | -0.030190036 | -0.078531018 | -0.02956605 | 0.087328212 | 0.120488735 | -0.087372165 | 0.064895141 | 0.091841313 | -0.052992173 | 0.03502711 | 0.008156139 | 0.04401857 |

|  | 1348 | 1620 | 1621 | 1637 | 1876 | 1877 | 1892 | 1893 | 1894 | 2133 | 2148 | 2149 | 2150 | 2165 | 2166 | 2167 | 2404 | 2405 | 2406 | 2420 | 2421 |
| --- | --- | --- | --- | --- | --- | --- | --- | --- | --- | --- | --- | --- | --- | --- | --- | --- | --- | --- | --- | --- | --- |
| PC64 | 0.787138574 | -0.115925644 | 0.113243856 | 0.462219804 | 0.17714365 | 0.005756977 | 0.186843665 | 0.061660447 | 0.073905431 | -0.154196971 | 0.009790922 | 0.033419991 | 0.03657443 | 0.079444178 | -0.064528358 | 0.085101163 | 0.022953094 | -0.07121093 | 0.056164796 | 0.028796424 | -0.009179408 |
| PC65 | 0.772420434 | 0.124729265 | -0.036978963 | 0.294597079 | 0.403689691 | -0.131077834 | 0.177991415 | -0.029609432 | -0.058322274 | -0.091186994 | 0.15967406 | -0.064751616 | -0.048681845 | 0.044952513 | -0.092800164 | 0.053080015 | -0.078513368 | 0.029377316 | -0.036601447 | -0.031098477 | 0.013306569 |
| PC66 | 0.730748885 | -0.094174259 | 0.182245783 | 0.546868071 | -0.144667337 | 0.033072346 | 0.099057656 | 0.123399481 | 0.010966022 | 0.089385686 | -0.085377711 | 0.077446119 | 0.058094209 | 0.014932436 | 0.071933283 | -0.050072333 | 0.082260531 | 0.047465438 | -0.026982428 | -0.002548118 | 0.010767657 |
| PC67 | 0.808413778 | 0.115121902 | 0.17939965 | 0.354106724 | 0.230656337 | 0.038777183 | 0.24152362 | 0.033742945 | -0.029670927 | 0.053335826 | -0.018771704 | 0.102735778 | 0.095869047 | -0.013937803 | 0.127107704 | -0.003206397 | 0.022058146 | 0.013302881 | 0.025896521 | 0.019713674 | -0.056937314 |
| PC68 | 0.509139407 | 0.597860254 | -0.105309137 | 0.055746674 | 0.389370048 | -0.071299077 | 0.341972879 | 0.032852107 | -0.133948919 | 0.060867101 | 0.142790183 | 0.083006882 | 0.048176962 | -0.063880099 | 0.007027082 | -0.018060235 | -0.029487977 | 0.079213007 | 0.084058414 | -0.015083053 | -0.008901193 |
| PC69 | -0.356624971 | 0.645790631 | -0.522203657 | -0.006817864 | -0.072337158 | -0.207106504 | 0.215508803 | 0.133712819 | -0.057336714 | -0.099209174 | 0.09276994 | 0.005702118 | -0.014147484 | -0.037456069 | -0.060903158 | -0.02241313 | 0.047269083 | 0.073951559 | 0.087568632 | -0.082838807 | -0.004155815 |
| PC70 | -0.628146947 | 0.41548275 | -0.475987574 | 0.07728523 | -0.225885231 | -0.236492793 | 0.187837233 | 0.072704253 | 0.078910164 | -0.124858782 | 0.012712259 | -0.000491025 | 0.020582205 | -0.033407221 | 0.025705806 | 0.020222366 | -0.017586332 | -0.024356245 | -0.04707735 | -0.048294059 | -0.016670225 |
| PC71 | 0.183851464 | 0.667620339 | -0.144233823 | -0.29937178 | 0.176016378 | -0.136621946 | 0.116983804 | 0.191178825 | -0.1725928 | 0.353781888 | -0.116294108 | 0.282774257 | -0.074149358 | 0.03354398 | 0.063918754 | -0.001683243 | -0.070018691 | -0.06898334 | 0.054820255 | 0.108394507 | -0.003939112 |
| PC72 | -0.319995007 | 0.662289539 | -0.436103843 | -0.215986464 | -0.155854362 | -0.212161999 | 0.119421671 | 0.24866586 | -0.089833703 | 0.031205932 | -0.034323163 | 0.126179494 | -0.070336649 | 0.00336051 | -0.054579602 | -0.024057277 | 0.067438915 | -0.001509078 | 0.084747899 | 0.050579348 | -0.01370582 |
| PC73 | -0.544711673 | 0.482498787 | -0.479229754 | -0.028093937 | -0.273393353 | -0.218393872 | 0.170252482 | 0.174940207 | 0.020889941 | -0.144073057 | -0.02051592 | 0.054851199 | -0.013851548 | -0.039093564 | 0.066311902 | -0.023996039 | -0.004487563 | -0.091286785 | 0.003176018 | -0.018361539 | -0.039121943 |
| PC74 | -0.657052689 | 0.325729015 | -0.429834743 | 0.121801434 | -0.226879035 | -0.201155908 | 0.19286786 | 0.045660632 | 0.088249348 | -0.112507715 | -0.042607014 | 0.057774989 | 0.014940923 | -0.03871378 | 0.139633035 | 0.00387183 | -0.112336255 | -0.175587918 | -0.115106476 | -0.078125555 | 0.036955941 |
| PC75 | 0.532777541 | -0.159382119 | 0.158524419 | 0.522815941 | -0.469188069 | 0.089451768 | -0.002401645 | 0.114237553 | -0.037523617 | 0.179985856 | 0.10036865 | -0.018067701 | -0.034659118 | -0.150813453 | -0.057163915 | -0.141067601 | -0.180269051 | 0.059897937 | -0.062042128 | 0.036644126 | -0.042158522 |
| PC76 | 0.581953179 | -0.141001222 | 0.182698363 | 0.584806934 | -0.403938832 | 0.07866694 | 0.006006375 | 0.073255469 | 0.00840346 | -0.026841471 | 0.018340087 | 0.11266006 | -0.149368487 | -0.031792525 | -0.139474431 | -0.066945605 | -0.05086095 | 0.002592734 | 0.013571579 | 0.087390607 | 0.048678993 |
| PC77 | 0.657198544 | -0.071378127 | 0.230645708 | 0.563747634 | -0.230049463 | 0.121033719 | 0.065494202 | -0.033602243 | 0.039701462 | -0.191572665 | -0.006676395 | 0.143148512 | -0.183747301 | -0.016042784 | -0.087092381 | 0.03141632 | 0.03753501 | -0.057663027 | 0.043080944 | 0.053177266 | 0.03365826 |
| PC78 | 0.737961543 | 0.105390239 | 0.288813848 | 0.379700211 | 0.075498514 | 0.208300196 | 0.176073085 | -0.143671701 | 0.03078573 | -0.169351421 | 0.145511793 | 0.064548056 | -0.023890074 | -0.105924846 | 0.034860337 | 0.113768945 | 0.028227466 | -0.098748111 | 0.066995817 | 0.005744295 | -0.037234279 |
| PC79 | 0.749911885 | 0.320382373 | 0.184387312 | 0.125857697 | 0.331472829 | 0.103343197 | 0.18827031 | -0.143369527 | -0.038463662 | -0.079974387 | 0.223549044 | -0.053291239 | 0.036187344 | -0.093769833 | -0.011056068 | 0.079401237 | -0.026667493 | -0.014806758 | -0.048815528 | -0.007671127 | -0.025119763 |
| PC80 | 0.649458791 | 0.040427147 | 0.290159352 | 0.468363619 | -0.198996451 | 0.201413961 | 0.020626876 | -0.045227659 | 0.065683574 | -0.106860109 | -0.214600502 | 0.2092947 | -0.101259876 | -0.033824721 | 0.058952322 | -0.124712941 | 0.090678093 | 0.122169522 | -0.110534298 | -0.052796271 | 0.033083713 |
| PC81 | 0.740774794 | 0.272957324 | 0.335824377 | 0.260772736 | 0.010675353 | 0.247948966 | 0.080984444 | -0.098413389 | 0.00972853 | 0.013120127 | -0.05395275 | 0.224280945 | -0.039611888 | -0.105059745 | 0.091185562 | 0.046535337 | 0.055155941 | -0.000103669 | -0.077496887 | -0.103542091 | -0.044663269 |
| PC82 | 0.557591159 | 0.687816832 | 0.159962467 | -0.222808791 | 0.144897525 | 0.067893905 | 0.078185287 | 0.015242139 | -0.07587219 | 0.141754395 | 0.10085787 | 0.099962495 | -0.017977081 | -0.088849909 | -0.079473045 | 0.119851286 | 0.045739947 | 0.014910957 | -0.088578567 | -0.053329283 | -0.035841387 |
| PC83 | 0.009078107 | 0.764185527 | -0.218330551 | -0.398117497 | -0.07500737 | -0.206483325 | 0.107450813 | 0.214193275 | -0.049161455 | -0.091338803 | 0.100958497 | -0.0473954 | -0.027881997 | -0.059566986 | -0.125118621 | 0.015546582 | 0.138837703 | 0.105813989 | -0.089006959 | 0.089048605 | -0.025642396 |
| PC84 | -0.288212014 | 0.662064428 | -0.38370379 | -0.212280796 | -0.199372184 | -0.255326383 | 0.163072542 | 0.226782807 | -0.009955268 | -0.174741678 | 0.055646982 | -0.039758676 | -0.00258235 | -0.070666831 | -0.019744146 | 0.001965369 | 0.081388707 | -0.014728375 | -0.155479797 | 0.072513519 | -0.059852525 |
| PC85 | 0.318666628 | 0.606425637 | 0.091083771 | -0.428150431 | 0.045897962 | 0.00130546 | -0.133785451 | 0.10365543 | -0.100161959 | 0.386992807 | -0.109010301 | 0.174637141 | -0.064115153 | 0.095777272 | -0.142640425 | 0.16374239 | -0.009056365 | -0.056773877 | -0.078227601 | -0.105259638 | 0.001946112 |

Color number value 2422-2967:

|  | 2422 | 2423 | 2438 | 2439 | 2676 | 2677 | 2678 | 2693 | 2694 | 2695 | 2710 | 2711 | 2712 | 2932 | 2933 | 2948 | 2949 | 2950 | 2965 | 2966 | 2967 |
| --- | --- | --- | --- | --- | --- | --- | --- | --- | --- | --- | --- | --- | --- | --- | --- | --- | --- | --- | --- | --- | --- |
| PC1 | -0.009312429 | -0.039968201 | 0.03372342 | -0.05239037 | -0.02988887 | 0.028014715 | 0.0687387 | -0.109656852 | -0.043346322 | 0.08051072 | -0.051932059 | 0.014512846 | 0.005132278 | 0.022772854 | -0.029131668 | 0.003176391 | -0.000838185 | 0.012479919 | 0.008177634 | -0.011582672 | -0.003680522 |
| PC2 | 0.024713201 | 0.012701843 | -0.007581146 | 0.010260831 | -0.016106294 | -0.005741979 | -0.003271813 | 0.014092113 | 0.008522993 | -0.034791016 | 0.000937055 | 0.007542925 | 0.021412685 | -0.019828146 | -0.028318299 | -0.024433843 | -0.001692701 | 0.005310831 | 0.015254973 | -0.006511189 | -0.009380772 |
| PC3 | -0.067467141 | -0.050468164 | 0.010186981 | -0.051221648 | 0.028485887 | 0.020102005 | -0.030882031 | 0.026750697 | 0.020317841 | -0.026633411 | 0.020664719 | -0.006451715 | 0.015403163 | -0.004548747 | 0.024380778 | 0.000960488 | 0.0040111 | -0.017156933 | -0.005997683 | -0.001801176 | -0.007115337 |
| PC4 | -0.0431598 | -0.02951298 | 0.006975057 | -0.031494657 | 0.016009981 | 0.012668622 | -0.017699083 | 0.009295516 | 0.00305371 | -0.009459031 | 0.015192532 | -0.003196416 | 0.009405349 | -0.005108566 | 0.024466683 | -0.004260274 | 0.010250941 | -0.01502279 | -0.016349808 | 0.009796801 | 0.004341107 |
| PC5 | 0.105416568 | 0.081341587 | -0.016444298 | 0.074951914 | -0.033707273 | -0.026084271 | -0.005774712 | 0.017226587 | -0.003880866 | -0.039247533 | 0.008943564 | -0.001818429 | 0.005420215 | -0.025868898 | -0.021650544 | -0.034443711 | -0.00990949 | 0.018939436 | 0.017262406 | -0.001964027 | 9.76E-06 |
| PC6 | -0.005132812 | -0.002537526 | -0.006819749 | 0.012304123 | 0.010896682 | -0.005389706 | -0.028094545 | 0.040172848 | 0.008423019 | -0.026654291 | 0.013640661 | -0.004976378 | -0.007115092 | -0.003367906 | 0.012357258 | 0.004250327 | 0.001060159 | 0.001006151 | -0.002318384 | 0.005641094 | 0.01209534 |
| PC7 | 0.057746872 | 0.059792708 | 0.003106437 | 0.029785042 | -0.013524011 | -0.016732656 | 0.019693599 | -0.023230119 | -0.011079803 | 0.04679172 | 0.004056961 | -0.001958491 | -0.038553278 | 0.021804893 | 0.036945491 | 0.031938188 | 0.01540305 | -0.025625414 | -0.02690823 | 0.009189912 | 0.01686277 |
| PC8 | -0.003844388 | -0.008461871 | -0.010570284 | 0.007927271 | 0.000926391 | -0.009308065 | -0.009462955 | 0.026870241 | 0.010342159 | 0.002076801 | -0.008057288 | 0.004503201 | -0.017153499 | 0.013255754 | 0.013554439 | 0.026575014 | 0.002272767 | -0.006455357 | -0.007906998 | 0.007221562 | 0.012928404 |
| PC9 | -0.04155235 | -0.049836952 | -0.011124834 | -0.013945213 | 0.012298959 | -0.000258024 | -0.023153135 | 0.036592972 | 0.017093948 | -0.018290484 | -0.005651224 | -0.001655942 | 0.004082644 | 0.001344987 | -0.000414421 | 0.008883834 | -0.00344555 | 0.00516239 | 0.00866085 | -0.000852621 | -0.007953298 |
| PC10 | -0.028338149 | -0.03033406 | 0.046459515 | -0.031320237 | -0.031804831 | -0.009807867 | -0.000922231 | 0.002781925 | 0.003108495 | 0.00304185 | 0.004476904 | -0.004682627 | -0.011963009 | -0.007801474 | -0.007218994 | -0.008265988 | 0.004763035 | 0.011019318 | -0.004651536 | 0.003142201 | 0.018713595 |
| PC11 | 0.01354502 | 0.045425228 | -0.038934725 | 0.016182847 | 0.050474335 | 0.045896948 | 0.00069533 | -0.008217144 | -0.005310372 | -0.017678176 | -0.00441792 | 0.012105768 | 0.024113034 | -0.006850829 | -0.004658786 | 0.013995477 | -0.005911996 | -0.011468117 | 0.009632728 | -0.00797079 | -0.036119184 |
| PC12 | 7.68E-05 | 0.029034915 | -0.029498152 | 0.025210716 | 0.039185908 | 0.028305279 | 0.001359425 | 0.002193185 | -0.0111762 | 0.008317566 | -0.003127477 | 0.004901985 | 0.003650482 | 0.015337122 | 0.004650394 | 0.018316119 | 0.000825148 | -0.008412421 | -0.00481923 | 0.000902894 | -0.004231949 |
| PC13 | -0.00200944 | -0.004638374 | -0.022153337 | 0.024137115 | 0.032636599 | -0.002642333 | -0.013591549 | 0.015200401 | -0.012469519 | 0.008845461 | 0.001756811 | -0.011999077 | -0.016092111 | 0.017467005 | -0.005103345 | 0.005688744 | -0.00642716 | 0.010558931 | -0.002117516 | 0.002774149 | 0.006080896 |
| PC14 | 0.015077024 | 0.033891154 | -0.001984722 | -0.001468592 | 0.004958357 | -0.01005853 | 0.009918436 | -0.015291315 | 0.010939551 | 0.042859919 | 0.005799668 | -0.001492991 | -0.021728894 | 0.018128438 | 0.026231573 | 0.024409968 | 0.002224142 | -0.019005156 | -0.005646759 | 6.90E-05 | -0.008296201 |
| PC15 | 0.006062132 | -0.007000977 | -0.0166006 | 0.022367312 | -0.009086996 | -0.016257356 | 0.000667072 | 0.016889077 | 0.001910689 | 0.009580208 | -0.015512729 | 0.002311033 | -0.013790125 | 0.007845788 | -0.018837339 | 0.002952222 | -0.008033822 | 0.020252117 | 0.00753184 | 0.004791356 | 0.004310047 |
| PC16 | -0.020041077 | -0.036978128 | -0.018876794 | 0.004238876 | -0.009844487 | -0.013945277 | 0.00487949 | 0.017225043 | 0.014831141 | 0.004132658 | -0.011530513 | -0.001400267 | 0.003896457 | -0.000923035 | -0.02857945 | 0.000485377 | -0.011457242 | 0.013621285 | 0.0208678 | 0.001433712 | -0.028155776 |
| PC17 | -0.011336471 | 0.001794498 | -0.018657789 | 0.003723573 | -0.067593192 | -0.034010786 | 0.023915902 | 0.02112169 | 0.042291134 | -0.003360515 | -0.042929802 | 0.042426291 | 0.033764157 | -0.01073568 | -0.001113519 | 0.023982979 | 0.005781009 | -0.019665392 | 0.00411331 | 0.00520616 | -0.002514342 |
| PC18 | -0.005218486 | -0.005815145 | -0.009378923 | 0.011683163 | -0.00354772 | -0.014799157 | 0.004423663 | 0.011204569 | 0.00042425 | 0.004214938 | -0.00439396 | 0.008722175 | 0.018317934 | -0.000303154 | -0.006061022 | -0.004137179 | 0.001302111 | 0.002942712 | 0.004207695 | 0.005270368 | 0.004804111 |
| PC19 | 0.013393978 | 0.001169499 | -0.002615349 | 0.012630106 | 0.071205585 | -0.004748644 | -0.017526532 | -0.031062544 | -0.045436824 | 0.0005041 | 0.025669174 | -0.040241638 | -0.018740497 | 0.001458768 | 0.001792285 | -0.027322919 | -0.003662904 | 0.025406402 | -0.006308813 | 0.004490128 | 0.017565692 |
| PC20 | -0.00594208 | -0.051792345 | 0.005874272 | 0.002716256 | -0.029068932 | -0.049170248 | -0.010352067 | 0.01786397 | 0.010744981 | 0.016190475 | -0.016667101 | 0.019682599 | 0.001270647 | 0.004681949 | 0.004049469 | -0.004873141 | -0.00141234 | -0.01352919 | -0.001982497 | 0.000839253 | -0.007269607 |
| PC21 | 0.004675684 | -0.052277214 | 0.017917629 | -0.001497763 | 0.018064698 | -0.009354515 | -0.010145272 | 0.008694835 | -0.003536451 | 0.023503372 | 0.012221628 | -0.000105625 | -0.010831522 | 0.008459309 | 0.001796938 | -0.010987191 | 0.002084127 | -0.009962807 | 0.002855245 | -0.001734719 | -0.014333594 |

|  | 2422 | 2423 | 2438 | 2439 | 2676 | 2677 | 2678 | 2693 | 2694 | 2695 | 2710 | 2711 | 2712 | 2932 | 2933 | 2948 | 2949 | 2950 | 2965 | 2966 | 2967 |
| --- | --- | --- | --- | --- | --- | --- | --- | --- | --- | --- | --- | --- | --- | --- | --- | --- | --- | --- | --- | --- | --- |
| PC22 | 0.008813908 | -0.004063243 | 0.010909353 | -0.014537224 | -0.005147225 | 0.01378915 | 0.012965996 | -0.028755435 | 0.007905514 | -0.002195378 | -0.003960222 | 0.002658369 | -0.00345199 | -0.014275183 | -0.004243761 | -0.001356422 | 0.014532805 | -0.008855353 | -1.92E-05 | -0.011781429 | -0.004254769 |
| PC23 | -0.010770713 | -0.022835289 | -0.003627173 | -0.010507995 | -0.026243332 | -0.0052203 | 0.013827811 | -0.016192125 | 0.005313509 | 0.00691971 | -0.006721284 | -0.000380136 | -0.002255012 | -0.015628912 | -0.017405761 | -0.002967978 | 0.009778853 | 0.003625535 | 0.007849525 | -0.004809812 | -0.003089721 |
| PC24 | -0.021598988 | 0.010990177 | 0.031881753 | -0.04576955 | -0.00660151 | 0.013396598 | 0.006519613 | -0.009589134 | 0.035707755 | 5.81E-05 | 0.004850485 | -0.011959777 | 0.029538888 | -0.005438011 | -0.008081795 | -0.026902882 | -0.007065835 | 0.007768994 | -0.018127739 | -0.009193925 | 0.015042897 |
| PC25 | -0.018331429 | -0.017033908 | 0.008749009 | -0.013903934 | -0.026157914 | 0.004566974 | 0.003741302 | -0.008279377 | -0.002304249 | -0.014672722 | -0.008972949 | 0.006722421 | 0.023505484 | -0.016074266 | -0.016370623 | -0.031393257 | 0.007270582 | 0.003097856 | -0.007791316 | -0.002969415 | 0.013297934 |
| PC26 | 0.035781024 | 0.0106058 | 0.01305865 | -0.036019785 | -0.067806802 | -0.014210707 | 0.011440473 | 0.001147172 | 0.022525423 | -0.019472976 | 0.002276868 | -0.001405723 | -0.013074157 | -0.008618596 | 0.008977424 | 0.012149845 | -0.005553898 | -0.004832684 | -0.004179027 | -0.009218841 | -0.006105465 |
| PC27 | 0.03743579 | 0.00055796 | 0.017470584 | -0.031785044 | -0.033949196 | -0.0062385 | -0.003028205 | -0.013035549 | 0.018317087 | -0.032147155 | -0.008760401 | -0.012805512 | -0.023799661 | -0.007566134 | 0.006644953 | 0.002951225 | -0.003011264 | 0.005311547 | -0.006801431 | -0.003952944 | -0.002874133 |
| PC28 | 0.047815524 | -0.002961889 | 0.030900164 | -0.018732217 | 0.0048593 | 0.018994246 | -0.001966778 | -0.040383039 | 0.008676335 | -0.044840606 | -0.005693411 | -0.020735134 | -0.037506156 | -0.011681183 | -0.000822164 | 0.002495445 | 0.008462946 | 0.010913205 | 0.004653629 | -0.000755377 | 0.010905104 |
| PC29 | 0.000670868 | -0.033163684 | 0.015408118 | 0.018766462 | -0.017255304 | -0.002002942 | -0.000761793 | 0.036525308 | 0.004923077 | 0.018844961 | 0.010748039 | -0.005714062 | 0.009129792 | 0.015470341 | -0.015838723 | 0.003093977 | 0.000468043 | 0.012710417 | -0.010323552 | 0.007742359 | 0.01305519 |
| PC30 | -0.031911565 | 0.017822232 | 0.028859784 | -0.028316677 | 0.017306074 | 0.004369521 | 0.01422256 | -0.002175422 | 0.01853515 | -0.004780699 | 0.01079042 | -0.01092413 | 0.011996219 | 0.014193366 | -0.007755948 | 0.011816869 | -0.018507381 | -0.002684769 | 0.014175248 | 0.004534696 | 0.016464563 |
| PC31 | -0.024159105 | 0.010381146 | 0.024467392 | -0.030001944 | -0.016889632 | 0.021546115 | -0.002643809 | -0.040435107 | -0.025740196 | -0.002019261 | 0.019551335 | 0.01103873 | 0.020538872 | -0.042669013 | 0.031182546 | -0.030679725 | 0.021493298 | -0.012856697 | 0.004910914 | 0.00336581 | -5.57E-05 |
| PC32 | -0.030276009 | -0.003653385 | 0.039833986 | -0.068018144 | 0.025022219 | -0.007200827 | 0.004447827 | 0.033080404 | 0.054167255 | 0.015494437 | 0.014941537 | -0.027909555 | 0.003358259 | 0.032711183 | -0.018383167 | 0.022956651 | -0.043111546 | 0.005192984 | 0.014987054 | 0.005980353 | -0.012998572 |
| PC33 | -0.003009052 | 0.008876053 | 0.033335603 | -0.027121666 | -0.007735798 | 0.030939021 | -0.025438694 | -0.010651716 | -0.005703364 | -0.0082742 | 0.010789073 | 0.0028567 | 0.027626491 | -0.007519819 | 0.043770355 | -0.004517028 | 0.001169647 | 0.004653507 | 0.003325822 | 0.009423353 | -0.011649681 |
| PC34 | 0.033027832 | 0.027422343 | -0.007298956 | 0.019772043 | -0.018244907 | -0.0089872 | -0.011575643 | 0.011385917 | -0.045505883 | -0.064719178 | -0.004409983 | 0.020042455 | 0.020244427 | 0.07687788 | 0.009386466 | 0.000466834 | -0.013015837 | -0.035667334 | -0.00090485 | -0.014388723 | 0.008086965 |
| PC35 | -0.020106934 | 0.007506161 | 0.01577605 | 0.00342708 | -0.032746922 | -0.004057069 | -0.000383807 | 0.021155147 | -0.000809607 | 0.030925864 | 0.024866729 | 0.002619365 | 0.003291657 | 0.009779833 | 0.000329625 | -0.005363286 | -0.005143009 | 8.20E-06 | 0.00163052 | -0.008820929 | 0.001701474 |
| PC36 | 0.013447578 | 0.077533371 | -0.012766483 | -0.033970446 | 0.027905803 | 0.033613705 | -0.000777173 | -0.031189833 | -0.002455223 | -0.024119544 | 0.005566871 | -0.022640545 | -0.017087147 | 0.000209423 | -0.001376645 | 0.008757268 | -0.014014842 | -8.34E-05 | -0.007738649 | -0.010407255 | -0.01090403 |
| PC37 | -0.054024589 | -0.038824609 | 0.004842295 | 0.04733263 | -0.019085374 | -0.026997028 | -0.018541919 | 0.024563269 | 0.00469311 | 0.045853491 | -0.008588123 | 0.00837442 | 0.011713092 | 0.016459985 | 0.007971828 | -0.026436997 | 0.008714127 | 0.002760497 | -1.39E-05 | 0.007585629 | 0.008611544 |
| PC38 | -0.020889992 | 0.031927135 | -0.028092955 | 0.017409383 | 0.018772858 | 0.037524154 | -0.007596559 | -0.000981836 | 0.001200248 | 0.009814439 | -0.016180038 | 0.002368943 | 0.012165234 | 0.010413077 | 0.003839682 | -0.005015063 | 0.009707957 | -0.000423794 | -0.002380302 | 0.006446744 | -0.003549521 |
| PC39 | -0.006387366 | 0.039021845 | -0.052504642 | 0.025149283 | 0.048722744 | 0.041941456 | -0.009770629 | 0.000215756 | -0.02904791 | 0.014669966 | -0.004947548 | -0.011748372 | 0.021308915 | 0.003672329 | 0.002332272 | -0.006377321 | -0.00036126 | 0.017644976 | -0.003732993 | 0.004722269 | 0.009554752 |
| PC40 | 0.009903784 | 0.020562474 | 0.006889239 | 0.027250042 | -0.041118381 | 0.018603719 | -0.006668326 | 0.02742696 | -0.010633619 | 0.037981746 | 0.032991127 | -0.001343224 | 0.022521968 | 0.009609017 | 0.014433298 | -0.00071365 | 0.004225196 | 0.003699723 | 0.000629959 | -0.007597264 | -0.01108315 |
| PC41 | -0.035830369 | 0.042991678 | -0.062027979 | -0.006508961 | 0.033639907 | -0.058202227 | -0.000729727 | -0.005960628 | -0.00585493 | -0.02464649 | -0.04387589 | 0.017799422 | -0.030893056 | -0.01232965 | -0.029233832 | -0.014239032 | 0.004476337 | -0.029768596 | -0.001562554 | 0.00099503 | 0.003157573 |
| PC42 | -0.032923883 | -0.006829172 | 0.011567799 | 0.016538742 | -0.007918983 | -0.008598164 | -0.018848083 | 0.017855941 | -0.059411444 | 0.010419469 | -0.004339813 | 0.050095835 | -0.045779925 | -0.028621751 | -0.006970264 | -0.010195152 | 0.024895095 | -0.00060323 | 0.014842004 | -0.009547282 | -0.005670596 |

|  | 2422 | 2423 | 2438 | 2439 | 2676 | 2677 | 2678 | 2693 | 2694 | 2695 | 2710 | 2711 | 2712 | 2932 | 2933 | 2948 | 2949 | 2950 | 2965 | 2966 | 2967 |
| --- | --- | --- | --- | --- | --- | --- | --- | --- | --- | --- | --- | --- | --- | --- | --- | --- | --- | --- | --- | --- | --- |
| PC43 | -0.011518707 | -0.005636922 | 0.0028073 | 0.018777915 | -0.017469506 | 0.012361738 | -0.001516317 | 0.003429579 | -0.032463475 | 0.014147039 | 0.001777316 | 0.015913441 | -0.025502201 | -0.037937595 | 0.028126353 | 0.023370205 | -0.012553983 | 0.012303632 | 0.006635799 | 0.031356478 | -0.005935475 |
| PC44 | 0.050933582 | -0.093398109 | -0.01682496 | 0.040086302 | 0.001725603 | 0.032985109 | -0.01425891 | 0.023643012 | 0.003103943 | -0.008506223 | -0.032852443 | -0.003583894 | -0.00495475 | 0.017739853 | -0.052576571 | 0.012948123 | 0.025556331 | 0.001393529 | -0.051026717 | -0.006359977 | 0.012945938 |
| PC45 | 0.032224636 | -0.023196847 | -0.010424978 | 0.033992515 | -0.005678644 | 0.026074385 | -0.024216957 | 0.009511894 | 0.017989092 | 0.004461268 | -0.00559896 | -0.012203505 | -0.013974294 | -0.02943873 | 0.000880866 | 0.02785871 | 0.003956948 | 0.028600103 | -0.016025382 | 0.009001182 | 0.001479257 |
| PC46 | 0.044193068 | 0.028470288 | -0.025591963 | 0.01420795 | -0.004727362 | -0.030924497 | 0.019287037 | -0.017898187 | 0.001710851 | 0.002022161 | 0.000716249 | -0.006316277 | 0.000367951 | 0.039228562 | -0.032354238 | 0.006335508 | -0.028082895 | 0.008197909 | 0.00831078 | 0.001383755 | -0.007502674 |
| PC47 | -0.022564044 | -0.068947805 | -0.026667444 | -0.011916156 | 0.011136171 | 0.013176573 | 0.011416333 | 0.04371226 | -0.050539983 | 0.033198814 | 0.041687639 | -0.003684003 | -0.004569758 | -0.009834642 | -0.020814648 | -0.007241247 | -0.012890569 | -0.006565157 | -0.006090122 | -0.015088348 | -0.008232304 |
| PC48 | -0.021745158 | 0.018125488 | 0.017256995 | 0.016356185 | 0.067128341 | 0.047342957 | 0.0010511 | -0.023021098 | 0.01430761 | -0.016289165 | -0.008823073 | 0.01302865 | 0.000401427 | 0.006981895 | -0.025364941 | 0.008670721 | 0.018744706 | -0.015605825 | 0.025319944 | 0.009256061 | 0.008819456 |
| PC49 | 0.037254758 | -0.062813094 | 0.04935674 | -0.028653749 | 0.006932358 | -0.026786916 | 0.004839934 | -0.017641944 | 0.001312897 | -0.034343534 | 0.004776878 | -0.009502309 | -0.022768359 | -0.01136797 | -0.01344725 | 0.010367333 | -0.006371008 | -0.020086421 | 0.002401842 | -0.002395834 | -0.008395851 |
| PC50 | 0.021460154 | -0.029939475 | 0.058498465 | 0.006338706 | 0.010596687 | -0.008189989 | -0.001168483 | -0.027590351 | 0.031332155 | -0.032412818 | -0.017671177 | 0.009366704 | -0.001925546 | 0.006683936 | 0.003384328 | 0.011854962 | 0.005884253 | -0.001255404 | 0.006259194 | 0.012471477 | 0.000964317 |
| PC51 | -0.007428637 | -0.001420114 | -0.015993585 | 0.011782362 | -0.003992707 | -0.010588335 | 0.010263745 | -0.017991412 | 0.043884575 | -0.01786292 | -0.036183928 | 0.018595242 | 0.021049649 | -0.000829051 | 0.028599332 | 0.010598579 | 0.007641404 | 0.025222133 | -0.008326826 | 0.008154377 | 0.007487819 |
| PC52 | 0.031636652 | 0.056685981 | -0.046388979 | -0.02415934 | 0.011052035 | -0.079981449 | 0.020269809 | 0.004150756 | -0.0213211 | 0.033093764 | 0.009186317 | 0.006200373 | 0.042766771 | -0.018529772 | 0.021601759 | -0.006004945 | -0.013836536 | 0.00753793 | -0.007852672 | 0.010478515 | 0.006761239 |
| PC53 | 0.036206226 | -0.009107753 | -0.027746819 | -0.055836919 | 0.002603776 | 0.036240432 | -0.038028631 | 0.001209046 | 0.004663631 | 0.003133625 | -0.044604085 | -0.022413834 | -0.033443947 | -0.000859671 | 0.005087737 | -0.020417362 | -0.010996545 | -0.011746333 | -0.004008554 | 0.000262457 | -0.010776316 |
| PC54 | -0.06232536 | -0.009567076 | -0.054163348 | 0.032602098 | -0.042485468 | 0.056992431 | 0.008360539 | -0.029122715 | 0.047732923 | -0.015600479 | 0.028661505 | -0.014088199 | -0.03712547 | 0.014215439 | 0.012947954 | -0.008523296 | -0.001370434 | 0.030802514 | 0.001337527 | -0.009036378 | -0.002501074 |
| PC55 | 0.008485652 | -0.05749565 | 0.002026206 | 0.031173677 | -0.045550378 | -0.028612321 | 0.032389981 | -0.051870694 | -0.007153851 | -0.03379496 | 0.0703915 | -0.020917203 | 0.003328822 | 0.036195812 | 0.011962372 | -0.008384804 | 0.010054663 | -0.011244429 | 0.003692693 | 0.007809804 | -0.013919141 |
| PC56 | -0.002586609 | -0.027220443 | -0.015232261 | 0.04177734 | 0.00372645 | 0.016639621 | 0.000249269 | 0.008546236 | -0.016337709 | -0.006964317 | -0.018900919 | 0.012488238 | -0.006846197 | 0.010646969 | 0.028610682 | -0.003639611 | 0.014294418 | 0.005775987 | 0.019133933 | -0.031009482 | -0.005323846 |
| PC57 | -0.023407756 | 0.014837694 | -0.032371191 | 0.013875937 | 0.016417736 | -0.03171638 | 0.026806926 | 0.021328846 | 0.018886142 | 0.022851728 | 6.91E-05 | -0.01252907 | -0.031913127 | -0.01950034 | 0.002148894 | 0.020558955 | -0.011980672 | 0.018034565 | 0.028454625 | 0.012286862 | -0.004514806 |
| PC58 | 0.035347298 | -0.047816558 | -0.040918272 | 0.016001305 | 0.01639244 | 0.045094928 | 0.01009528 | 0.001241034 | 0.039240687 | 0.022567297 | -0.041965805 | -0.043902232 | 0.057715739 | -0.003460285 | 0.02060872 | -0.011717026 | -0.004430576 | -0.016031183 | 0.007940558 | -0.006849816 | 0.017759175 |
| PC59 | 0.002488396 | 0.031006709 | -0.027802819 | -0.009608053 | 0.025964478 | -0.018936277 | 0.001185011 | -0.006038557 | 0.052439932 | 0.035818269 | 0.013185272 | -0.021866179 | -0.004526077 | -0.022455897 | 0.00437959 | 0.012200316 | 0.038206393 | -0.013966267 | 0.014558666 | -0.015155713 | -0.012736645 |
| PC60 | 0.035088933 | 0.051207229 | -0.007093081 | -0.003495067 | 0.013662643 | -0.029693144 | 0.012251861 | -0.015080427 | 0.051596851 | 0.030494353 | 0.028505852 | -0.013356965 | 0.003392432 | -0.023379418 | -0.037318608 | -0.043596425 | 0.014686945 | -0.018428572 | -0.038361704 | 0.008595192 | -0.006617139 |
| PC61 | 0.0030448 | -0.023909872 | -0.023372731 | -0.004672737 | -0.015712245 | -0.040027379 | 0.017222084 | -0.016306322 | 0.00238714 | -0.014028479 | 0.006078766 | -0.012503079 | 0.002202379 | 0.00471108 | 0.018640961 | -0.003827609 | 0.003365492 | 0.010865899 | 0.005390512 | 0.008564149 | 0.015320892 |
| PC62 | 0.023513937 | -0.016766642 | -0.004127365 | 3.62E-05 | 0.043245619 | 0.009497096 | 0.028500367 | 0.00548948 | -0.008585628 | 0.005437066 | 0.0402128 | 0.025438308 | 0.011469967 | 0.017711374 | -0.011869681 | -0.008484592 | 0.005642892 | -0.003358053 | 0.000754491 | 0.019695615 | -0.007607061 |
| PC63 | 0.014949894 | 0.000255428 | -0.006161118 | -0.0080818 | 0.014806578 | 0.043330707 | 0.028340686 | 0.037978845 | -0.016862597 | 0.020537334 | 0.039490374 | 0.026729616 | -0.002807292 | 0.009098436 | -0.015807624 | -0.013060257 | 0.001287055 | -0.006485527 | 0.002116331 | -0.002966065 | -0.001262797 |

|  | 2422 | 2423 | 2438 | 2439 | 2676 | 2677 | 2678 | 2693 | 2694 | 2695 | 2710 | 2711 | 2712 | 2932 | 2933 | 2948 | 2949 | 2950 | 2965 | 2966 | 2967 |
| --- | --- | --- | --- | --- | --- | --- | --- | --- | --- | --- | --- | --- | --- | --- | --- | --- | --- | --- | --- | --- | --- |
| PC64 | 0.00151539 | -0.005874961 | -0.028552878 | -0.02178295 | -0.054371291 | 0.032787668 | 0.022088056 | 0.038959569 | -0.012495114 | 0.001320281 | 0.012772129 | -0.007231379 | -0.013956055 | -0.020526575 | -0.004962047 | 0.014846817 | -6.04E-05 | -0.003014823 | -0.003995686 | -0.03519841 | 0.008171332 |
| PC65 | 0.064097802 | 0.038048595 | 0.055804527 | -0.052933274 | -0.020683794 | 0.015707612 | -0.022249399 | 0.026212032 | -0.024327983 | 0.008247607 | 0.01027351 | -0.013266684 | 0.025033907 | -0.020427027 | -0.016264245 | 0.04365245 | -0.012929363 | 0.012396051 | -0.014010125 | -0.016802678 | 0.002275549 |
| PC66 | -0.033336348 | 0.010905171 | 0.091022016 | 0.055072802 | 0.039120456 | -0.075892453 | -0.087766858 | -0.034069078 | -0.025017389 | 0.024328116 | -0.025830166 | -0.030879613 | 0.02892402 | -0.012721194 | 0.000305926 | 0.014840484 | -0.020297059 | 0.004839845 | -0.020907505 | -0.022992408 | -0.017485372 |
| PC67 | -0.061107529 | -0.000523369 | 0.038416895 | 0.046622149 | 0.024216101 | 0.003642985 | -0.000813933 | -0.016068599 | -0.018652288 | -0.00673243 | 0.007261246 | -0.01600346 | 0.009094257 | -0.02900775 | -0.027100727 | 0.031656683 | 0.014450387 | -0.018783594 | 0.03519251 | -0.01219051 | 0.032132089 |
| PC68 | -0.047842183 | 0.041023759 | 0.0909231 | 0.016812198 | 0.015481203 | 0.050161765 | 0.034067736 | 0.028962717 | -0.0055093 | -0.012262208 | -0.028223762 | 0.025856302 | -0.00807123 | -0.017024535 | -0.022283378 | -0.001341021 | -0.013565937 | -0.010890228 | -0.028928145 | 0.048331194 | -0.013670039 |
| PC69 | -0.085841484 | 0.061424127 | 0.004986479 | -0.064752243 | 0.005067253 | -0.008969091 | -0.000655148 | -0.00066782 | -0.016849564 | -0.012551613 | -0.026100637 | 0.013188608 | -0.006452926 | 0.022479715 | 0.007267053 | -0.031891705 | -0.022728467 | 0.007784158 | -0.001061217 | -0.010521256 | 0.009431999 |
| PC70 | -0.079707519 | 0.066555647 | 0.02572939 | -0.035379285 | 0.016990505 | -0.035062021 | 0.019840712 | 0.03868885 | 0.00824503 | -0.011755514 | 0.000189243 | 0.003157229 | -0.026341684 | 0.027724665 | -0.002505468 | 0.002633148 | 0.023228218 | 0.015942368 | -0.003994979 | -0.020866448 | 0.015663535 |
| PC71 | 0.107936102 | -0.026066135 | 0.041707278 | -0.054894914 | 0.050390895 | -0.018765543 | 0.012171488 | 0.053556621 | -0.012446678 | 0.011265411 | -0.021989474 | 0.018621158 | -0.016470993 | -0.00420938 | 0.029987409 | -0.014021168 | 0.008303702 | 0.001893801 | 0.01582344 | -0.015895851 | 0.002840932 |
| PC72 | -0.003290379 | -0.04984817 | -0.093790183 | -0.060553212 | -0.027479833 | -0.017591111 | -0.064776952 | -0.044666693 | -0.031801465 | -0.003871636 | 0.01609262 | 0.018390093 | 0.003570412 | -0.006183026 | -0.00897816 | 0.016314827 | -0.018961911 | 0.020246419 | 0.000351916 | 0.022476482 | 0.004226117 |
| PC73 | -0.00242171 | 0.006234338 | -0.022739265 | -0.024418896 | -0.026864872 | -0.002081436 | -0.061720301 | -0.026983916 | -0.028212223 | -0.024741293 | 0.024291207 | 0.022526615 | 0.02600025 | 0.015282179 | -0.022835338 | 0.033899234 | 0.044129469 | 0.006755687 | -0.014709675 | 0.003400794 | -0.016618297 |
| PC74 | 0.037114732 | -0.025661932 | 0.042851005 | 0.063500584 | -0.042092537 | 0.058247933 | -0.022899826 | -0.018248837 | -0.00722566 | 0.003885613 | -0.0056975 | 0.028423225 | -0.000804144 | -0.005880292 | 0.011046343 | -0.010284945 | -0.064641909 | -0.020875687 | 0.01327795 | 0.001942327 | 0.017353268 |
| PC75 | -0.00848886 | 0.058193253 | -0.079198015 | -0.01639762 | -0.04218155 | 0.04147845 | 0.002820203 | 0.01992247 | 0.001302448 | 0.007215944 | -0.032523821 | -0.004356301 | 0.012865469 | -0.014472452 | 0.00892566 | 0.013913201 | -0.001250431 | 0.000271326 | -0.004859353 | -0.008617264 | 0.000945542 |
| PC76 | -0.024672744 | 0.044410763 | 0.072470923 | 0.062188667 | -0.022144923 | 0.004217213 | -0.024516782 | -0.015159056 | 0.047046506 | 0.008296203 | 0.011090672 | 0.023565712 | -0.011107784 | 0.015024068 | 0.003999671 | -0.008674066 | 0.011013587 | 0.014186156 | 0.010495452 | 0.000634975 | -0.00221451 |
| PC77 | -0.025212202 | -0.014327609 | 0.001722641 | 0.016636213 | -0.014786521 | -0.040068652 | 0.002776597 | 0.001173421 | -0.011150698 | -0.009417749 | -0.022485208 | -0.023372364 | -0.021778016 | -0.000550327 | 0.013186534 | -0.02297098 | -0.016175041 | -0.015949211 | -0.01254036 | -0.00188887 | -0.010821253 |
| PC78 | -0.024928824 | -0.052959427 | -0.093665478 | -0.004438023 | -0.001218086 | -0.008065594 | -0.005717725 | -0.003978499 | -0.021213242 | -0.00626638 | -0.038989376 | -0.039735155 | -0.018081156 | 0.004406515 | 0.010805876 | -0.016924534 | -0.003259092 | -0.018584969 | 0.000300691 | 0.015766146 | -0.009664643 |
| PC79 | 0.07750044 | 0.004658228 | 0.012788842 | -0.039357826 | 0.002188206 | 0.004862232 | -0.10172086 | -0.001364352 | 0.032793953 | 0.040665026 | -0.018752742 | 0.003230432 | 0.002556343 | 0.038809337 | -0.01030586 | -0.018989671 | 0.035752692 | 0.00515986 | 0.039829469 | 0.015471401 | 0.002302582 |
| PC80 | 0.053237805 | 0.02883165 | -0.015285061 | -0.076973919 | -0.030491215 | 0.024517711 | 0.004462293 | 0.026065165 | 0.001958924 | 0.011025954 | 0.01818328 | 0.006719299 | -0.011957147 | 0.01175791 | -0.012014748 | -0.010598423 | 0.008653033 | -0.009730293 | 0.010906049 | 0.024816492 | 0.012252551 |
| PC81 | 0.000772741 | -0.041601948 | -0.039906186 | -0.006792753 | 0.05492159 | -0.038689706 | 0.075036073 | -0.018829064 | -0.000200275 | -0.037708146 | 0.008644162 | 0.02362821 | 0.036365172 | -0.001544621 | 0.005933488 | 0.02402458 | 0.008911133 | 0.021296971 | -0.002195052 | -0.000429755 | 0.004740645 |
| PC82 | -0.002547193 | -0.017230307 | -0.03101071 | 0.031234759 | 0.034805842 | 0.019480518 | -0.010990343 | -0.029273518 | 0.076031368 | 0.014584857 | 0.016253017 | 0.062880945 | -0.023106489 | 0.008300393 | 0.011249704 | -0.018731786 | -0.019906609 | 0.012424884 | -0.028385645 | -0.026854286 | -0.010157825 |
| PC83 | 0.012546714 | -0.035047433 | -0.033767855 | 0.031921968 | 0.015167558 | -0.010052533 | -0.004183226 | -0.022871088 | 0.038942349 | 0.020051544 | 0.038165935 | -0.011199314 | -0.004481714 | -0.031748473 | -0.015163331 | 0.012517202 | -0.018326277 | -0.045623503 | 0.012923531 | -0.002633006 | 0.016755165 |
| PC84 | 0.039046021 | -0.027963302 | 0.061901958 | 0.039244931 | 0.001565665 | 0.013429783 | 0.074646872 | 0.038326953 | -0.021560354 | -0.012437566 | -0.029682024 | -0.055864441 | 0.008392193 | 0.009926824 | 0.01916972 | -0.012778568 | 0.027625327 | 0.014628482 | 0.002869752 | 0.008356506 | -0.022528585 |
| PC85 | -0.083348565 | 0.064536466 | -0.011017939 | 0.027911448 | -0.087520071 | 0.015368291 | -0.01167486 | 0.003591292 | -0.012123868 | 0.001546257 | -0.001299175 | -0.059459489 | 0.002759235 | 0.015561185 | -0.021596286 | 0.00141833 | 0.01552985 | -0.015350765 | 0.005341159 | 0.009728003 | -0.006526768 |

Color number value 2968-3494:

|  | 2968 | 2983 | 2984 | 2985 | 3204 | 3205 | 3220 | 3221 | 3222 | 3223 | 3237 | 3238 | 3239 | 3240 | 3241 | 3256 | 3257 | 3258 | 3492 | 3493 | 3494 |
| --- | --- | --- | --- | --- | --- | --- | --- | --- | --- | --- | --- | --- | --- | --- | --- | --- | --- | --- | --- | --- | --- |
| PC1 | -0.008145079 | -0.002217306 | 0.004585464 | 0.005081435 | 0.001932962 | -0.00104224 | -0.001123937 | 0.003686456 | -0.002394316 | -0.002136648 | -0.006458354 | -0.002709374 | -0.001435058 | 0.001880246 | 0.002352405 | 0.002153457 | -0.000206999 | -0.001880558 | 0.000367155 | 0.001597187 | -0.000476446 |
| PC2 | -0.000903524 | 0.014629664 | -0.006289615 | -0.000259473 | 0.00055875 | -0.007495785 | 0.009926525 | 0.005219726 | 0.006068852 | 0.013818345 | 0.00535984 | -0.001219503 | -0.018676723 | -0.004860772 | -0.004037328 | 0.002800323 | -0.003964332 | 0.006056586 | -0.001765746 | -0.000925853 | -0.001051219 |
| PC3 | -0.000843917 | 0.006611528 | 0.000801288 | -0.00157643 | -0.002386176 | 0.003887843 | 0.000518509 | 0.001732594 | 0.005590971 | 0.005551792 | 0.005766922 | 1.39E-05 | 6.98E-05 | -0.000666951 | -0.003793697 | -0.000677852 | 0.002400868 | 0.000927574 | -0.000482496 | -0.003798756 | -0.001247718 |
| PC4 | 0.007603079 | 0.002919467 | -0.007129452 | -0.00323079 | -0.00217958 | -0.004799176 | -0.001637163 | -0.007627756 | -0.010063224 | -0.019680494 | 0.010073416 | 0.008972292 | 0.013856681 | 0.002036346 | 0.005841882 | 0.001955094 | 0.000658441 | 0.008474867 | 0.002324973 | -0.00019316 | 0.002876787 |
| PC5 | -0.001388591 | 0.00345937 | 0.000437634 | 0.000713395 | -0.002345608 | -0.002234295 | 0.000505372 | -0.003065528 | -0.003827827 | -0.004000383 | -0.002067395 | 0.004449831 | 0.008599903 | 0.003617376 | 0.004200041 | -0.001599086 | 0.001585913 | -0.001375974 | 0.000967467 | -0.00169207 | -4.48E-05 |
| PC6 | 0.002434428 | -0.009401782 | 0.005109028 | -0.001841714 | 0.000938628 | 0.007346409 | -0.00269119 | 0.00449796 | 0.001166673 | 0.009501847 | -0.003894274 | -0.001630101 | 0.000664423 | 0.00057479 | -0.002722951 | -0.005248869 | 0.002357013 | -0.006050796 | 0.000559289 | 0.000701325 | -0.001701258 |
| PC7 | 0.009493967 | 0.009846293 | -0.003167228 | 0.00065897 | -0.000157796 | 0.008432723 | 0.005583341 | -0.007761664 | 0.000819363 | -0.009676218 | 0.0071387 | -0.00196327 | 0.004764926 | -0.00524992 | -0.002463683 | -0.001602089 | 0.000439326 | 0.001279215 | 0.000254022 | 0.001043311 | -0.001735952 |
| PC8 | 0.003797336 | -0.015771771 | 0.003442303 | -0.003599757 | 0.003533287 | 0.003201061 | -0.005917301 | 0.002876473 | -0.006182561 | -0.000577086 | -0.005200176 | -0.001516511 | 0.002370833 | 0.00418027 | -0.001489624 | -0.004299394 | 0.001955714 | -0.004852644 | -0.000562304 | -0.000769683 | 7.71E-05 |
| PC9 | -0.006612029 | -0.017516922 | 0.006604241 | -0.002408472 | -0.000339322 | -0.000794644 | -0.007537262 | 0.000472103 | 0.001423503 | 0.003726761 | -0.00910227 | -0.001001433 | 0.002221578 | 0.000428547 | -0.000519713 | 0.002521758 | -0.002425072 | -0.005183657 | -0.000522957 | 0.000510077 | 0.003394356 |
| PC10 | -0.002355116 | -0.010792898 | -0.001789111 | -0.006770842 | 0.00098378 | 0.000574789 | 0.005126046 | 0.000938685 | -0.005421742 | 0.004664415 | 0.001460375 | -0.002706464 | -2.16E-05 | 0.000261963 | 0.004448357 | -0.002963029 | -0.003309916 | 0.003944165 | 0.002544495 | -0.001310701 | -0.002490815 |
| PC11 | 0.006073307 | 0.018244883 | 0.000312118 | 0.009859991 | -0.003434232 | 0.001151003 | -0.010812169 | -0.003312189 | 0.006988863 | -0.010988519 | -0.003086057 | -0.004172659 | 0.001827764 | -0.002806686 | -0.002940121 | 0.00220019 | -0.002647809 | -0.00175042 | 0.001920497 | 0.005093878 | 0.005127508 |
| PC12 | 0.004592628 | -0.001770729 | -0.005292734 | 0.001966873 | 0.00533923 | -0.000467828 | -0.008952261 | 0.00407721 | 0.004304566 | -0.005251415 | 0.000750597 | 0.005328422 | 0.001082238 | 0.005192324 | -0.003875523 | 0.001595688 | 0.008042377 | -0.00364455 | -0.004957222 | -0.00057055 | -0.000883717 |
| PC13 | -0.011145232 | -0.012813879 | 0.002547553 | 0.001917494 | 0.007362921 | 0.001019288 | 0.000941492 | 0.005470522 | 0.003492268 | 0.006756967 | -0.001255677 | -0.002758774 | -0.007909333 | -0.000805735 | -0.001081959 | 0.000524714 | -0.00623461 | -0.002744687 | -0.001789758 | 0.003296876 | 0.006042426 |
| PC14 | 0.001573658 | -0.005230204 | -0.003312286 | -0.004253801 | -0.002106915 | -0.008404279 | 0.000969015 | -0.000118054 | 0.004908736 | 0.017482902 | 0.004501616 | 0.003368824 | -0.010350294 | -0.004979993 | 0.000693291 | 0.006656156 | -0.004767794 | 0.005494257 | -0.001145333 | -0.002879406 | 0.002478956 |
| PC15 | -0.000183394 | -0.016698135 | -0.000244213 | -0.00482952 | -0.001152315 | -0.012112153 | -0.005515163 | 0.008308261 | -0.014366698 | 4.94E-05 | -0.007268014 | -0.006413553 | -0.005330743 | 0.005582259 | 0.009070772 | -0.000305495 | -0.000516433 | 0.004276084 | 0.001586395 | -0.004189309 | -0.000543354 |
| PC16 | -0.001597739 | 0.009791441 | -0.002262666 | 0.004958465 | -0.009530129 | -0.015296927 | -0.00028362 | -0.015121123 | 0.004823886 | -0.014509846 | 0.002848567 | -0.004485752 | 0.007224295 | -0.004466415 | -0.001463481 | 0.003888539 | 0.005314251 | 0.003025533 | -0.001015771 | 0.000769215 | -0.01008376 |
| PC17 | 0.016885619 | -0.004592692 | -0.009602909 | -0.002187985 | 0.001469308 | 0.005636807 | -0.004274293 | 0.005594384 | -0.009998485 | -0.000551838 | -1.55E-05 | 0.004320415 | -0.003997151 | -0.005914863 | -0.001430398 | 0.002936431 | 0.002712094 | -0.004565657 | 0.004284848 | -0.000157338 | 0.002048892 |
| PC18 | 0.003174112 | -0.000649924 | -0.006109739 | 0.002240446 | -0.001402063 | -0.002074493 | -7.22E-05 | 0.003974193 | -0.003662966 | -0.000399734 | -0.002301664 | -0.001637204 | -0.002763668 | 0.001137008 | -0.001190175 | 0.000351235 | 0.0046489 | -0.001693224 | -7.26E-05 | -0.000953292 | -0.002914793 |
| PC19 | -0.01106001 | -0.000672106 | 0.003277838 | 0.007791936 | 0.000334443 | -0.006472708 | 0.006084457 | -0.002165542 | 0.005397087 | -0.005576101 | 0.000824125 | -0.003045187 | 0.002366066 | -0.002573841 | 0.005188999 | -0.000368714 | 0.000282919 | 0.005704199 | -0.000890403 | 0.001991752 | 0.000736833 |
| PC20 | -0.003946514 | 0.000636693 | 0.007337791 | 0.004179693 | 0.001153015 | 0.004048568 | 0.006389671 | -0.005567381 | -0.006285297 | 0.001218554 | -0.004813601 | -0.000925154 | 0.004435473 | -0.005483664 | 0.009002742 | -0.003373727 | -0.005120328 | 0.002289339 | 0.001554536 | -0.010297975 | 0.001865001 |
| PC21 | -0.009332346 | 0.010963494 | 0.00951608 | -0.002866219 | -0.006801585 | 0.006129239 | 0.005184989 | -0.007699933 | 0.011218367 | 0.009977062 | 0.001474783 | 0.001566203 | 0.001563971 | 0.007032692 | -0.005757748 | 0.001471554 | -0.006405373 | 0.000501007 | -0.000425276 | -0.002029526 | 0.001779789 |

|  | 2968 | 2983 | 2984 | 2985 | 3204 | 3205 | 3220 | 3221 | 3222 | 3223 | 3237 | 3238 | 3239 | 3240 | 3241 | 3256 | 3257 | 3258 | 3492 | 3493 | 3494 |
| --- | --- | --- | --- | --- | --- | --- | --- | --- | --- | --- | --- | --- | --- | --- | --- | --- | --- | --- | --- | --- | --- |
| PC22 | 0.003763494 | 0.004178461 | 0.010625947 | -0.003323931 | -0.003049817 | 0.003695763 | -0.004180714 | 0.005827956 | 0.001452946 | 0.006569691 | 0.000394493 | 0.012027726 | 0.004380911 | 0.008928631 | -0.00683738 | 0.002212675 | 0.002063692 | 0.000304527 | 0.003428802 | -0.003294825 | -0.005891828 |
| PC23 | 0.003680803 | 0.005213067 | -0.001133562 | -0.002119018 | 0.004672379 | 0.004543369 | 0.00816242 | -0.00709475 | 0.004127963 | 0.000363043 | 0.004425439 | 0.005420724 | -0.003013254 | -0.012922131 | 0.001141484 | -0.005080205 | -0.003690368 | -0.004034789 | 0.004061277 | 0.007334883 | 0.001192987 |
| PC24 | -0.0073358 | 0.008980742 | 0.002879772 | 0.014918315 | -0.003738447 | 0.036436143 | 0.000696293 | 0.009951595 | 0.004046383 | 0.004394396 | -0.00847188 | -0.004577006 | 0.008939409 | 0.007955728 | -0.005602848 | 0.001817832 | 0.001641358 | -0.005718992 | -0.000563065 | 0.003241691 | -0.001686647 |
| PC25 | -0.000882807 | 0.014764006 | -0.010397566 | 0.008191242 | 0.004507507 | 0.012737987 | 0.005824379 | 0.000715363 | -0.004781988 | -0.008857023 | 0.002421142 | -0.008009766 | -0.002253051 | -0.001886674 | 0.004074002 | -0.002500181 | 0.000498372 | 0.006836215 | -0.002237722 | -0.001857161 | 0.008636183 |
| PC26 | -0.000199204 | 0.001450709 | 0.005775807 | -0.00600933 | 0.007736754 | 0.000310159 | -0.007411347 | 0.003286825 | 0.00054483 | -0.003643299 | -0.00048386 | -0.00069614 | 0.00022264 | -0.000217122 | 0.001019301 | 0.000283525 | 0.002647287 | 0.003236976 | -0.00376973 | 0.003047204 | 0.000115773 |
| PC27 | -0.001156969 | -0.002063385 | 0.004997873 | -0.001796233 | 0.006575935 | 0.002228892 | -0.004427056 | -0.000508857 | 0.000718344 | -0.001732732 | -0.003327497 | -0.005013776 | 0.001313021 | 0.004199325 | 0.003420959 | -0.001482355 | 0.00208866 | 0.002276757 | -0.005535967 | 0.002479424 | -0.002514169 |
| PC28 | 0.001632403 | -0.008038199 | 0.009858191 | -0.015616781 | -0.005291396 | 0.004880812 | -0.003834232 | -0.009071046 | -0.00689923 | 0.004130162 | -0.00199541 | 0.007578458 | -0.007280478 | 0.001253955 | 0.000287237 | -0.000946494 | 0.003016047 | -0.000787683 | 0.001412921 | 0.004530876 | -0.005014462 |
| PC29 | -0.001044093 | -0.003536502 | -0.007561284 | -0.002592889 | 0.006287446 | 0.002284839 | -0.003664022 | 0.006573351 | -0.002187273 | -0.008425469 | -0.002525417 | -0.001223708 | -0.001514347 | -0.00748174 | 0.001412388 | 0.001059578 | 0.004609445 | 0.005242162 | -0.006099001 | 0.007648937 | 0.004814109 |
| PC30 | -0.002393291 | 0.00678392 | -0.022431849 | -0.00584479 | -9.49E-05 | -0.013515144 | 0.005779776 | 0.021023486 | -0.007170104 | -0.000345103 | 0.016240992 | -0.004244096 | -0.00373811 | -2.59E-05 | -0.002223303 | 0.003724731 | 0.004728461 | -0.001499215 | 0.000118286 | -0.004701118 | -0.002817424 |
| PC31 | 0.011137442 | 0.005216787 | -0.004821389 | -0.003175775 | 0.004259215 | -0.018614462 | 0.001049179 | -0.003848887 | -0.006526081 | 0.002979941 | 0.002468385 | 0.007692224 | -0.001617042 | -0.003057492 | 0.00555206 | -0.011633819 | -0.000804178 | -0.010026152 | -0.003347029 | -0.003448033 | 0.000997415 |
| PC32 | -0.015317257 | -0.005291629 | -1.25E-05 | -0.005963734 | 0.007731567 | 0.001842078 | 0.002315944 | -0.013189996 | -0.000247037 | -0.004839538 | -0.003725145 | 0.003157459 | 0.001472692 | -0.002176395 | 0.002957619 | -0.00476532 | 0.000141947 | 0.003448153 | 0.003192507 | -0.001465331 | 0.000661477 |
| PC33 | 0.001332748 | -0.026088626 | 0.005552793 | -0.00106603 | -0.01102339 | -0.016216825 | -0.009998118 | -0.01274109 | -0.002689646 | -0.000284922 | -0.006045737 | 0.00034576 | -0.00672775 | -0.001128994 | 0.009120752 | 0.008179107 | -0.003676601 | -0.002754436 | -0.000653501 | 0.009483097 | -0.000829632 |
| PC34 | -0.007858259 | 0.001296649 | 0.003036753 | -0.005616146 | 0.017939245 | -0.001799644 | 0.012358473 | 0.000567167 | 0.006702946 | -0.003805575 | -0.005249519 | -0.005755127 | -0.004765852 | -0.00037326 | 0.001253333 | 0.000720488 | 0.000735469 | 0.002405801 | 0.003349006 | 0.003559769 | -0.004043567 |
| PC35 | -0.004926926 | 0.005477397 | -0.002054555 | -0.00887145 | -0.004388555 | -0.009067075 | 0.003930868 | -0.000449542 | 0.002504378 | 0.001291504 | 0.007037746 | 0.000548649 | 0.003477321 | -0.000125264 | -0.008174469 | 0.001118781 | -0.004998613 | -0.00107151 | -0.002360704 | 0.002249282 | -0.003665348 |
| PC36 | -0.000933974 | 0.009393738 | 0.00089994 | -0.003587165 | 0.00816721 | -0.002531568 | 0.004654238 | 0.00542031 | 0.000251357 | 0.001335998 | 0.003876212 | -0.004354178 | 0.00457209 | -0.001226822 | 0.001525494 | 0.002645744 | -0.007137144 | -0.001225769 | -0.002545773 | -0.006738996 | 0.003031448 |
| PC37 | -0.006305237 | -0.007823523 | -0.01163541 | 0.013985356 | -0.01486649 | -0.008898532 | 0.00707159 | -0.004508787 | 0.006206729 | 0.002900783 | 0.003936365 | 0.001697657 | 0.003045296 | 0.002502263 | -0.008204594 | -0.003108194 | -0.002368654 | 0.001681981 | 0.00011615 | 0.006316461 | -0.002608845 |
| PC38 | 0.002380015 | -0.004800221 | -0.01074626 | 0.012202108 | -0.004500026 | 0.003323835 | -0.002183102 | 3.19E-05 | 0.00208973 | 0.005147214 | -0.002641321 | -0.002630988 | 0.00134036 | 0.001041262 | 0.002649914 | -0.003239035 | 1.18E-05 | -0.000477227 | 0.000775003 | 0.004021207 | 0.002309872 |
| PC39 | -0.004844978 | -0.008526891 | -0.016660331 | 0.004852271 | 0.002807847 | -0.003972463 | -0.001025441 | -0.003024221 | -0.00911779 | 0.00446462 | -0.000923477 | -0.006992624 | 0.005014595 | -0.006251088 | 0.000396347 | 0.00024506 | -0.002856659 | -0.008880348 | 0.013359086 | -0.005604449 | -0.002476044 |
| PC40 | 0.000154933 | 0.004594648 | 0.014266207 | 0.005233928 | -0.007609037 | 0.012125445 | 0.001064241 | -0.01452733 | 0.000869461 | 0.007289969 | -0.007991414 | 0.005923609 | -0.003436224 | 0.003837042 | 0.004788226 | -0.001333351 | 0.002221396 | 0.003874053 | 0.001233784 | -0.005421477 | 0.003005861 |
| PC41 | 0.023602594 | -0.023155636 | 0.016357458 | 0.013438555 | -0.003894993 | 0.003934056 | 0.017078773 | -0.002639507 | 0.002275991 | -0.007189551 | 0.001084418 | 0.000541754 | 0.00077243 | 0.001000884 | 0.001229215 | 1.58E-05 | -0.008373908 | -0.000374688 | -0.004814489 | 0.000863575 | -0.003902999 |
| PC42 | -0.007312926 | -0.010203211 | -0.003302902 | -0.006976007 | 0.010907741 | -0.01036573 | -0.010240161 | 0.014553007 | 0.011735392 | 0.009350253 | 0.001590505 | 0.000578332 | 0.013460217 | -0.000238026 | -0.004505161 | 0.000988982 | 0.007516099 | 0.006741122 | 0.00185423 | 0.00286278 | 0.005383463 |

|  | 2968 | 2983 | 2984 | 2985 | 3204 | 3205 | 3220 | 3221 | 3222 | 3223 | 3237 | 3238 | 3239 | 3240 | 3241 | 3256 | 3257 | 3258 | 3492 | 3493 | 3494 |
| --- | --- | --- | --- | --- | --- | --- | --- | --- | --- | --- | --- | --- | --- | --- | --- | --- | --- | --- | --- | --- | --- |
| PC43 | 0.008932145 | 0.015433203 | 0.001203387 | 0.010222713 | 0.004728849 | -0.00555082 | 0.001478224 | -0.004046587 | -0.005096708 | -0.006750554 | -0.017625919 | -0.015001581 | -0.005408873 | 0.008093989 | -0.014060632 | -0.001155673 | 0.00027822 | 0.004914728 | -0.001054316 | -0.006786758 | -0.002317839 |
| PC44 | -0.00283411 | 0.026889023 | 0.001360164 | -0.002909338 | 0.006075361 | -0.013694332 | -0.010201188 | -0.019853032 | 0.004048099 | 0.004143682 | 0.00054555 | 0.003309379 | -0.005235937 | -0.001274766 | -0.000423736 | -0.005554134 | 0.002059938 | -0.005569508 | 3.84E-05 | -0.002514193 | 0.000571385 |
| PC45 | 0.008893311 | 0.000719088 | 0.014125257 | 0.012872678 | -0.028250277 | 0.01856231 | -0.008536742 | 0.013707183 | -0.001144923 | -0.002497724 | 0.012233053 | -0.001562266 | -0.005259891 | -0.012887039 | 0.002079622 | 0.006105684 | -0.005611693 | 0.004446055 | 0.00080911 | -0.00071651 | -0.00271163 |
| PC46 | 0.008090374 | -0.009908944 | 0.007895102 | -0.001806688 | -0.02439749 | -0.005766817 | -0.01108381 | 0.005098523 | -0.00172813 | 0.010183521 | 0.008334616 | 0.007868422 | 0.015458343 | 0.001776446 | 0.001176808 | -0.005376555 | 0.002816565 | -0.001705428 | -0.002841466 | -0.0034866 | 0.00457785 |
| PC47 | 0.018996498 | 0.002675697 | 0.015512776 | -0.006191261 | 0.013673362 | 0.003786765 | -0.00251504 | 0.006907 | -0.004024088 | -0.004824356 | 0.003686623 | 0.00031096 | -0.004069808 | -0.000231957 | 0.007346964 | 0.006408418 | 0.003757727 | -0.007418623 | -0.006137656 | -0.001882773 | -0.003121796 |
| PC48 | 0.007605762 | -0.005747418 | -0.00523622 | 0.001950823 | -0.012568268 | 0.011018837 | 0.004729106 | -0.007878996 | -0.002461252 | 0.00096712 | -0.003709561 | 0.009262621 | -0.01228154 | 0.006358828 | 0.001081444 | -0.001717078 | 0.011702658 | 0.010673141 | 0.000829297 | -0.001955758 | 0.002367862 |
| PC49 | 0.001465336 | -0.000280884 | 0.003692722 | -0.000480259 | -0.001092428 | -0.001358976 | -0.003556602 | 0.005193224 | -0.000486552 | -0.00868073 | -0.001788088 | -0.004252228 | 0.001509253 | 0.000450889 | -0.005942682 | -0.003730789 | -0.008686848 | -0.000591253 | 0.012651152 | 0.000484724 | 0.004356938 |
| PC50 | -0.01088656 | 0.002593383 | -0.015890071 | 0.002494026 | -0.001605465 | -0.006977371 | -0.00383701 | 0.003889939 | 0.004896339 | 0.001950751 | 0.000673547 | -0.00248413 | 0.007399602 | -0.004255671 | -0.002759434 | -0.001565906 | -0.002553477 | -0.001746791 | -0.000752972 | 0.002157277 | -0.001168633 |
| PC51 | -0.013722334 | 0.004657105 | -0.000748406 | 0.004227796 | 0.012613777 | -0.007353615 | -0.011917551 | 0.002822253 | 0.021980333 | -0.004512488 | 0.002283133 | -0.000287877 | 0.000480808 | 0.004514766 | 0.009950806 | 0.002720073 | -0.004651706 | -0.002751541 | -0.008439956 | -0.005787647 | -0.002039409 |
| PC52 | -0.024658681 | 0.01968329 | 0.024894275 | -0.029330137 | -0.004266757 | 0.003080744 | -0.005245345 | 0.000643722 | -0.002470755 | -0.001459452 | 0.000749149 | 0.001203356 | -0.003894713 | -0.001761287 | -0.002868739 | -0.000339005 | 0.004188045 | 0.001813849 | 0.004305744 | 0.003064748 | 0.001116738 |
| PC53 | -0.006416421 | 0.007544969 | -0.010204153 | -0.008470978 | -0.006112262 | -0.000537377 | 0.008399711 | -0.008652048 | -0.014310837 | 0.010881672 | 0.003111366 | -0.007577289 | 0.003427499 | 0.001781645 | -0.000298741 | 0.005621692 | 0.007313505 | -0.001433564 | -0.005944576 | 0.001273176 | 0.003007282 |
| PC54 | 0.005364423 | 0.003813439 | 0.016963171 | 0.005567429 | 0.024775353 | -0.00931429 | 0.000141138 | 0.006950083 | -0.005749316 | -0.001860574 | -0.001340863 | 0.010111523 | 1.00E-04 | -0.008052252 | -0.008281219 | -0.001233948 | 0.002274398 | 0.003235589 | 0.006213629 | 0.001467284 | -0.000291429 |
| PC55 | 0.011072833 | 0.003577969 | -0.021746678 | 0.00574171 | -0.016720162 | 0.005504277 | -0.008889248 | 0.003550305 | -0.00208989 | 0.002787594 | -0.00102831 | -0.009652678 | -0.003418273 | 0.005375827 | 0.002309247 | -0.00265906 | -0.0001361 | -0.002148122 | -0.001851147 | 0.000637691 | 0.000526921 |
| PC56 | -0.027609059 | -0.007178772 | -0.015768809 | -0.000860766 | 0.007509383 | 0.0251075 | 0.004121311 | -0.001084808 | -0.009003888 | -0.007059086 | 0.001182776 | 0.011302728 | 0.001935511 | -0.002356923 | 0.003267719 | 0.011880032 | -0.002992829 | 5.80E-05 | 0.001052339 | -0.003690754 | -0.003177452 |
| PC57 | -0.001609246 | 0.012701062 | -0.013528993 | 0.000883307 | 0.012616699 | 0.010206976 | 0.013486623 | -0.006632601 | -0.006590563 | -0.005660601 | 0.009019227 | 0.007146808 | -0.00768191 | 0.012066837 | -0.000521122 | -0.005285553 | -0.002794641 | -0.01010394 | -0.003090258 | 0.006302735 | 0.00444374 |
| PC58 | 0.022499368 | -0.00316555 | 0.026379247 | -0.008617565 | 0.000298489 | -0.017808538 | 0.015235886 | 0.011476974 | -0.002300863 | -0.00216508 | -0.003481151 | -0.001186311 | 0.005436253 | 0.005924614 | -0.005322883 | 0.001206412 | -0.003410719 | 0.00441974 | -0.001128633 | 0.001932383 | 0.002467516 |
| PC59 | -0.008490752 | -0.004463026 | 0.005740693 | 0.007671072 | 0.007654052 | 0.002731705 | 0.00886181 | 0.006126184 | 0.01413177 | 0.005395308 | -0.003351979 | -0.013195857 | 0.00259136 | -0.009231191 | 0.012107038 | -0.010278878 | 0.010525503 | 0.001038923 | 0.002120172 | -9.60E-05 | -0.003571199 |
| PC60 | -0.004495797 | -0.024933029 | -0.022342673 | -0.010449564 | 0.014970772 | -0.000874943 | -0.012065448 | -0.007952094 | -0.000115116 | -0.004161017 | -0.006148184 | 0.001295044 | -0.002886367 | 0.002833955 | -0.008237376 | 0.010556633 | -0.002853477 | 0.002103613 | 0.000647421 | -0.00222973 | -0.000555381 |
| PC61 | -0.00930406 | -0.003099623 | 0.007653626 | 0.018477937 | -0.005602085 | -0.008788943 | 0.009341844 | -0.007136331 | 0.011950817 | -0.00219069 | 0.001152186 | 0.005229659 | -0.004117083 | 0.001524629 | -0.001764285 | 0.008112517 | 0.009060093 | -0.004973289 | 0.003065951 | -0.003349265 | 0.002009169 |
| PC62 | 0.009454902 | 0.002325041 | 0.003983717 | 0.002701657 | 0.008151704 | 0.004821843 | -0.006379351 | 0.014302143 | -0.003562582 | -0.002552479 | -0.007112561 | 0.00669178 | 0.000214394 | -0.008705624 | 0.000568842 | -0.002255119 | -0.003785633 | 0.002224961 | -0.00428692 | 0.000179628 | 0.001980137 |
| PC63 | 0.01046715 | -0.004112531 | 0.001368501 | -0.00693647 | -0.001731383 | 0.006907561 | -0.000423913 | -0.003306947 | -0.006191796 | 0.002854852 | 0.004203517 | -0.007244105 | 0.001120007 | 0.001200249 | 0.00237695 | -0.005173673 | -0.004454072 | 0.000636704 | -0.000321102 | 0.00271338 | -0.009399333 |

|  | 2968 | 2983 | 2984 | 2985 | 3204 | 3205 | 3220 | 3221 | 3222 | 3223 | 3237 | 3238 | 3239 | 3240 | 3241 | 3256 | 3257 | 3258 | 3492 | 3493 | 3494 |
| --- | --- | --- | --- | --- | --- | --- | --- | --- | --- | --- | --- | --- | --- | --- | --- | --- | --- | --- | --- | --- | --- |
| PC64 | 0.001772339 | -0.016823037 | -0.003274793 | 0.00774927 | -0.005838874 | -0.006542539 | -0.00130554 | -0.003738644 | 0.011387516 | -0.007642016 | 0.011618986 | -0.006841155 | -0.004173981 | 0.0173046 | 0.003922797 | 0.000882596 | -0.001276541 | 0.000696148 | 0.005758348 | -0.000498839 | 0.00516512 |
| PC65 | 0.009187621 | -0.013078951 | -0.018932984 | 0.020520489 | 0.003073144 | -0.007482793 | 0.018197425 | -0.002863725 | -0.000567499 | 0.005386229 | -0.011445383 | 0.01053875 | 0.006369506 | -0.009274913 | -0.006021961 | -0.00135298 | 0.000248208 | 0.001129816 | -0.005321942 | -0.000498812 | -0.000406667 |
| PC66 | 0.015468586 | 0.001673112 | -0.006122659 | 0.002552192 | 0.008912314 | 0.001381208 | -0.006613038 | 0.010062184 | 0.001310809 | -0.004080611 | 0.002974668 | 0.006180361 | -0.008132406 | 0.008429905 | 0.002996154 | -0.004561833 | 0.001841735 | 0.00162858 | 0.003621404 | 0.001281639 | -0.002949744 |
| PC67 | -0.010798898 | 0.000627298 | 0.002474269 | -0.030027309 | -0.014507372 | 0.003493235 | -0.005003678 | -0.008509802 | 0.005438531 | -0.007982504 | -0.007530589 | -0.005453565 | 0.00255622 | -0.009078169 | -0.003695082 | 0.003020737 | -0.002969288 | -0.004086074 | -0.005466164 | -0.003209591 | 0.00154393 |
| PC68 | -0.01459177 | 0.004767586 | 0.013149072 | -0.001943854 | 0.003228095 | -0.000414758 | 0.008719936 | 0.004659266 | 0.003623691 | 0.006508845 | 0.005860162 | 7.60E-05 | 0.002561909 | 0.004357633 | 0.009025646 | 0.003268817 | 0.001796047 | -0.001435093 | 0.004490606 | 0.002672698 | -0.000972165 |
| PC69 | 0.002655579 | -0.012120311 | 0.010411596 | 0.010893432 | -0.000637925 | 0.004441432 | -0.024337105 | -0.01177587 | 0.001776018 | -0.00442782 | 0.008037478 | -0.001118323 | -0.008023462 | -0.008295225 | -0.010311588 | -0.009062778 | 0.00026722 | 0.00185961 | -0.003364511 | -0.004568694 | 0.000352462 |
| PC70 | 0.023869204 | 0.040758911 | -0.005090098 | 0.001341394 | -0.014193358 | -0.013460657 | -0.003147139 | 0.00299817 | -0.003504917 | 0.005759233 | -0.015259777 | 0.002793245 | 0.002412323 | 0.00286299 | 0.008169056 | 0.008244215 | -0.003683873 | 4.71E-05 | 0.003370173 | 0.003485143 | 9.88E-05 |
| PC71 | -0.008806213 | 0.00700247 | -0.006868693 | 0.013819076 | -0.00169734 | -0.002676638 | -0.007861226 | 0.00416087 | -0.007504258 | -0.001185692 | 5.49E-05 | 0.001766801 | -0.001415145 | -0.0009576 | -0.001482996 | -0.000922436 | -0.000834258 | -0.000222948 | -0.000706006 | 0.000336129 | -0.000549264 |
| PC72 | 0.025132777 | -0.000793707 | -0.017740772 | -0.020870291 | 0.004508905 | 0.012592 | 0.008254274 | -0.003886205 | 0.017213428 | 0.005670945 | -0.002669653 | 0.001519326 | 0.005792169 | -0.000405553 | 0.003095322 | 0.005276035 | 0.000800748 | 0.001079332 | 0.002989909 | -7.19E-05 | -1.23E-05 |
| PC73 | -0.0348752 | -0.006974175 | 0.017422717 | 0.005685199 | -0.00771389 | -0.005534532 | 0.01353073 | 0.00743109 | -0.01915288 | -0.00600876 | 0.005932896 | -0.001330785 | -0.001361408 | 0.004704505 | -0.005299074 | -0.002214666 | -0.000152531 | -0.001802413 | -0.003028977 | 0.000402345 | 0.000568765 |
| PC74 | 0.001290461 | 0.002331993 | -3.87E-05 | 0.005071909 | 0.003819052 | 0.0059298 | -0.002544006 | -0.002953472 | 0.004918818 | 0.003627196 | 0.003286903 | 0.001994578 | 0.000750626 | -0.002362012 | 0.003505351 | -0.001241934 | 0.000316657 | 0.000132902 | 0.000198379 | 0.000289642 | -0.000407433 |
| PC75 | -0.010957318 | 0.00206095 | -0.016387577 | -0.013009714 | -0.005850543 | 0.00298687 | -0.009561528 | -0.002166464 | -0.002842487 | 0.005952026 | 0.001673475 | -0.0052358 | 0.001282306 | 0.001748846 | -0.000164884 | -0.007453523 | -0.003037587 | 0.006360269 | 0.000583315 | 0.003491267 | -0.001606172 |
| PC76 | 0.001647151 | 0.003197548 | 0.008450026 | -0.005237569 | 0.00890795 | 0.000261821 | 0.008227084 | -0.006801464 | -0.006459326 | 0.002944557 | 0.000132264 | -0.002062857 | 0.007989765 | 0.002045905 | -0.001180937 | 0.002293947 | -0.004977019 | -0.0002299 | -0.004361716 | -0.001285894 | 0.002009739 |
| PC77 | 0.003913089 | -0.000578597 | 0.005823648 | 0.008619113 | -0.006095364 | -0.000355896 | 0.004566934 | -0.003161476 | -0.009550144 | 0.00461622 | -0.000894943 | -0.007890779 | -0.000387708 | -0.012430956 | -0.00243583 | 0.008618374 | 0.0105832 | -0.00633974 | 0.00093494 | 0.000239733 | 0.00368286 |
| PC78 | -0.016528516 | 0.012459627 | -0.013873534 | -0.001693346 | -0.004678016 | 0.002004486 | -0.003356891 | 0.011616136 | -0.00318382 | 0.00130409 | -0.010409453 | 0.013203701 | 0.000257391 | 0.000811844 | 0.002299439 | -0.006635116 | -0.005116973 | 0.004620509 | -0.002794245 | 7.66E-05 | -0.003551533 |
| PC79 | 0.020358904 | 0.008270589 | 0.006642752 | -0.000882185 | 0.014568872 | 0.005581595 | -0.010219151 | -0.002082864 | -0.001846959 | -0.002096552 | 0.008353819 | -0.007400326 | -0.000953636 | 0.00238541 | -0.00076945 | 0.002877426 | -0.000498062 | -0.002123858 | 0.002364338 | -0.000243539 | 0.000370223 |
| PC80 | -0.008428417 | -0.003076736 | 0.001417236 | 0.011161228 | -0.008585331 | -0.005031894 | 0.002706775 | 0.009356528 | 0.015349079 | -0.012361649 | -0.004325015 | 0.009458964 | -0.005235855 | -0.000638939 | 0.000517947 | 0.00058946 | -0.000111232 | -0.003969044 | 0.00276164 | -0.002796798 | 0.000120142 |
| PC81 | 0.010475347 | -0.007790521 | 0.00371885 | 0.010480933 | 0.017115076 | 0.003588341 | -0.00060774 | -0.016386977 | -0.007962626 | 0.016047733 | 0.008472193 | -0.004083951 | 0.00597831 | 0.00287899 | -0.001829564 | 0.002003004 | -0.000309429 | 0.006215576 | -0.00043314 | 0.001473956 | -0.000234205 |
| PC82 | -0.000445702 | -0.007337642 | -0.008348327 | -0.018273572 | -0.019173142 | -0.005357652 | 0.014227076 | 0.00539527 | 0.002055068 | -0.010851682 | -0.006405779 | -0.002494121 | -0.005140525 | -0.000339531 | -8.86E-05 | -0.005121782 | 0.003108901 | -0.003056052 | -0.000285778 | -0.000948403 | 0.001441077 |
| PC83 | -0.019307083 | 0.005419935 | 0.001721679 | 0.023410438 | 0.008130684 | -0.009848801 | -0.014045993 | -0.001505852 | -0.008822617 | 0.006509713 | -0.000390585 | 0.000650187 | 0.001451588 | 0.001769486 | 0.003700164 | 0.003081149 | -0.000908513 | 0.001450093 | -6.88E-05 | 0.002814188 | -0.001432399 |
| PC84 | 0.01169841 | -0.017029789 | -0.006615077 | -0.017970381 | 0.002176821 | 0.005367584 | 0.005067288 | 0.002252165 | 0.008996747 | -0.00406257 | 0.002218011 | -0.001037782 | 0.000143488 | -0.001686981 | -0.003785334 | -0.002953866 | 0.00129233 | -0.000185368 | 7.43E-05 | -0.003142479 | 0.000725463 |
| PC85 | -0.000485139 | -0.000719459 | 0.003343463 | 0.004322663 | 0.005772214 | 0.002657242 | -0.005184003 | -0.000290522 | 0.000206047 | 0.000364584 | 0.000242951 | 0.000529928 | 0.000401962 | 0.00022707 | 0.000876688 | 0.001837534 | -0.001373799 | -3.95E-05 | -0.000802119 | -0.000789219 | -0.0001835 |

Color number value 3495-3801:

|  | 3495 | 3496 | 3510 | 3511 | 3512 | 3513 | 3514 | 3528 | 3529 | 3530 | 3531 | 3765 | 3766 | 3767 | 3768 | 3769 | 3783 | 3784 | 3785 | 3786 | 3787 | 3801 |
| --- | --- | --- | --- | --- | --- | --- | --- | --- | --- | --- | --- | --- | --- | --- | --- | --- | --- | --- | --- | --- | --- | --- |
| PC1 | 0.000136722 | 0.000803826 | 0.000945427 | -0.002408261 | 0.001103988 | -0.000239376 | -0.000628099 | 1.21E-05 | -0.000375429 | 0.000490851 | -0.000321706 | 0.000194983 | -0.000119287 | -0.000115219 | 0.000707095 | 4.70E-05 | 0.000242687 | -0.000267202 | 3.36E-05 | -0.000200274 | -3.96E-06 | -3.54E-05 |
| PC2 | -0.003915438 | -0.000960306 | -0.001915583 | 0.003648587 | -0.001314854 | 2.54E-05 | -0.001920746 | -0.001781416 | -0.001044338 | -1.64E-06 | 0.000503651 | -4.66E-05 | 0.000186567 | 0.001052637 | -0.002200835 | -0.000719807 | -0.001538053 | 0.000493539 | 0.000140304 | 0.000439932 | -9.38E-05 | -8.76E-06 |
| PC3 | -0.000823843 | -0.003936749 | 0.000830763 | 0.000663565 | 0.000969569 | 0.000707137 | 0.000325778 | 0.001308421 | 0.003320857 | -0.000750711 | 0.000307127 | -0.000301133 | 0.002352882 | -0.002554584 | -3.93E-05 | 0.001565692 | 0.00161541 | -9.90E-05 | -0.000856069 | 0.000229776 | 0.00019355 | -0.000139172 |
| PC4 | -0.006250773 | 0.004733139 | -0.001145806 | -0.004311486 | 0.002318194 | -0.000936056 | 0.0018063 | 0.000452158 | -0.001452188 | -0.002423432 | -0.001447879 | -0.000819531 | -0.000691979 | 0.000805981 | 3.88E-05 | -0.000754216 | -0.001182694 | 0.000412248 | 0.000636389 | 0.000156974 | -0.000228522 | 0.000238967 |
| PC5 | 0.001154258 | 0.005834646 | 0.000427072 | -0.003149003 | 0.000778003 | 0.000389505 | 0.00078874 | 0.001487925 | 0.000361762 | -0.001140319 | -0.000221723 | 0.000161978 | -0.000670876 | -0.000865245 | 0.001182642 | 0.000384053 | 0.001395824 | -8.54E-05 | -0.000616007 | -1.76E-05 | 1.31E-06 | 1.23E-05 |
| PC6 | 0.001909177 | -0.005798682 | -0.000827276 | 0.000763527 | -0.002091067 | -0.000465864 | -0.000705347 | -0.000483738 | 0.000555121 | -0.000271713 | -0.001502663 | 0.00103197 | -5.28E-05 | 0.000999379 | 0.001908635 | -0.000914781 | -0.000626691 | -0.001132296 | 0.00147954 | -0.001121249 | 0.000365141 | -0.000101118 |
| PC7 | 0.003548113 | -0.004070538 | 0.003181136 | 0.004694646 | -0.002383611 | 0.000856348 | 0.000155471 | -0.00026611 | 0.001144132 | 0.001016715 | 0.002816643 | -0.001887281 | 0.000259212 | 0.000127006 | -0.000549931 | 0.000230558 | -0.000212439 | 0.000646031 | 0.000228775 | 0.000257406 | 0.000382885 | 2.69E-05 |
| PC8 | 0.001158392 | -0.003423461 | -0.000149016 | 0.000830474 | -0.000367232 | -0.001292862 | -0.00147982 | -0.000749785 | -0.000933805 | 0.001833622 | -0.000578419 | 0.002341389 | -0.000447125 | 0.002098311 | -0.000805303 | -0.000149996 | 0.000129827 | -0.000552085 | -0.001264305 | 0.00042301 | -0.001299147 | 0.000229727 |
| PC9 | 0.005502311 | 0.004956276 | -0.000538244 | 0.000802597 | 0.000322349 | 0.000695187 | 0.002490185 | 0.000991019 | -0.002768216 | 0.003201245 | 0.0031124 | -0.000223376 | -0.003597365 | 0.000708934 | -0.000357375 | 0.000374462 | 0.000210476 | 0.001120967 | -0.000123873 | -3.01E-05 | 0.000693325 | -2.10E-05 |
| PC10 | -0.000784989 | 0.001783555 | 0.00174876 | 0.003101748 | -0.002256975 | 0.001610876 | -3.83E-05 | 0.000595738 | 0.000513974 | -0.001542938 | 0.000538095 | 0.000280289 | -0.001329904 | -0.000590699 | 0.000100327 | 0.000406222 | -0.000143609 | 0.000107925 | -0.000121873 | 0.000122084 | -0.000101451 | 1.99E-05 |
| PC11 | 0.000931605 | -0.007846833 | -0.00277803 | -0.002331424 | 0.001735773 | -0.003003814 | 0.0030147 | 0.000650626 | 0.000525337 | 0.001875022 | -8.31E-05 | -0.000404646 | -0.000504735 | -0.000415778 | -0.000629675 | 0.000841109 | -0.00021684 | -3.89E-05 | 0.000284832 | -0.000194633 | -0.000333617 | 0.000138097 |
| PC12 | -0.00353813 | 0.006316058 | -0.001122671 | -0.000827122 | 0.001143251 | 0.000697411 | -0.004466299 | -0.002961674 | -0.002411342 | 0.000426749 | -0.0019456 | 0.000702119 | 0.002140334 | 0.000936896 | -0.000786463 | -0.000583719 | 0.00045646 | -0.000106648 | -0.000310145 | 0.000318559 | 0.000796986 | -0.000598536 |
| PC13 | 0.004574123 | 0.008306459 | 0.002494143 | -0.002492732 | -0.000233185 | 0.000410722 | 0.001515499 | -0.002953023 | 0.002026039 | -4.25E-05 | -0.00042672 | -0.003734889 | 0.001855096 | -0.002157901 | -0.000229313 | -0.001115437 | 0.000104686 | -0.000270861 | 0.000602666 | 0.000115834 | -0.000606081 | 0.000233133 |
| PC14 | -0.006838567 | 0.002809893 | -0.00491117 | 0.002981391 | 0.002268062 | 0.001217229 | 0.002343906 | 0.002859445 | -0.00151253 | 0.00030736 | -0.001906404 | 0.002750779 | -0.000703171 | -0.001781801 | 0.000945223 | 0.000604665 | 0.000270532 | 3.78E-05 | 2.70E-05 | -0.000491559 | -5.88E-05 | 0.000172465 |
| PC15 | -0.004157866 | -0.008304808 | 0.001968361 | -0.00675943 | 0.0032831 | -0.001681951 | 0.00023093 | 0.000945547 | 0.002375112 | -0.000957205 | 0.000791685 | -0.000436853 | 0.001685896 | 0.000505006 | -0.000346366 | 0.001602529 | -0.001132252 | 0.000291626 | -3.41E-05 | 0.000154404 | 0.000453889 | -0.000194391 |
| PC16 | 0.00344787 | -0.000248504 | 0.004650185 | 0.00517882 | -0.001439841 | 0.004328735 | -0.001007748 | -0.000545959 | -0.001008712 | 0.000470071 | 4.47E-05 | 0.000673084 | 0.000883874 | -0.001239123 | 0.001286151 | -0.000400615 | -0.000707529 | -0.000255123 | 2.84E-05 | -0.000191001 | -0.000300399 | 1.29E-05 |
| PC17 | -0.002084087 | 0.000993062 | -0.001123077 | 0.002194577 | 0.000105743 | 0.000374289 | 0.001869707 | -0.001391951 | 0.000726313 | -0.001831723 | 0.001253981 | -0.001099436 | -0.000478251 | -0.000582462 | 0.001564755 | 0.000138797 | -0.000585918 | 0.000133507 | -0.000277731 | -0.000141787 | -0.00031974 | -0.000748118 |
| PC18 | -0.002014804 | -0.001105828 | 0.000527013 | 0.001472343 | 0.002763704 | 0.000475082 | -0.001448318 | 0.001081854 | 0.000232568 | 0.000729079 | 0.000738409 | 0.001121888 | 0.00078273 | 0.000442029 | 0.000805773 | 6.87E-06 | 0.000879166 | 0.000384854 | 0.000729613 | 0.000583031 | 0.000230633 | 0.001379767 |
| PC19 | 0.001353072 | -0.002431533 | -0.001300537 | 0.001839852 | -0.001805829 | -0.000702357 | 0.000979116 | 0.000321704 | 1.00E-05 | -0.000513179 | -3.33E-05 | 0.001350549 | -0.001228219 | -0.000566948 | -0.0008058 | 0.000415775 | -0.000613031 | -2.11E-05 | -0.000646438 | -0.000451386 | -3.97E-05 | -0.00044006 |
| PC20 | 0.007049115 | -0.001422152 | 0.001316356 | -0.000240643 | 0.001095012 | 0.000139276 | -0.000274198 | 0.001694952 | -0.0036086 | -0.00026857 | -0.000957693 | 1.32E-05 | 0.001962314 | -0.000776387 | -0.00219362 | -0.001466127 | 0.000293796 | -0.00032971 | 4.59E-05 | -0.000283704 | 0.000164183 | 8.03E-06 |
| PC21 | -0.003997223 | 0.004365246 | 0.00711481 | -0.000567949 | -9.39E-05 | 0.000567449 | -0.000772613 | 0.000407268 | 0.002559071 | -0.000490209 | 0.000684917 | -0.000379378 | -0.00032769 | 0.003254367 | 0.001982748 | 0.000959822 | -0.000625583 | -0.000259354 | -0.00028148 | 0.000167777 | -5.45E-05 | -7.84E-05 |

|  | 3495 | 3496 | 3510 | 3511 | 3512 | 3513 | 3514 | 3528 | 3529 | 3530 | 3531 | 3765 | 3766 | 3767 | 3768 | 3769 | 3783 | 3784 | 3785 | 3786 | 3787 | 3801 |
| --- | --- | --- | --- | --- | --- | --- | --- | --- | --- | --- | --- | --- | --- | --- | --- | --- | --- | --- | --- | --- | --- | --- |
| PC22 | -0.001800532 | -0.001969557 | -0.001464782 | -0.003432989 | -0.001770834 | -0.002132608 | -0.004164035 | -0.00050022 | 0.00143491 | -0.002661136 | 0.002444324 | -0.000418759 | -0.003108692 | -0.001684825 | -0.001779736 | -0.00159732 | 0.000636551 | -0.00039916 | 0.000243149 | -0.000280885 | -0.000108951 | 0.000126282 |
| PC23 | 0.008155217 | -0.000680041 | -0.003914017 | 0.001256975 | -0.002391409 | -0.004504751 | 0.000232345 | 0.001551159 | 0.000743313 | -0.004060289 | -0.002727717 | 0.000492694 | 0.000349135 | 0.002223417 | 8.22E-05 | 0.000570201 | 0.00109324 | 0.000406276 | 6.84E-05 | 0.000525893 | 0.000234906 | -2.92E-05 |
| PC24 | 0.000613537 | 0.000111497 | -0.000389018 | 0.001542939 | 0.002606303 | 0.002792512 | 0.002078128 | 0.002308954 | -0.001070165 | -0.000708406 | -0.001124663 | 0.000399985 | 0.001154851 | -0.000677068 | -0.000405325 | 0.00010364 | -0.001428055 | 0.000516316 | -2.52E-05 | 0.000272776 | -6.99E-05 | -3.85E-05 |
| PC25 | -0.008531283 | -0.002004603 | -0.000263546 | 0.003357895 | -0.001195429 | 0.000209919 | 0.000320689 | -0.00142574 | -0.000300751 | 0.003566101 | 0.000946716 | -0.001250217 | -0.000234829 | 0.001266487 | 0.000486355 | -0.00067335 | 0.001713118 | -0.000913755 | -7.38E-05 | -0.000398424 | 8.33E-05 | -3.03E-05 |
| PC26 | 0.000789527 | 0.002791685 | 0.003438073 | 0.00163691 | -0.000647265 | -0.00082556 | -0.000389487 | 0.000877031 | 0.001662574 | 0.002032093 | -0.003490034 | -0.00140761 | -0.002221004 | -0.000640303 | -0.001367977 | 0.001563325 | -0.001059764 | -0.001528171 | -0.000269444 | 0.000229166 | 0.00047567 | 0.000312918 |
| PC27 | -0.003367782 | 0.001738001 | 0.003344147 | 0.001568308 | 0.00070843 | -0.001264137 | -0.000865498 | -0.001385426 | 0.001624264 | 0.000920267 | -0.000856688 | 0.001573727 | 0.000539866 | 0.00023176 | -0.000836967 | 0.0007157 | 0.0013415 | 0.001979383 | 0.001150839 | -0.000454602 | -0.000525419 | -0.000386885 |
| PC28 | -0.004082495 | -0.003596304 | -0.004998845 | -0.001117334 | -0.003095449 | 0.001992535 | 0.003368734 | -0.000226016 | -0.004252663 | 0.001163141 | 0.00038355 | -0.002267693 | 0.00178737 | -0.000414438 | 0.001313417 | -0.000251179 | -1.77E-05 | -6.78E-05 | -0.000337573 | 0.000355083 | 2.92E-05 | 0.000104085 |
| PC29 | 0.003129559 | 0.001756013 | -0.006453363 | 0.001294508 | 0.002478518 | 0.000322498 | -0.002341522 | 0.00144913 | 0.002616657 | -0.000961331 | 0.002438554 | 0.002047353 | 0.000142586 | -0.001101037 | 0.000550441 | -0.001233674 | -0.000531823 | -0.000243364 | -0.000148557 | 0.00014463 | 8.66E-05 | -3.55E-06 |
| PC30 | 0.006730223 | -0.0017664 | 0.005450743 | -0.002371826 | 5.18E-05 | 0.000928286 | 0.004778922 | -0.002147218 | -0.000847355 | -0.000264073 | -0.001355675 | 8.74E-05 | -0.001334063 | 0.000830462 | -0.000223762 | -0.000187039 | 0.000668583 | 0.000103906 | -2.08E-05 | -0.000155138 | 9.69E-06 | -5.16E-06 |
| PC31 | 0.00471937 | 0.003307967 | -0.000862782 | -0.002509174 | -0.000674438 | -0.000435755 | -0.002065153 | -0.001386979 | 0.00092873 | 0.003570052 | 0.001042489 | 0.001252667 | 0.00106414 | -0.000995574 | 0.000534939 | 0.001121313 | -0.001337733 | 0.000240831 | 3.38E-05 | -0.000125068 | -0.000104592 | 6.07E-05 |
| PC32 | -0.002598087 | 0.001072209 | -0.004085591 | -0.000444555 | -0.004444196 | -0.003840888 | -0.003286039 | -0.001164622 | -0.000179035 | 0.000703793 | 0.000690518 | -0.000751247 | 0.000351253 | -0.000254051 | 0.000426142 | 0.000627995 | 5.16E-05 | 4.84E-05 | 0.000110918 | 1.45E-05 | -8.83E-06 | 5.03E-05 |
| PC33 | -0.000894146 | -0.002606543 | 0.004089697 | 0.002823622 | 0.002583671 | 0.003760601 | 0.000140877 | 0.000101551 | 0.001423583 | -0.001731067 | 0.000189119 | -0.000280074 | -0.000136701 | 0.001136412 | -0.00099354 | -0.001583442 | 0.000787911 | -0.00051195 | -2.77E-05 | 0.000166872 | 7.17E-05 | -0.000125491 |
| PC34 | 0.000428386 | 7.37E-05 | 0.001326503 | -0.002790677 | 0.00097821 | -0.001074222 | -0.001924892 | 0.000765988 | -0.001675933 | -0.000477527 | 9.20E-05 | -0.000127365 | -0.000652858 | -0.000610088 | 0.000898276 | 0.000503947 | -0.00016378 | 0.000204951 | -9.13E-05 | -1.18E-05 | -7.09E-05 | -8.37E-06 |
| PC35 | 0.000918201 | -0.00143871 | -0.005059527 | -0.004808143 | 0.001725325 | 0.001666822 | -0.001218754 | 0.002959508 | 0.001286855 | 0.001712831 | -0.000358861 | -0.002193045 | 0.001077705 | 0.001814515 | -4.16E-05 | -0.001620561 | 3.81E-05 | 0.000755601 | -0.000912832 | -0.001048294 | -3.69E-05 | 0.000121065 |
| PC36 | 0.002987811 | -0.000923868 | 0.000524255 | -0.001399477 | 0.000852324 | 0.001030542 | 0.000238284 | 0.00075955 | -0.000796881 | -0.001539158 | 0.002758559 | 0.001893379 | 0.000247489 | 0.000600208 | 0.001333871 | -0.001111577 | 2.84E-06 | -0.00078188 | 0.00039441 | 0.000836907 | -2.28E-05 | -0.000224197 |
| PC37 | -0.001942964 | -0.003180369 | -0.002228672 | -0.006104683 | -0.001721435 | 0.000329868 | 0.001337472 | -0.0021662 | -0.000766117 | 0.00207835 | -0.000538968 | 0.001064078 | -0.001399985 | -0.001189554 | -0.000112421 | 0.000689898 | 0.000585455 | -0.000387339 | 0.000638083 | 0.000889791 | 8.27E-06 | -0.00035254 |
| PC38 | -0.002495986 | 0.000923331 | 0.003860848 | 0.000993622 | -0.003709144 | -0.001742786 | 0.000930328 | -0.004934794 | -0.001276515 | -0.004198797 | 0.000864989 | 0.000969688 | 0.000540803 | -0.000307093 | -0.000133098 | 0.000657539 | -0.00039637 | 0.000330098 | -0.000978914 | -0.000807242 | 0.000317832 | 0.000698504 |
| PC39 | -0.004592646 | 0.001142213 | -0.000992813 | 0.00606902 | 0.000739791 | 0.000675889 | -0.003928944 | 0.00254354 | 0.000650861 | 0.000891538 | -0.001570232 | -0.001971651 | -0.001110408 | -0.000291673 | -0.000176806 | 0.000391079 | 1.48E-05 | 0.000192578 | 0.000199256 | 0.000182777 | -0.000120009 | -0.000143846 |
| PC40 | 0.001224858 | -0.002427624 | 0.004035132 | -0.000847456 | -0.001430692 | -0.000952209 | 0.001242238 | -0.000289195 | -0.001805464 | -6.76E-05 | -8.50E-06 | 0.0002121 | 0.000211838 | -8.45E-05 | 0.000103139 | -0.000215491 | 0.000102514 | 0.000137903 | 0.000226851 | 0.000140006 | -0.000143069 | -0.000162748 |
| PC41 | -0.001431968 | 0.001234878 | -0.001474553 | -0.000508964 | 0.000470726 | 0.001709723 | 0.000285291 | 0.000277723 | 0.00102884 | 0.000314447 | -3.45E-05 | 7.54E-05 | 6.12E-05 | 0.000220561 | -0.000243731 | 0.000362145 | 6.86E-05 | -0.00018442 | -3.40E-05 | -0.000120656 | 7.62E-05 | 5.42E-05 |
| PC42 | -0.000244386 | -0.002850921 | 0.003221213 | 0.003240176 | -9.59E-05 | -0.002703863 | -0.000418022 | 0.001681334 | -0.002028128 | -3.92E-05 | -3.25E-05 | -0.000918747 | 0.000864087 | -0.000411459 | 0.000636583 | -0.000352821 | -8.41E-05 | 0.000288188 | 1.58E-05 | 0.000192531 | -9.77E-05 | -5.08E-05 |

|  | 3495 | 3496 | 3510 | 3511 | 3512 | 3513 | 3514 | 3528 | 3529 | 3530 | 3531 | 3765 | 3766 | 3767 | 3768 | 3769 | 3783 | 3784 | 3785 | 3786 | 3787 | 3801 |
| --- | --- | --- | --- | --- | --- | --- | --- | --- | --- | --- | --- | --- | --- | --- | --- | --- | --- | --- | --- | --- | --- | --- |
| PC43 | 0.001070995 | 0.003974017 | -0.006341115 | -5.56E-05 | -0.002041215 | 0.000555245 | 0.002577996 | -0.000163019 | 0.000931286 | -0.001892508 | -0.00051956 | -0.000234728 | -0.000206716 | 0.000428876 | -0.000183108 | 2.21E-05 | 0.000153502 | -3.11E-05 | -4.39E-07 | 7.18E-05 | 5.61E-05 | 1.33E-05 |
| PC44 | -0.001065692 | -0.000304322 | 0.001105608 | -0.001175461 | 0.002384486 | 0.002432617 | 0.002051398 | -7.53E-05 | 0.000544289 | -0.000637279 | -0.00028847 | 0.000452873 | -0.000757828 | -0.000134748 | -0.000457442 | 1.23E-05 | -2.11E-05 | -1.97E-05 | 1.05E-05 | -9.00E-05 | 3.17E-05 | 1.30E-05 |
| PC45 | 0.002432848 | 0.001638947 | 0.002266127 | -0.003589565 | 0.002082037 | -0.002927265 | -0.004141834 | -0.000634026 | -0.001853688 | 0.001217692 | -4.66E-05 | -0.000129101 | -0.00020145 | -0.000158057 | 0.000923141 | 0.000733017 | 6.65E-05 | 8.19E-05 | -0.000131983 | -9.86E-05 | -5.86E-05 | 1.20E-05 |
| PC46 | -0.001709096 | -0.000302769 | -0.00357133 | 0.004450949 | -0.001675363 | 0.001129892 | 0.002668071 | -0.000672126 | 0.00234252 | 0.00036705 | -0.000160212 | -2.02E-07 | 0.000601673 | 0.000453768 | -0.00089315 | -0.000189313 | -0.000160447 | 9.62E-05 | 0.000174022 | 0.000152677 | 4.05E-05 | 5.12E-05 |
| PC47 | -0.001311261 | -0.000492184 | -0.002263328 | 0.002560699 | -0.000214195 | -0.000957047 | 0.001106379 | -0.000327572 | -0.00105713 | -0.001400205 | 0.000639001 | -0.000986015 | -0.000302688 | 0.000162733 | 1.37E-05 | 0.000922357 | 0.000181004 | -0.000102076 | 0.000184361 | -6.50E-06 | -0.000169369 | 1.16E-05 |
| PC48 | 0.007241371 | 0.001998447 | 0.001983942 | 0.001052776 | 0.001231271 | 0.000224875 | -0.000672875 | 0.002911864 | 0.001919545 | 0.001287608 | -0.000575812 | -0.000156571 | -0.000347134 | 0.000439079 | 0.000123075 | -0.000346721 | -0.000192135 | 0.000136742 | 7.77E-05 | -7.07E-05 | -0.000102374 | -9.48E-05 |
| PC49 | -0.001390562 | -0.001060912 | 0.001107513 | 0.000489828 | 0.00209595 | 0.000873341 | -0.000340536 | -0.00302944 | 0.001358375 | 0.001226966 | -0.001156124 | 0.001852319 | 0.000271562 | -0.000952029 | 0.000507698 | -0.001841515 | -0.000252347 | 0.000467473 | -0.000414687 | 0.000133171 | 0.000343934 | 3.40E-05 |
| PC50 | -0.001203459 | 0.00445644 | -0.001027805 | -0.000398421 | 0.001324752 | 0.000145858 | -5.44E-05 | 0.002565622 | -0.002093198 | -0.001440765 | 0.0027552 | -0.000264529 | 0.00170672 | 0.001156329 | -0.001656825 | 0.002165755 | 0.000224095 | -0.001099836 | 0.000522349 | -0.00043104 | -0.000229126 | -6.06E-05 |
| PC51 | -0.001051744 | -0.002483503 | -0.000404834 | 8.94E-05 | -0.00424305 | -0.003184945 | 0.000535301 | 0.001604089 | 0.001110818 | 0.00026194 | -0.001099744 | -0.000671011 | -0.000467222 | -0.000388118 | 0.001136738 | -0.001539141 | -0.000286418 | 0.000488882 | -0.000284395 | 0.000290543 | 6.80E-05 | 6.27E-05 |
| PC52 | 0.000945799 | 0.000384604 | -0.00022381 | -0.000268329 | 0.002206878 | 0.001422168 | 5.74E-06 | -0.001841093 | -0.000270246 | -3.86E-05 | 0.000392059 | 0.000605541 | 0.000110783 | 0.000234847 | -0.000317278 | 0.0005665 | 0.000134255 | -0.000104656 | 9.69E-05 | -9.07E-05 | -1.30E-05 | 9.29E-06 |
| PC53 | 0.003495484 | -0.002322116 | -0.003056922 | -0.00080904 | 0.001356736 | -0.000629468 | -0.002045127 | -0.001391871 | -6.73E-06 | -0.00044886 | -0.000713601 | -0.000857426 | -0.000996724 | -0.000272044 | 0.000449505 | -5.71E-06 | -8.81E-05 | 0.000172339 | -0.000114048 | 6.70E-05 | 4.44E-06 | 2.85E-05 |
| PC54 | -0.00120255 | 0.001191441 | 0.002624922 | -0.000101525 | 0.000904387 | 0.001100888 | 0.001540337 | 0.000160439 | 0.000457635 | 0.000878368 | 0.000532634 | 0.000635483 | 0.000613166 | 0.00050209 | -0.000334636 | 9.60E-06 | 3.49E-05 | -0.000107693 | -9.57E-06 | -0.000101227 | 1.78E-05 | 1.76E-05 |
| PC55 | 0.000672778 | 0.000448524 | -0.000989833 | 0.000200101 | -0.000250151 | -0.001539603 | -0.00142371 | 0.000851186 | -0.00077669 | -0.000679016 | -3.73E-05 | -0.000707172 | -0.000364275 | -0.000189815 | 0.00034362 | -0.000333561 | 3.06E-06 | 4.16E-05 | -5.28E-05 | 8.25E-05 | -4.35E-05 | -6.20E-05 |
| PC56 | 0.000716271 | 0.000446623 | -0.003320676 | -6.59E-05 | -0.002330774 | 0.001626746 | 0.001984508 | -0.001905018 | 0.002728671 | 0.001117281 | 7.52E-05 | 0.001533055 | 0.000704688 | 0.000543086 | -0.000585337 | 0.000346556 | -0.000231989 | -0.000115732 | 9.39E-05 | -8.05E-05 | 5.66E-05 | 0.000113467 |
| PC57 | -0.004901799 | -0.002728893 | 0.005907641 | -0.000103473 | 0.001698203 | 0.000168215 | -0.001279266 | 0.003133345 | -0.00156149 | -0.00047607 | 0.000328292 | 0.000471578 | -0.000568398 | -0.001288419 | -0.000556437 | -0.000531011 | -9.97E-05 | 1.36E-05 | -4.55E-05 | -0.000123918 | 6.04E-05 | -6.29E-05 |
| PC58 | 0.000731758 | -1.45E-05 | 0.001203705 | 0.002720818 | -0.001930321 | -0.001743993 | -0.000470842 | 0.001195304 | -0.000837062 | 8.83E-05 | -0.000168549 | -0.000219814 | 0.000729096 | -0.000231939 | 0.000260673 | 0.000138439 | 7.47E-05 | 9.55E-06 | 6.04E-05 | -1.28E-05 | 8.96E-06 | -3.32E-05 |
| PC59 | -0.002832416 | 0.001181177 | -0.002406387 | -0.003600358 | 0.00310342 | 0.003055194 | 0.000907776 | -0.001395993 | 0.000511609 | -0.000417018 | 0.000224434 | -0.000880478 | -0.00105625 | 0.000719813 | 1.70E-05 | -0.000656787 | -4.03E-05 | -3.28E-05 | -0.000106712 | 0.000148516 | -6.41E-05 | 3.50E-06 |
| PC60 | 0.003596518 | -0.001451594 | 0.001888478 | -0.000462412 | -0.000905606 | -0.002127628 | -6.00E-05 | -0.000145957 | -0.000655841 | 0.00038948 | -6.51E-06 | 0.000113882 | 0.000593481 | 0.000116454 | 0.000209447 | 0.0003793 | 0.00013123 | -9.20E-05 | 1.44E-05 | -0.000112247 | -1.87E-05 | -2.63E-06 |
| PC61 | 0.000205446 | 0.00016444 | -0.001063062 | 0.001225735 | 0.003030199 | -0.004005322 | 0.000653526 | -0.000462337 | 0.000122232 | 8.69E-05 | 0.00074885 | -0.000303655 | 0.000509913 | 0.000315694 | 1.38E-05 | -2.08E-05 | -0.000192055 | -0.000158969 | -3.18E-05 | -7.48E-05 | -2.80E-05 | 3.49E-05 |
| PC62 | -0.000677099 | -0.003187763 | 0.001324263 | -0.003390172 | -0.005176453 | 0.007508519 | -0.002008859 | 0.000938695 | -0.000397617 | -0.000641788 | -0.00117042 | -0.000359131 | -0.000960986 | 4.73E-06 | 9.79E-05 | 0.000615657 | 0.000279976 | 0.000423937 | 7.89E-05 | 6.16E-05 | -9.43E-05 | 2.40E-05 |
| PC63 | -0.002092141 | 0.004013134 | 0.001216787 | 0.002272901 | 0.002354138 | -0.00687618 | 0.003985664 | 5.42E-06 | 0.00059489 | 0.001506968 | 0.000564857 | 0.000719118 | 0.000285682 | -0.000435685 | -0.000264805 | -0.001174653 | -5.46E-06 | -0.000375338 | -0.000103054 | 8.11E-05 | 0.000137109 | -0.000151687 |

|  | 3495 | 3496 | 3510 | 3511 | 3512 | 3513 | 3514 | 3528 | 3529 | 3530 | 3531 | 3765 | 3766 | 3767 | 3768 | 3769 | 3783 | 3784 | 3785 | 3786 | 3787 | 3801 |
| --- | --- | --- | --- | --- | --- | --- | --- | --- | --- | --- | --- | --- | --- | --- | --- | --- | --- | --- | --- | --- | --- | --- |
| PC64 | 0.004054571 | -0.000556158 | -0.00394666 | 0.000131857 | -0.001396249 | 0.004478992 | -0.002027938 | -0.000344228 | -0.000752231 | -0.000755878 | 0.000137133 | 9.04E-05 | 0.000414338 | 0.000109324 | -9.99E-05 | 0.001148043 | 2.79E-05 | -2.56E-05 | 0.000202349 | -0.000104796 | -5.96E-05 | 4.81E-05 |
| PC65 | -0.002038045 | -0.001637213 | 0.002649409 | -0.001214875 | 0.001292023 | -0.000910926 | 0.000587066 | -0.000275631 | 3.58E-05 | 0.000195634 | -0.000119511 | -0.000433132 | -0.0001571 | 2.63E-05 | 0.000201193 | -0.000222191 | -4.08E-05 | 5.35E-05 | -0.000114121 | -4.79E-06 | 1.30E-05 | 1.46E-05 |
| PC66 | 0.001052795 | 0.001222616 | -0.000767674 | 0.001879895 | -0.000661629 | -2.57E-05 | 0.000912461 | 0.000779205 | 0.00014096 | 0.000317576 | 0.000325658 | -0.000354967 | 9.92E-05 | 0.000431803 | 0.000154858 | 4.24E-05 | 2.55E-05 | -7.64E-05 | -1.48E-05 | -3.15E-05 | -2.21E-05 | 2.19E-05 |
| PC67 | -0.00375094 | -0.000186825 | 0.00190026 | -0.000525618 | -0.000548167 | -0.000625538 | -9.72E-05 | -0.000286844 | -6.30E-05 | -0.000844612 | -0.00056516 | 0.000396354 | -9.66E-05 | -0.000637317 | -0.00040015 | 0.000127046 | -8.90E-05 | 7.66E-05 | 0.000102571 | 5.66E-05 | 1.24E-05 | 2.14E-05 |
| PC68 | 0.000613137 | 0.000184908 | -0.001426775 | -0.000263891 | -0.000950597 | 0.000392007 | 2.73E-05 | -4.12E-05 | 0.000381371 | 0.001024944 | -4.13E-05 | 0.000246092 | 0.000142558 | 5.42E-05 | -0.000150551 | 8.30E-06 | 6.39E-05 | -1.17E-05 | -1.29E-05 | -5.01E-06 | 2.88E-05 | 6.02E-06 |
| PC69 | 0.000324395 | -0.000501494 | 0.000676362 | -6.17E-05 | 0.00243235 | -6.98E-05 | -0.00039281 | 0.000154743 | -0.000603095 | -0.001566148 | 0.000168576 | -0.000422359 | -0.000730734 | 3.07E-05 | 0.000230519 | 6.23E-06 | -2.72E-05 | 7.95E-05 | -7.08E-06 | 1.80E-06 | -2.30E-05 | 1.38E-05 |
| PC70 | 0.001425942 | 0.001772087 | -0.00044936 | 0.000375304 | -0.001402326 | -0.000618802 | -0.000891445 | -0.000582935 | 0.000233607 | 0.000854643 | -0.000140227 | 1.20E-05 | 0.000506405 | 7.09E-05 | 3.79E-05 | 7.41E-05 | 0.000245811 | -0.000130077 | 3.95E-06 | 2.23E-05 | -2.25E-06 | -4.07E-05 |
| PC71 | 0.000315949 | -1.35E-05 | 5.47E-05 | 0.000645296 | 8.27E-05 | 6.67E-06 | 1.99E-05 | 6.32E-05 | -2.76E-05 | -0.000470999 | -2.55E-05 | -0.000107728 | -7.63E-05 | 6.84E-05 | 0.000220631 | -3.03E-05 | 1.26E-05 | 3.37E-05 | -1.16E-07 | 2.85E-05 | -1.76E-06 | -1.70E-05 |
| PC72 | 0.000300289 | -0.00047615 | 0.000412499 | -0.002083455 | 0.000776477 | 0.000926712 | 8.57E-05 | -0.00095204 | 0.000320624 | 0.000873012 | 7.96E-05 | 0.000287154 | 6.60E-05 | -8.25E-05 | -0.00036558 | 8.18E-05 | 1.07E-06 | -8.59E-05 | -2.28E-05 | -5.77E-05 | 2.01E-07 | 3.50E-05 |
| PC73 | -0.00072394 | 0.000313521 | -0.000413568 | 0.00209565 | -0.002219918 | -0.001069967 | 0.00028599 | 0.001076847 | -9.19E-05 | -0.000364063 | -0.000272882 | 0.000125755 | 0.000137238 | -0.000235453 | 0.000224037 | 3.52E-05 | -5.22E-05 | 9.64E-05 | 2.27E-05 | 5.01E-06 | 4.19E-05 | -2.27E-05 |
| PC74 | -0.000719644 | -0.000477239 | -6.37E-05 | -0.000158964 | 0.000449195 | 0.000889824 | 1.59E-05 | -6.67E-05 | 0.000290689 | -8.24E-05 | 0.000255601 | 0.000173318 | -0.000179996 | 2.60E-05 | -0.000105957 | -0.000112612 | -0.000114518 | 6.22E-05 | 5.46E-07 | 4.28E-05 | -4.32E-07 | 3.30E-05 |
| PC75 | 0.000695889 | 0.002356292 | 0.001457584 | -0.002289916 | -0.003051727 | 0.00082991 | 0.000338399 | 0.001991824 | 3.92E-05 | 0.000343419 | 0.000511146 | 0.00080279 | 0.000634877 | 8.61E-05 | -0.0001891 | -0.000473743 | 4.32E-05 | -7.28E-05 | 4.06E-05 | 5.97E-06 | 1.68E-05 | -5.10E-05 |
| PC76 | 0.002072237 | -0.003556588 | -0.0025737 | 0.003179303 | 0.005815267 | 0.000675031 | -0.001882207 | -0.003795638 | -0.00056382 | -0.000922816 | -0.000823909 | -0.00093921 | -0.000927387 | -0.000324074 | 0.000472187 | 0.000835933 | 1.80E-05 | 8.83E-05 | -2.77E-05 | -1.68E-06 | 1.48E-05 | 4.24E-05 |
| PC77 | -0.003865449 | 0.001673415 | 0.002915549 | -0.004506279 | -0.005221506 | -0.00066031 | -7.02E-05 | 0.002417682 | 0.000854948 | 0.000329905 | 2.63E-05 | 0.000813115 | 0.000271525 | 2.02E-05 | -0.000428345 | -0.000726924 | -0.00018689 | 0.000152684 | -4.48E-05 | 3.93E-05 | 1.11E-05 | 2.98E-05 |
| PC78 | 0.001934898 | -0.001646589 | -0.000445402 | 0.003553657 | 0.003721798 | -0.000275161 | -7.13E-05 | -0.000992637 | -0.000360064 | -0.000320204 | -6.57E-05 | -0.001031804 | -0.000477619 | 0.000252567 | 0.000575433 | 5.81E-05 | 0.00014108 | -2.47E-05 | -4.12E-05 | 8.29E-06 | -5.18E-05 | -4.01E-05 |
| PC79 | -0.000314763 | 0.001351512 | -0.0004598 | -0.001032252 | -0.00198655 | 0.000164819 | 0.00054598 | 0.0008803 | 0.00024929 | 0.000255773 | 0.000152153 | 0.00064243 | 0.000319488 | -0.000143324 | -0.000343145 | -0.000125084 | -4.65E-05 | -5.17E-05 | 2.83E-05 | -4.79E-06 | 3.17E-05 | -1.28E-05 |
| PC80 | -0.001047646 | -0.000379103 | -0.000411737 | 0.001007331 | 0.000606754 | -0.000236243 | 0.000749342 | 6.64E-05 | -9.48E-05 | 3.84E-05 | 0.000224954 | -5.26E-05 | 0.000226574 | 6.01E-05 | -0.000132 | 0.000122819 | 4.36E-05 | -5.75E-05 | 5.94E-05 | -4.50E-05 | -3.56E-05 | -1.69E-05 |
| PC81 | 0.002260547 | -0.000334401 | -0.000262812 | -0.001328681 | 0.000660487 | 0.000274151 | -0.0004942 | -0.000578837 | -6.49E-05 | 0.000393334 | 9.86E-05 | 1.73E-05 | 9.63E-05 | 8.13E-05 | 0.000166856 | 9.93E-05 | 2.04E-06 | 8.42E-06 | -7.45E-05 | -1.44E-06 | 3.73E-05 | 1.49E-05 |
| PC82 | -0.000761502 | 0.000854341 | 0.000494622 | 0.000906095 | -0.00076846 | -0.000493187 | -7.36E-05 | 0.000539684 | -0.000231252 | -0.000527708 | 2.62E-05 | -0.000146511 | -0.000241207 | -0.000153809 | 1.58E-05 | -0.000128701 | 4.65E-06 | 9.16E-08 | 5.45E-05 | -1.02E-05 | -2.66E-05 | -1.08E-05 |
| PC83 | 0.000578096 | -0.000213014 | -0.00035161 | 0.000673237 | -0.000662591 | -0.000374261 | -0.00021428 | -0.000108967 | 2.47E-06 | 0.000137149 | 0.000134702 | -0.000192215 | 0.000248144 | 0.000231671 | 0.000340099 | 1.62E-05 | 6.22E-05 | 3.83E-05 | -1.80E-05 | 6.24E-05 | 8.27E-06 | -2.96E-05 |
| PC84 | 0.00022873 | -0.000449059 | -0.000191986 | -0.000150849 | 0.001166923 | 0.00029715 | 0.00067973 | -0.000119453 | -7.06E-05 | 7.52E-05 | -0.0001659 | -0.000100816 | -2.60E-05 | 1.89E-05 | -0.000196406 | 9.28E-05 | -7.66E-05 | -2.63E-05 | 2.14E-05 | -4.99E-05 | -3.35E-05 | 1.59E-05 |
| PC85 | -0.000583072 | -0.000387903 | -1.08E-05 | -9.93E-05 | 0.000527937 | 0.000145634 | 0.000269626 | 6.73E-05 | 0.000143416 | 0.000258829 | 1.07E-05 | 4.12E-05 | 7.47E-05 | 0.000105978 | -0.000111312 | -4.90E-05 | -4.94E-05 | -3.39E-05 | -9.33E-06 | -1.15E-05 | -1.10E-05 | -1.20E-06 |

HPLC：

|  | Peak1 | Peak2 | Peak3 | Peak4 | Peak5 | Peak6 | Peak7 | Peak8 | Peak9 | Peak10 | Peak11 | Peak12 | Peak13 | Peak14 | Peak15 | Peak16 | Peak17 | Peak18 | Peak19 | Peak20 | Peak21 | Peak22 |
| --- | --- | --- | --- | --- | --- | --- | --- | --- | --- | --- | --- | --- | --- | --- | --- | --- | --- | --- | --- | --- | --- | --- |
| PC1 | 0.258171296 | -0.364515786 | 0.152880906 | -0.178347508 | 0.604128383 | 0.304194988 | -0.234619701 | 0.006016313 | 0.346377233 | -0.298835644 | 0.118520052 | -0.034144508 | 0.0693602 | 0.038056349 | -0.031973927 | -0.024898372 | -0.00824375 | 0.004566673 | -0.013457298 | -0.003965675 | 0.002446199 | -0.00461924 |
| PC2 | 0.163933118 | 0.426630109 | -0.339347161 | -0.501441414 | -0.396145525 | -0.001208804 | 0.111801344 | 0.316736869 | 0.172634593 | -0.190333096 | -0.204038301 | -0.152243662 | 0.08925626 | 0.080278821 | -0.068443558 | -0.054793518 | -0.014509079 | 0.036607798 | 0.01553031 | 0.004311899 | 0.002710761 | 0.001739601 |
| PC3 | 0.432136196 | -0.685287114 | 0.211138726 | 0.200134662 | -0.400740594 | 0.177279588 | -0.022995689 | 0.003102862 | 0.078557779 | 0.009807361 | 0.083424914 | 0.145642575 | 0.068618326 | 0.006372411 | -0.033138653 | 0.013152758 | 0.021408168 | 0.152988786 | 0.035979342 | 0.023652956 | 0.015555605 | 0.005278874 |
| PC4 | 0.424789417 | -0.657091128 | 0.021603733 | -0.108223875 | -0.482509858 | 0.179961811 | 0.061446778 | -0.131122712 | 0.103798909 | -0.147332839 | -0.083642598 | 0.098822935 | 0.055390638 | -0.11625506 | 0.058785985 | 0.017342501 | -0.055543642 | -0.116418358 | -0.052403305 | -0.016265476 | -0.012745614 | -0.006370186 |
| PC5 | 0.035433499 | -0.64570617 | 0.303775632 | -0.066295525 | 0.37129868 | -0.260933101 | 0.252135256 | 0.201056391 | -0.061324699 | 0.189551321 | -0.289825468 | -0.022493722 | 0.196879049 | -0.083501909 | 0.001640722 | -0.044262994 | -0.049613004 | 0.022273027 | -0.014784006 | 0.009944403 | -0.00472368 | 0.000538894 |
| PC6 | 0.620427221 | 0.13188837 | -0.151747211 | -0.042656781 | 0.010275315 | -0.085930833 | -0.139688477 | 0.609092157 | 0.225102127 | 0.227537794 | 0.182649224 | 0.148820324 | 0.006552811 | -0.0315602 | 0.084226697 | 0.086457656 | 0.010331134 | -0.031705553 | -0.011899055 | -0.003961505 | -0.000382655 | 0.001671381 |
| PC7 | 0.048013865 | -0.630081066 | 0.16811118 | 0.476343656 | -0.160608906 | -0.312959879 | -0.082618609 | 0.246257558 | 0.044763678 | -0.157056418 | 0.079283236 | -0.322915026 | -0.118028512 | 0.032231428 | -0.034052484 | 0.013706887 | -0.003724133 | -0.025769348 | 0.005983641 | -0.007103452 | -0.002739615 | 0.000975854 |
| PC8 | 0.877050265 | -0.057614757 | 0.054646842 | -0.224473931 | 0.039969519 | 0.199265604 | -0.048668679 | 0.077343947 | -0.171577107 | 0.019230614 | -0.038288792 | -0.14023003 | -0.163081629 | -0.153556347 | 0.10535467 | -0.060079184 | 0.036343782 | 0.010772241 | -0.036535387 | 0.042194111 | 0.063366059 | -0.006544903 |
| PC9 | 0.931549874 | 0.160410869 | -0.086362035 | 0.071020775 | -0.012569737 | 0.078864061 | -0.183349254 | 0.042571992 | -0.14505283 | 0.020420815 | 0.074766734 | -0.035471509 | 0.000164503 | -0.056956657 | -0.015529999 | -0.089757734 | -0.025547902 | 0.017358051 | 0.001468302 | 0.055344299 | -0.084925871 | -0.033091962 |
| PC10 | 0.834377944 | 0.210330577 | -0.166992786 | 0.257790158 | 0.008581734 | -0.217129935 | -0.141896967 | -0.064487123 | -0.014840198 | -0.07012407 | 0.012822557 | 0.096650843 | 0.165955702 | -0.057154906 | -0.178159516 | -0.043743662 | 0.026345911 | -0.08444973 | 0.055756303 | 0.041800203 | 0.04374734 | 0.004279021 |
| PC11 | 0.633912993 | -0.585074331 | 0.009212136 | -0.19852361 | -0.012908503 | 0.193549162 | -0.028717497 | 0.015197102 | -0.099345253 | 0.270112691 | -0.062096853 | 0.003586627 | -0.055118209 | 0.262392194 | -0.050826965 | -0.071913148 | 0.075303566 | -0.067052408 | 0.029258008 | -0.016614713 | -0.004351065 | 0.001687953 |
| PC12 | 0.651486816 | -0.104663888 | 0.132052017 | -0.15290288 | 0.133794759 | -0.227654282 | 0.310694054 | 0.173548806 | -0.314777177 | -0.389936944 | 0.089065328 | 0.220183469 | -0.095373417 | 0.097142466 | 0.032443652 | 0.00984302 | -0.009476472 | 0.003982481 | 0.00835573 | -0.000422665 | -0.002738777 | 0.001269391 |
| PC13 | 0.836103526 | 0.145339062 | 0.029145993 | 0.043529788 | 0.02911943 | 0.153870488 | -0.1834352 | -0.033249498 | -0.291951007 | -0.066159613 | -0.093184625 | -0.155545385 | 0.222363236 | 0.061497188 | 0.086749549 | 0.183072079 | 0.01662432 | 0.006689161 | 0.016787293 | -0.019836528 | 0.000472554 | -0.005477515 |
| PC14 | 0.913985461 | 0.235079136 | 0.059784681 | 0.1128508 | -0.003875804 | 0.043670425 | -0.134969233 | 0.057267514 | -0.130355698 | 0.0282993 | -0.008062264 | 0.02144562 | -0.022607832 | -0.072184303 | -0.149747397 | -0.05883984 | -0.021169697 | 0.042479436 | -0.086090298 | -0.096467482 | -0.002119108 | 0.027190832 |
| PC15 | 0.470557634 | 0.157486426 | -0.101441037 | 0.189896947 | 0.070452528 | 0.324149802 | 0.703865098 | 0.016265032 | 0.050074162 | 0.08220102 | 0.254839376 | -0.132594625 | 0.057969228 | -0.022520733 | -0.070987135 | 0.025339899 | 1.31E-05 | -0.018003082 | -0.004815746 | 0.002318788 | -0.000105252 | -0.002159374 |
| PC16 | 0.576826525 | 0.252261704 | 0.639392393 | -0.064277984 | 0.005689981 | -0.11568505 | 0.127494031 | -0.086537303 | 0.228532836 | -0.032196144 | -0.222794224 | 0.022193211 | -0.101846114 | -0.060709671 | -0.08278096 | 0.092855397 | 0.139251686 | 0.000675696 | -0.018564827 | 0.007134554 | -0.024327192 | -0.014712087 |
| PC17 | 0.318346982 | 0.282714459 | -0.241887576 | 0.695181985 | 0.106634505 | 0.249430194 | 0.082340697 | 0.114482682 | 0.130121283 | -0.084456227 | -0.354801305 | 0.091234145 | -0.059064883 | 0.0864833 | 0.108522988 | -0.046737702 | -0.014571502 | -0.000900389 | -0.004222108 | 0.003914383 | 0.003902658 | 0.002073213 |
| PC18 | 0.847825765 | 0.006466642 | -0.246868302 | -0.004683272 | 0.006266417 | -0.315451068 | 0.08065735 | -0.231996343 | 0.173478658 | 0.050146144 | 0.057838018 | -0.013804107 | 0.016779456 | 0.012852736 | 0.101433747 | -0.053749502 | -0.004846017 | 0.033276976 | 0.035078272 | -0.061976427 | 0.01858615 | -0.063162734 |
| PC19 | 0.417795099 | 0.335271984 | 0.798864744 | -0.076705989 | -0.037186263 | 0.133951633 | -0.020342295 | 0.02034967 | 0.051219579 | 0.061833431 | -0.022168124 | -0.019186065 | -0.085379018 | -0.037406937 | 0.015095449 | -0.00951067 | -0.114786519 | -0.022222875 | 0.135079443 | -0.020333632 | 0.002075901 | 0.013035879 |
| PC20 | 0.85302802 | -0.072695722 | -0.269923283 | -0.101323875 | 0.077573653 | -0.140066852 | -0.013602087 | -0.218415623 | 0.113123614 | 0.123131831 | -0.070901413 | -0.008607325 | -0.139024258 | 0.112629807 | -0.092214778 | 0.119413275 | -0.131527338 | 0.02158606 | -0.051642635 | 0.044787046 | 0.010517646 | 0.004685829 |
| PC21 | 0.096406383 | 0.454496559 | 0.793235693 | 0.086412016 | -0.18130926 | -0.124787026 | -0.015653639 | -0.060719036 | 0.057315631 | 0.032775095 | 0.139096578 | -0.017370889 | 0.135866968 | 0.167243354 | 0.087316252 | -0.074772792 | -0.018100397 | -0.011642245 | -0.099727064 | 0.028003466 | 0.014981533 | 0.000799738 |
| PC22 | 0.868519575 | -0.043687922 | -0.256447597 | -0.073976308 | 0.041620575 | -0.214187006 | 0.062686042 | -0.242610964 | 0.14572858 | -0.009820582 | 0.050854928 | -0.06651607 | 0.025724776 | -0.028078702 | 0.148327578 | -0.038452842 | 0.044352812 | 0.010093794 | 0.025194877 | 0.006054593 | -0.026400398 | 0.073607489 |

## Supplementary Table2

Sample information

| No. | Sample | Batch | Date of manufacture | Manufacturer | No. | Sample | Batch | Date of manufacture | Manufacturer |
| --- | --- | --- | --- | --- | --- | --- | --- | --- | --- |
| 1 | BS | 180311 | 2018.03 | Anhui People ’s Chinese Herbal Pieces Co., Ltd. | 68 | GE | / | 2019.06 | Chengdu lotus pond Chinese medicine market |
| 2 | BS | 181001 | 2018.10 | Anhui People ’s Chinese Herbal Pieces Co., Ltd. | 69 | GE | / | 2019.06 | Chengdu lotus pond Chinese medicine market |
| 3 | BS | 181202 | 2018.12 | Anhui People ’s Chinese Herbal Pieces Co., Ltd. | 70 | GE | / | 2019.06 | Chengdu lotus pond Chinese medicine market |
| 4 | BS | 20181201 | 2018.12 | Anhui Dechang Pharmaceutical Co., Ltd. | 71 | GE | / | 2019.06 | Chengdu lotus pond Chinese medicine market |
| 5 | BS | 170801 | 2017.08 | Bozhou Pioneer Chinese Herbal Pieces Co., Ltd. | 72 | GE | / | 2019.06 | Chengdu lotus pond Chinese medicine market |
| 6 | BS | 1901191 | 2019.01 | Anhui Puren Chinese Herbal Pieces Co., Ltd. | 73 | GE | / | 2019.06 | Chengdu lotus pond Chinese medicine market |
| 7 | BS | 1808061 | 2018.08 | Anhui Puren Chinese Herbal Pieces Co., Ltd. | 74 | GE | / | 2019.06 | Chengdu lotus pond Chinese medicine market |
| 8 | BS | 1812131 | 2018.12 | Anhui Puren Chinese Herbal Pieces Co., Ltd. | 75 | GE | / | 2019.06 | Chengdu lotus pond Chinese medicine market |
| 9 | BS | 1812230102 | 2018.12 | Bozhou Huqiao pharmaceutical Co., Ltd. | 76 | PO | / | 2019.06 | Chengdu lotus pond Chinese medicine market |
| 10 | BS | 181219 | 2018.12 | Anhui People ’s Chinese Herbal Pieces Co., Ltd. | 77 | PO | / | 2019.06 | Chengdu lotus pond Chinese medicine market |
| 11 | BS | 18051001 | 2018.05 | Bozhou Yonggang Decoction Pieces Factory Co., Ltd. | 78 | PO | / | 2019.06 | Chengdu lotus pond Chinese medicine market |
| 12 | BS | 18072501 | 2018.07 | Bozhou Yonggang Decoction Pieces Factory Co., Ltd. | 79 | PO | / | 2019.06 | Chengdu lotus pond Chinese medicine market |
| 13 | BS | 20181001 | 2018.10 | Anhui Dechang Pharmaceutical Co., Ltd. | 80 | PO | / | 2019.06 | Chengdu lotus pond Chinese medicine market |
| 14 | BS | D1812074 | 2018.12 | Sichuan Xinhehua Chinese Herbal Pieces Co., Ltd. | 81 | PO | / | 2019.06 | Chengdu lotus pond Chinese medicine market |
| 15 | BS | 180715 | 2018.07 | Anhui People ’s Chinese Herbal Pieces Co., Ltd. | 82 | PO | / | 2019.06 | Chengdu lotus pond Chinese medicine market |
| 16 | BS | 180903 | 2018.09 | Anhui People ’s Chinese Herbal Pieces Co., Ltd. | 83 | PO | / | 2019.06 | Chengdu lotus pond Chinese medicine market |
| 17 | BS | 190213 | 2019.02 | Anhui People ’s Chinese Herbal Pieces Co., Ltd. | 84 | PO | / | 2019.06 | Chengdu lotus pond Chinese medicine market |
| 18 | BS | 181125 | 2018.11 | Anhui People ’s Chinese Herbal Pieces Co., Ltd. | 85 | PO | / | 2019.06 | Chengdu lotus pond Chinese medicine market |
| 19 | BS | 181216 | 2018.12 | Anhui People ’s Chinese Herbal Pieces Co., Ltd. | 86 | PO | / | 2019.06 | Chengdu lotus pond Chinese medicine market |
| 20 | BS | 190113 | 2019.01 | Anhui People ’s Chinese Herbal Pieces Co., Ltd. | 87 | PO | / | 2019.06 | Chengdu lotus pond Chinese medicine market |
| 21 | BS | 180520 | 2018.05 | Anhui People ’s Chinese Herbal Pieces Co., Ltd. | 88 | PO | / | 2019.06 | Chengdu lotus pond Chinese medicine market |
| 22 | BS | 190120 | 2019.01 | Anhui People ’s Chinese Herbal Pieces Co., Ltd. | 89 | PO | / | 2019.06 | Chengdu lotus pond Chinese medicine market |
| 23 | BS | 181110 | 2018.11 | Anhui People ’s Chinese Herbal Pieces Co., Ltd. | 90 | PO | / | 2019.06 | Chengdu lotus pond Chinese medicine market |
| 24 | BS | 181005 | 2018.10 | Anhui People ’s Chinese Herbal Pieces Co., Ltd. | 91 | PO | / | 2019.06 | Chengdu lotus pond Chinese medicine market |
| 25 | BS | 190214 | 2019.02 | Anhui People ’s Chinese Herbal Pieces Co., Ltd. | 92 | PO | / | 2019.06 | Chengdu lotus pond Chinese medicine market |
| 26 | BS | 180707 | 2018.07 | Anhui People ’s Chinese Herbal Pieces Co., Ltd. | 93 | PO | / | 2019.06 | Chengdu lotus pond Chinese medicine market |
| 27 | BS | 190101 | 2019.01 | Anhui People ’s Chinese Herbal Pieces Co., Ltd. | 94 | PO | / | 2019.06 | Chengdu lotus pond Chinese medicine market |
| 28 | BS | 18030102 | 2018.03 | Zhengzhou Ruilong Pharmaceutical Co., Ltd. | 95 | PO | / | 2019.05 | Yikangtang of Chengdu lotus pond Chinese medicine market |
| 29 | BS | 18030101 | 2018.03 | Zhengzhou Ruilong Pharmaceutical Co., Ltd. | 96 | PO | / | 2019.05 | Yikangtang of Chengdu lotus pond Chinese medicine market |
| 30 | BS | / | 2019.06 | Chengdu lotus pond Chinese medicine market | 97 | PO | / | 2019.05 | Yikangtang of Chengdu lotus pond Chinese medicine market |
| 31 | BS | / | 2019.06 | Chengdu lotus pond Chinese medicine market | 98 | PO | / | 2019.05 | Yikangtang of Chengdu lotus pond Chinese medicine market |
| 32 | BS | / | 2019.05 | Yikangtang of Chengdu lotus pond Chinese medicine market | 99 | PO | / | 2019.05 | Yikangtang of Chengdu lotus pond Chinese medicine market |
| 33 | BS | / | 2019.05 | Yikangtang of Chengdu lotus pond Chinese medicine market | 100 | PO | / | 2019.05 | Yikangtang of Chengdu lotus pond Chinese medicine market |
| 34 | BS | / | 2019.05 | Yikangtang of Chengdu lotus pond Chinese medicine market | 101 | PO | / | 2019.05 | Yikangtang of Chengdu lotus pond Chinese medicine market |
| 35 | BS | / | 2019.05 | Yikangtang of Chengdu lotus pond Chinese medicine market | 102 | PO | / | 2019.05 | Yikangtang of Chengdu lotus pond Chinese medicine market |
| 36 | BS | / | 2019.05 | Yikangtang of Chengdu lotus pond Chinese medicine market | 103 | PO | / | 2019.05 | Yikangtang of Chengdu lotus pond Chinese medicine market |
| 37 | BS | / | 2019.05 | Yikangtang of Chengdu lotus pond Chinese medicine market | 104 | PO | / | 2019.05 | Yikangtang of Chengdu lotus pond Chinese medicine market |
| 38 | BS | / | 2019.05 | Yikangtang of Chengdu lotus pond Chinese medicine market | 105 | PO | / | 2019.05 | Yikangtang of Chengdu lotus pond Chinese medicine market |
| 39 | BS | / | 2019.05 | Yikangtang of Chengdu lotus pond Chinese medicine market | 106 | BOS | / | 2019.05 | Yikangtang of Chengdu lotus pond Chinese medicine market |
| 40 | BS | / | 2019.05 | Yikangtang of Chengdu lotus pond Chinese medicine market | 107 | BOS | / | 2019.05 | Yikangtang of Chengdu lotus pond Chinese medicine market |
| 41 | BS | / | 2019.05 | Yikangtang of Chengdu lotus pond Chinese medicine market | 108 | BOS | / | 2019.05 | Yikangtang of Chengdu lotus pond Chinese medicine market |
| 42 | BS | / | 2019.05 | Yikangtang of Chengdu lotus pond Chinese medicine market | 109 | BOS | / | 2019.05 | Yikangtang of Chengdu lotus pond Chinese medicine market |
| 43 | BS | / | 2019.05 | Yikangtang of Chengdu lotus pond Chinese medicine market | 110 | BOS | / | 2019.05 | Yikangtang of Chengdu lotus pond Chinese medicine market |
| 44 | BS | / | 2019.05 | Yikangtang of Chengdu lotus pond Chinese medicine market | 111 | BOS | / | 2019.05 | Yikangtang of Chengdu lotus pond Chinese medicine market |
| 45 | BS | / | 2019.05 | Yikangtang of Chengdu lotus pond Chinese medicine market | 112 | BOS | / | 2019.05 | Yikangtang of Chengdu lotus pond Chinese medicine market |
| 46 | GE | / | 2019.05 | Yikangtang of Chengdu lotus pond Chinese medicine market | 113 | BOS | / | 2019.05 | Yikangtang of Chengdu lotus pond Chinese medicine market |
| 47 | GE | / | 2019.05 | Yikangtang of Chengdu lotus pond Chinese medicine market | 114 | BOS | / | 2019.05 | Yikangtang of Chengdu lotus pond Chinese medicine market |
| 48 | GE | / | 2019.05 | Yikangtang of Chengdu lotus pond Chinese medicine market | 115 | BOS | / | 2019.05 | Yikangtang of Chengdu lotus pond Chinese medicine market |
| 49 | GE | / | 2019.05 | Yikangtang of Chengdu lotus pond Chinese medicine market | 116 | BOS | / | 2019.05 | Yikangtang of Chengdu lotus pond Chinese medicine market |
| 50 | GE | / | 2019.05 | Yikangtang of Chengdu lotus pond Chinese medicine market | 117 | BOS | / | 2019.05 | Yikangtang of Chengdu lotus pond Chinese medicine market |
| 51 | GE | / | 2019.05 | Yikangtang of Chengdu lotus pond Chinese medicine market | 118 | BOS | / | 2019.05 | Yikangtang of Chengdu lotus pond Chinese medicine market |
| 52 | GE | / | 2019.05 | Yikangtang of Chengdu lotus pond Chinese medicine market | 119 | BOS | / | 2019.05 | Yikangtang of Chengdu lotus pond Chinese medicine market |
| 53 | GE | / | 2019.05 | Yikangtang of Chengdu lotus pond Chinese medicine market | 120 | BOS | / | 2019.05 | Yikangtang of Chengdu lotus pond Chinese medicine market |
| 54 | GE | / | 2019.05 | Yikangtang of Chengdu lotus pond Chinese medicine market | 121 | BOS | / | 2019.05 | Yikangtang of Chengdu lotus pond Chinese medicine market |
| 55 | GE | / | 2019.05 | Yikangtang of Chengdu lotus pond Chinese medicine market | 122 | BOS | / | 2019.05 | Yikangtang of Chengdu lotus pond Chinese medicine market |
| 56 | GE | / | 2019.05 | Yikangtang of Chengdu lotus pond Chinese medicine market | 123 | BOS | / | 2019.05 | Yikangtang of Chengdu lotus pond Chinese medicine market |
| 57 | GE | / | 2019.05 | Yikangtang of Chengdu lotus pond Chinese medicine market | 124 | BOS | / | 2019.05 | Yikangtang of Chengdu lotus pond Chinese medicine market |
| 58 | GE | / | 2019.05 | Yikangtang of Chengdu lotus pond Chinese medicine market | 125 | BOS | / | 2019.05 | Yikangtang of Chengdu lotus pond Chinese medicine market |
| 59 | GE | / | 2019.05 | Yikangtang of Chengdu lotus pond Chinese medicine market | 126 | BOS | / | 2019.05 | Yikangtang of Chengdu lotus pond Chinese medicine market |
| 60 | GE | / | 2019.05 | Yikangtang of Chengdu lotus pond Chinese medicine market | 127 | BOS | / | 2019.05 | Yikangtang of Chengdu lotus pond Chinese medicine market |
| 61 | GE | / | 2019.05 | Yikangtang of Chengdu lotus pond Chinese medicine market | 128 | BOS | / | 2019.05 | Yikangtang of Chengdu lotus pond Chinese medicine market |
| 62 | GE | / | 2019.05 | Yikangtang of Chengdu lotus pond Chinese medicine market | 129 | BOS | / | 2019.05 | Yikangtang of Chengdu lotus pond Chinese medicine market |
| 63 | GE | / | 2019.05 | Yikangtang of Chengdu lotus pond Chinese medicine market | 130 | BOS | / | 2019.05 | Yikangtang of Chengdu lotus pond Chinese medicine market |
| 64 | GE | / | 2019.05 | Yikangtang of Chengdu lotus pond Chinese medicine market | 131 | BOS | / | 2019.05 | Yikangtang of Chengdu lotus pond Chinese medicine market |
| 65 | GE | / | 2019.05 | Yikangtang of Chengdu lotus pond Chinese medicine market | 132 | BOS | / | 2019.05 | Yikangtang of Chengdu lotus pond Chinese medicine market |
| 66 | GE | / | 2019.05 | Yikangtang of Chengdu lotus pond Chinese medicine market | 133 | BOS | / | 2019.05 | Yikangtang of Chengdu lotus pond Chinese medicine market |
| 67 | GE | / | 2019.05 | Yikangtang of Chengdu lotus pond Chinese medicine market | 134 | BOS | / | 2019.05 | Yikangtang of Chengdu lotus pond Chinese medicine market |

(BS: *Bletilla striata*; GE: *Gastrodia elata*; PO: *Polygonatum odoratum*; BOS: *Bletilla ochracea schltr*. “/” indicates the sample was purchased from Chinese medicine market and the corresponding pieces were self-processed. The shelf life of samples was 5 years.)
